# Synthesis and evaluation of new guanidine-thiourea organocatalyst for the nitro-Michael reaction: Theoretical studies on mechanism and enantioselectivity

**DOI:** 10.3762/bjoc.8.168

**Published:** 2012-09-07

**Authors:** Tatyana E Shubina, Matthias Freund, Sebastian Schenker, Timothy Clark, Svetlana B Tsogoeva

**Affiliations:** 1 Computer Chemistry Center and Interdisciplinary Center for Molecular Materials, University of Erlangen-Nuremberg, Nägelsbachstr. 25, 91052 Erlangen, Germany; 2 Department of Chemistry and Pharmacy, Chair of Organic Chemistry I, University of Erlangen-Nuremberg, Henkestraße 42, 91054, Erlangen, Germany

**Keywords:** bifunctional organocatalyst, DFT calculations, guanidine-thiourea, Michael addition, organocatalysis, transition states

## Abstract

A new guanidine-thiourea organocatalyst has been developed and applied as bifunctional organocatalyst in the Michael addition reaction of diethyl malonate to *trans*-β-nitrostyrene. Extensive DFT calculations, including solvent effects and dispersion corrections, as well as ab initio calculations provide a plausible description of the reaction mechanism.

## Introduction

In recent years bifunctional compounds have found frequent applications as organocatalysts in modern synthetic organic chemistry [1–8]. Over the past decade, different catalytic methodologies have been reported that use chiral thiourea-based bifunctional molecules [9–13]. In particular, remarkable progress has been made in the development of secondary and tertiary amine-thiourea bifunctional organocatalysts for a great number of useful transformations [14–35].

Recently, the Tsogoeva group and that of Jacobsen reported the first successful application of primary amine-thiourea organocatalysts with the synchronous dual activation of a nucleophile and an electrophile in nitro-Michael addition reactions [36–42]. Bifunctional organocatalysts that contain both a thiourea moiety and an imidazole group [43–44] on a chiral scaffold, as asymmetric catalysts in the addition of acetone to *trans*-β-nitrostyrene, have also been reported [44–47].

Since guanidines [48] are stronger bases than amines and/or imidazole, we were interested in exploring whether guanidine-thiourea organocatalysts would perform as well as or even better than amine-thioureas and imidazole-thioureas. Generally, guanidines are well-known basic catalysts in organic synthesis, but only scattered examples of chiral guanidines as organocatalysts are known [49]. Indeed, only one guanidine-thiourea organocatalyst has been published up until now [50–53]. This encouraged us to synthesize and investigate the potential of new guanidine-thiourea **7** as organocatalyst for the nitro-Michael addition reactions. Here we report the first results of our investigations, accompanied by quantum-chemical calculations on the mechanism and the observed stereoselectivity.

## Results and Discussion

### Synthesis and application of new guanidine-thiourea catalyst **7**

**Scheme 1:**
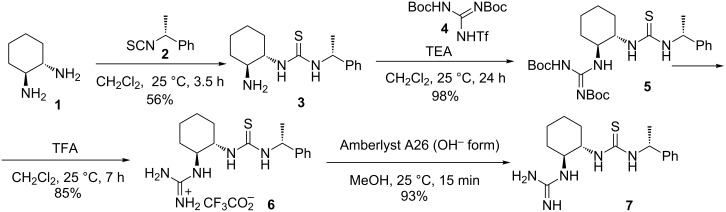
Synthesis of guanidine-thiourea organocatalyst **7**.

The syntheses of new guanidine-thiourea compound **7** was accomplished by known methods [36,38,40,54–56] as summarised in Scheme 1. (*S*,*S*)-1,2-Diaminocyclohexane (**1**) and (*R*)-1-phenylethyl isothiocyanate (**2**) were employed for the synthesis of primary amine-thiourea **3** [36,38,40,54]. Subsequent treatment of **3** with a guanidinylation reagent, *N*,*N′*-di-Boc-*N″*-triflylguanidine (**4**) [54–55], gave the intermediate **5** in 98% yield. The next step involved cleavage of the *tert*-butyl groups with trifluoroacetic acid (TFA) to give the corresponding salt **6** in 85% yield. Finally, guanidinium salt neutralisation with Amberlyst A26 (OH^−^ form) [56], filtration and evaporation afforded the guanidine-thiourea **7** in high yield and purity.

**Scheme 2:**
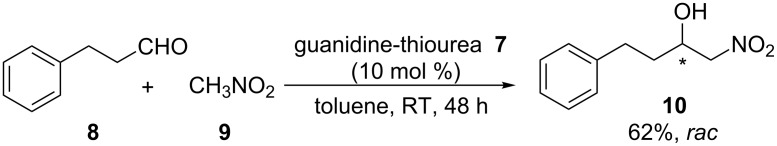
Henry reaction of 3-phenylpropionaldehyde (**8**) with nitromethane (**9**).

This compound was then examined for its ability to mediate the enantioselective C–C bond-formation reactions. As an initial model transformation we studied the Henry reaction of 3-phenylpropionaldehyde (**8**) with nitromethane (**9**) in the presence of 10 mol % of **7**, with the reaction proceeding for 48 h at room temperature in toluene. However, guanidine-thiourea **7** gave the product **10** only in racemic form and in moderate yield (62%, Scheme 2).

**Scheme 3:**
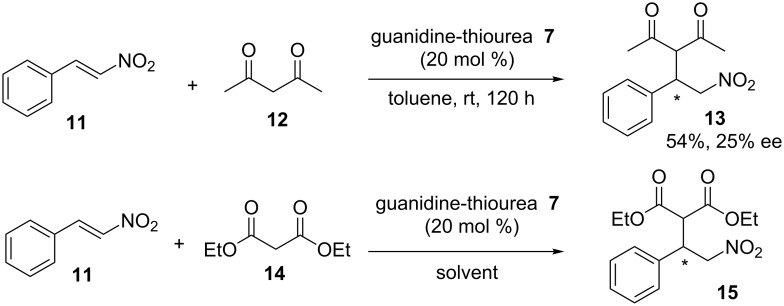
Michael addition of (**12**) and (**14**) to *trans*-β-nitrostyrene (**11**).

The Michael additions of 2,4-pentanedione and diethylmalonate to *trans*-β-nitrostyrene were further explored (Scheme 3). The use of guanidine-thiourea **7** at 20 mol % in toluene at room temperature resulted in the formation of the corresponding products **13** and **15** in moderate yields and low enantioselectivities, i.e., 54%, 25% ee for **13** (Scheme 3) and 66%, 5% ee for **15** (Scheme 3, Table 1, entry 1). Interestingly, while catalyst **7** provides the product **13** in 54% yield after 120 h, the same catalyst produces the Michael product **15** with 66% yield in only 2 h. We therefore decided to study the solvent effects on the reaction outcome in the nitro-Michael reaction of diethylmalonate with *trans*-β-nitrostyrene further. The results are shown in Table 1. Whereas Michael reactions performed in dichloromethane and ethyl acetate showed better results in terms of yields, compared to the results obtained in toluene (Table 1, entries 2 and 3 versus entry 1), reactions in ether and ethanol gave the adduct with lower yields (Table 1, entries 4 and 5). Notably, the highest yield (96%, Table 1, entry 6) was observed in THF. However, the Michael product was nearly racemic in all runs, indicating that the influence of chirality of the catalyst was minimal in all of the solvents screened. We expected that the resulting ee value would be higher at a lower temperature. However, carrying out the reaction for 24 h in THF at −78 °C gave again the racemic product in 78% yield. Hence, yield rather than ee value is influenced here by the reaction temperature. In order to explain the enantioselectivities observed, as well as to refine the catalyst design, or possibly get ideas that can be transferred to other systems, we decided to carry out a computational investigation of this guanidine-thiourea catalysed nitro-Michael reaction employing density functional calculations.

**Table 1:** Screening of solvents for the guanidine-thiourea **7** catalysed *nitro*-Michael addition of diethylmalonate (**14**) to *trans*-β-nitrostyrene (**11**) (Scheme 3).

entry	temperature	solvent	reaction time [h]	yield [%]^a^

1	rt	toluene	2	66
2	rt	CH_2_Cl_2_	2	81
3	rt	EtOAc	2	83
4	rt	Et_2_O	2	42
5	rt	EtOH	2	56
6	**rt**	**THF**	**2**	**96**
7	−78 °C	THF	24	78

^a^Yield of isolated product after column chromatography on SiO_2_.

### Theoretical studies: DFT calculations

**Figure 1:**
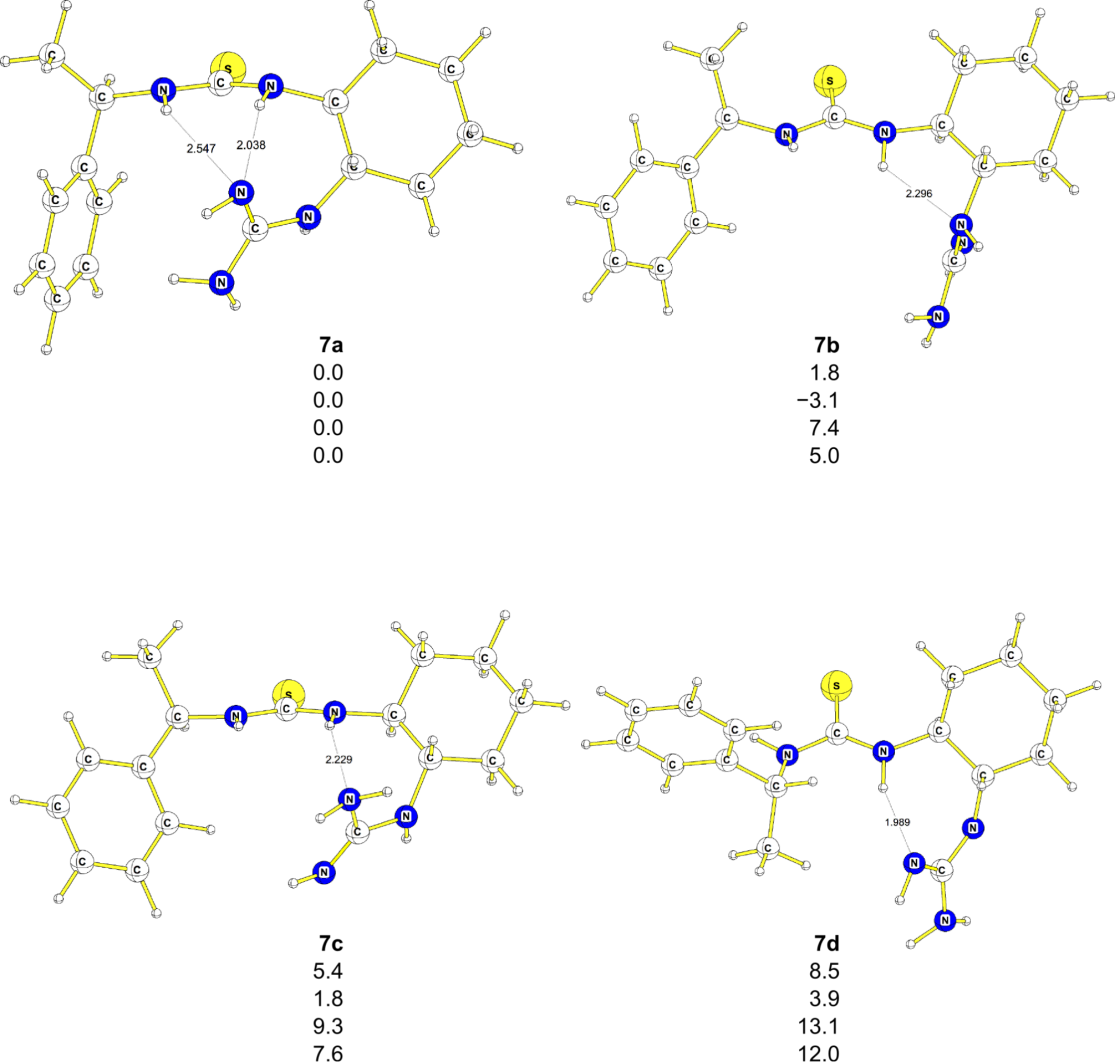
Optimized geometries of four conformers of catalyst **7**. Energies are in kcal·mol^−1^, B3PW91/6–31G(d) (first entry), DFT-PCM (second entry), MP2/6–311++G(d,p)// B3PW91/6–31G(d) (third entry), DFT-D (fourth entry). Bond lengths are in Å.

The main goal of our calculations was to gain insight into the mechanism of the nitro-Michael addition of diethyl malonate (**14**) to nitrostyrene (**11**) and to find a plausible explanation as to why the enantioselectivity of this reaction is low. As a first step, we explored the conformational flexibility of the catalyst **7** itself. Pápai and co-workers [57] studied a similar dimethylaminothiourea catalyst and found that their system cannot be treated as conformationally rigid. Thus, a full conformational search of **7** was performed at the AM1 level by using the TORQUE algorithm within the VAMP program [58]. Based on the clustered semiempirical results, the four most stable structures were selected for the further optimization at the B3PW91/6–31G(d) level. Conformer **7a** is the only one with two hydrogen bonds between a nitrogen atom in the guanidine moiety and hydrogen atoms from the thiourea fragment. While inclusion of the solvent effect (DFT-PCM) gives a preference of 3.1 kcal·mol^−1^ for **7b** over **7a** (Figure 1), gas-phase DFT, MP2 and DFT-D with van der Waals correction predict **7a** to be more stable than **7b** by between 1.8 and 7.4 kcal·mol^−1^ (Figure 1). The MP2 and DFT-D energies agree very well. The two other conformers, **7c** and **7d**, are less stable than **7a**/**7b** at all levels employed and were therefore not considered for the further studies.

**Scheme 4:**
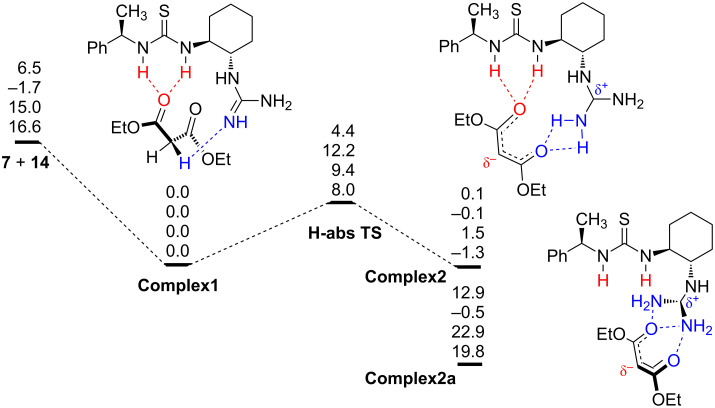
Energy profile for the first step of the reaction between catalyst **7** and malonate **14**. Energies are in kcal·mol^−1^, B3PW91/6–31G(d) (first entry), DFT-PCM (second entry), MP2/6–311++G(d,p)//B3PW91/6–31G(d) (third entry), DFT-D (fourth entry).

A similar Michael reaction of 1,3-dicarbonyl compounds with nitroolefins has been studied in some detail before [57,59]. It is generally proposed that the reaction proceeds first by deprotonation of the acidic proton of malonate **14** followed by formation of a complex between **7** and **14**. Formation of **Complex1** between catalyst **7** and malonate **14** is exothermic (−6.5, −15.0 and −16.6 kcal·mol^–1^ B3PW91/6–31G(d), MP2 and DFT-D, respectively, Scheme 4). Two energetically almost equivalent complexes **Complex1** and **Complex2** are connected by the H-abstraction transition state with a rather low activation barrier of 4.4 kcal·mol^−1^ at the B3PW91/6–31G(d) level. Inclusion of PCM, MP2 and DFT-D corrections increases the activation barrier to 12.2, 9.4 and 8.0 kcal·mol^−1^, in the DFT-PCM, MP2 and DFT-D results, respectively. We were also able to find a second complex **Complex2a**, which is less stable than **Complex2** by up to 22.9 kcal·mol^−1^ (MP2, Scheme 4). Again, gas-phase DFT, MP and DFT-D results follow the same trend, while inclusion of the solvent effect makes **Complex2a** 0.4 kcal·mol^−1^ more stable than **Complex2** (Scheme 4). In this complex, malonate **14** is coordinated only to the guanidine moiety.

**Figure 2:**
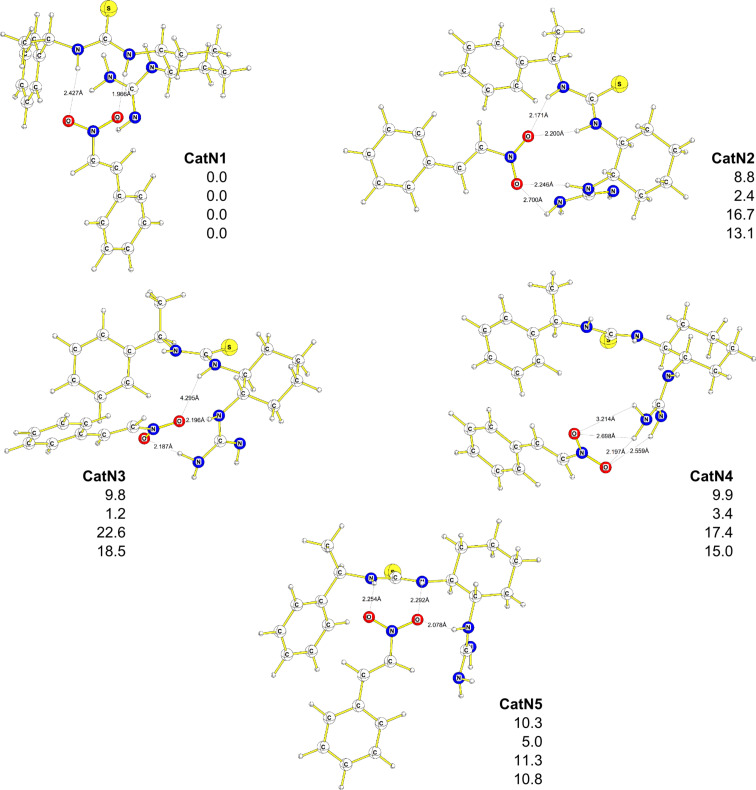
Complexes (**CatN1**–**CatN5**) between catalyst **7** and nitrostyrene **11**. Energies are in kcal·mol^−1^, B3PW91/6–31G(d) (first entry), DFT-PCM (second entry), MP2/6–311++G(d,p)// B3PW91/6–31G(d) (third entry), DFT-D (fourth entry). Bond lengths are in Å.

Since catalyst **7** has a certain degree of conformational flexibility, one can expect various complexes between the catalyst **7** and nitrostyrene (**11**) to exist. We were able to locate five complexes (Figure 2) that differ in energy by up to 10.3 kcal·mol^−1^ {5.0 kcal·mol^−1^ (DFT–PCM), 22.6 kcal·mol^−1^, (MP2), 18.5 kcal·mol^−1^, (DFT-D)}.

**Scheme 5:**
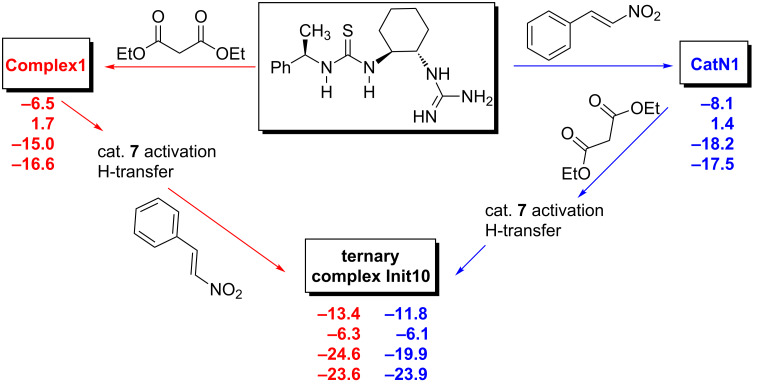
Two possible routes for ternary complex formation. Energies are in kcal·mol^−1^, B3PW91/6–31G(d) (first entry), DFT-PCM (second entry), MP2/6–311++G(d,p)//B3PW91/6–31G(d) (third entry), DFT-D (fourth entry).

It is interesting to note that nitrostyrene can coordinate not only to the thiourea moiety, as originally suggested by Takemoto and co-workers [22], but also to the amino group (as pointed out by Pápai and co-workers [57]) or both. The ternary complex **Init10** between catalyst **7**, malonate **14** and nitrostyrene (**11**) can be formed via two routes (Scheme 5): Formation of **Complex1**, activation of catalyst and H-transfer followed by addition of nitrostyrene (**11**) to **Complex2** or addition of malonate **14** to the complex **CatN1** followed by catalyst activation and H-transfer. The thermodynamics of both routes are comparable at all levels. Once ternary complex **Init10** is formed, there are few C–C bond-forming pathways.

**Figure 3:**
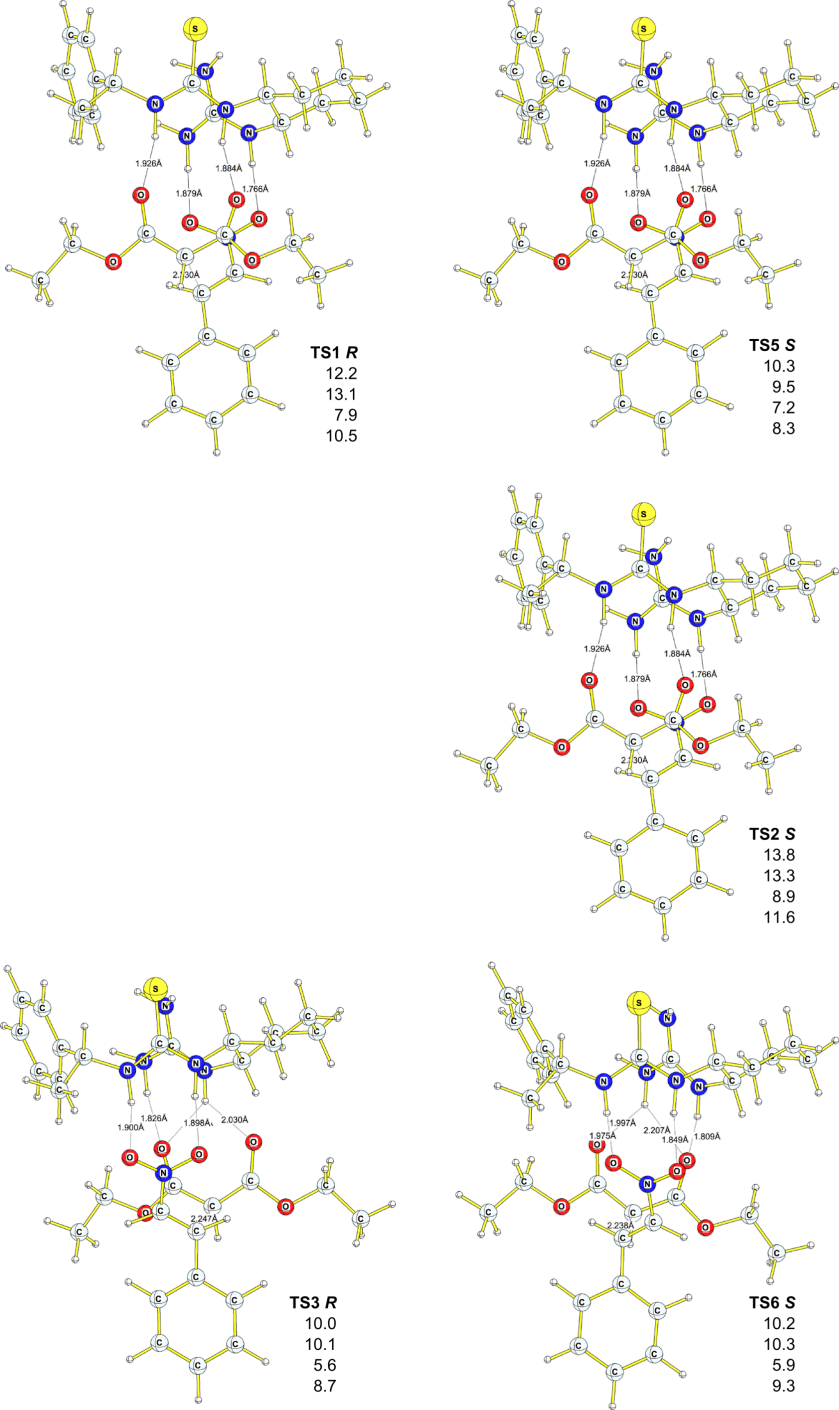
Geometries of transition states for *R* and *S* products. Relative energies (with respect to **Init10**) are in kcal·mol^−1^, B3PW91/6–31G(d) (first entry), DFT-PCM (second entry), MP2/6–311++ G(d,p)//B3PW91/6–31G(d) (third entry), DFT-D (fourth entry). Bond lengths are in Å.

**Figure 4:**
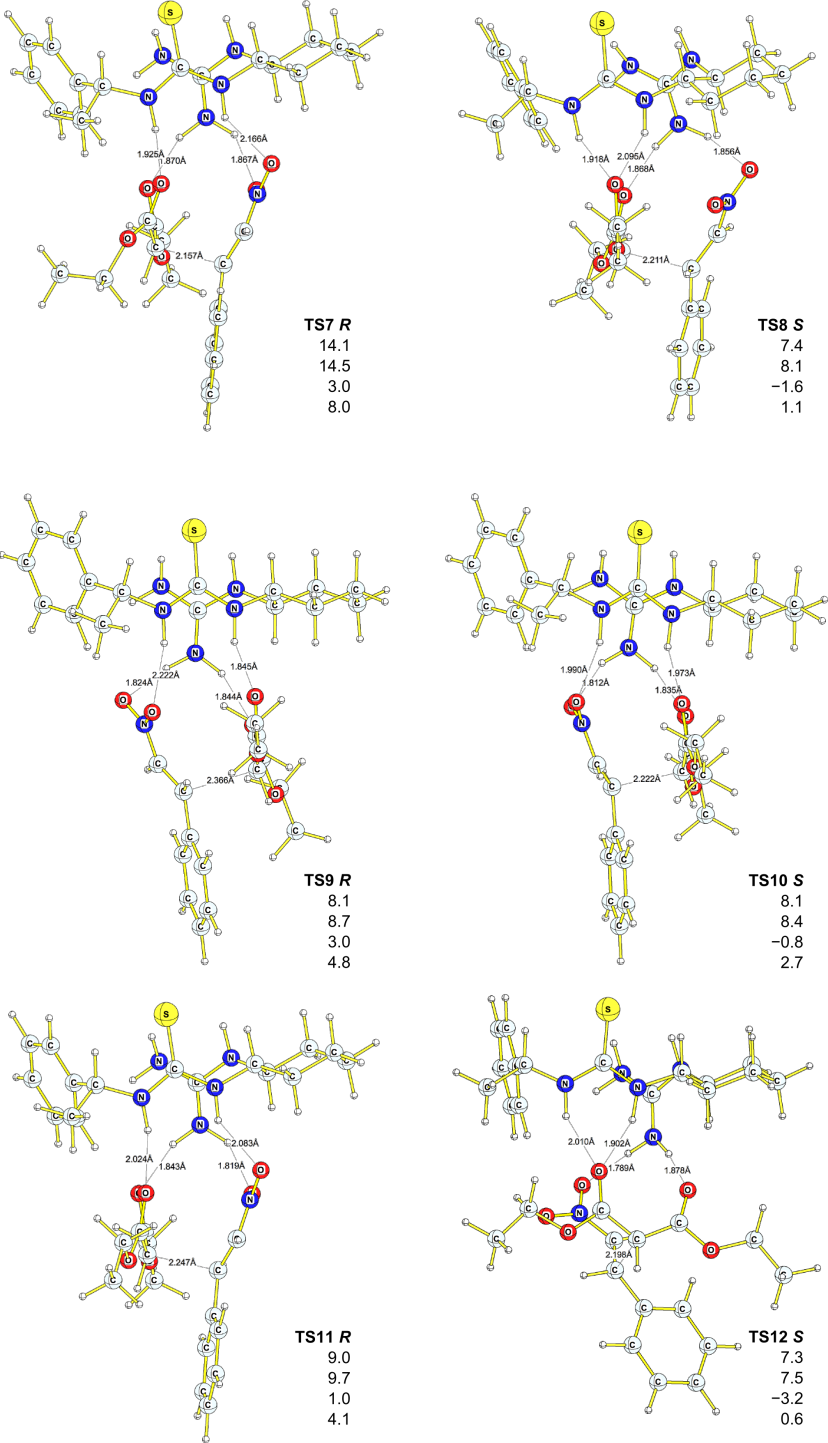
Geometries of transition states for *R* and *S* products. Relative energies (with respect to **Init10**) are in kcal·mol^−1^, B3PW91/6–31G(d) (first entry), DFT-PCM (second entry), MP2/6–311++ G(d,p)//B3PW91/6–31G(d) (third entry), DFT-D (fourth entry). Bond lengths are in Å.

Based on the extensive conformational search, we were able to find eleven competing transition states (Figure 3 and Figure 4), six of which lead to *R* products (**TS1**, **TS3**, **TS7**, **TS9**, **TS11, TS12**), and five of which (**TS2**, **TS5**, **TS6**, **TS8**, **TS10**) lead to *S* products; the corresponding initial and final complexes were also located. All but one (**TS6 *S***) transition state for the *S* enantiomer were found to lay lower on the potential-energy surface (PES), at all levels employed, compared to the corresponding transition states that lead to the *R* product. The activation barriers vary between +7.3 kcal·mol^−1^ (+7.5 kcal·mol^−1^ DFT-PCM, **TS8** ***S*)** and +14.1 kcal·mol^−1^ (+14.5 kcal·mol^−1^ DFT-PCM, **TS7** ***R***). DFT-D and MP2/6–311++G(d,p)//6–31G(d) results support the same trend, although the absolute values of the activation energies are much lower and in some cases even negative activation barriers were found (Figure 3 and Figure 4, Table 2). Relatively low activation barriers could explain the poor selectivity observed experimentally for this reaction, i.e., catalyst **7** is too active. The computed ee based on the theory of the activated complex (see Supporting Information File 1) give values between 43% and 99%, significantly higher than those observed experimentally. Also, at least six initial complexes (**INIT7**–**INIT12**) can interconvert, as the energy difference between them ranges from 0.7 kcal·mol^−1^ to 4.3 kcal·mol^−1^ (B3PW91/6–31G(d), Table 2).

**Table 2:** Computed energy difference between initial and final complexes and activation barriers with respect to the **Init10** complex. Energies are in kcal·mol^−1^, B3PW91/6–31G(d), (DFT-PCM), {MP2/6–311++G(d,p)//B3PW91/6–31G(d)} and [DFT-D].

	INIT	TS	FIN

**1** ***R***	10.7 (10.9) {8.8} [10.4]	12.2 (13.1) {7.9} [10.5]	1.7 (−1.0) {−10.6} [−2.5]
**5 *S***	8.9 (7.3) {9.7} [10.1]	10.3 (9.5) {7.2} [8.3]	−0.2 (−2.9) {−12.8} [−4.9]
			
**2** ***S***	8.5 (8.7) {7.8} [10.5]	13.8 (13.3) {8.9} [11.6]	3.0 (1.6) {−9.0} [−3.3]
			
**3** ***R***	7.0 (5.6) {9.5} [10.8]	10.0 (10.1) {5.6} [8.7]	1.8 (−0.7) {−11.2} [−3.7]
**6** ***S***	7.5 (6.4) {8.4} [9.5]	10.2 (10.3) {5.9} [9.3]	1.9 (0.1) {−12.2} [−5.0]
			
**7** ***R***	4.3 (4.9) {3.9} [4.7]	14.1 (14.5) {3.0} [8.0]	5.4 (3.9) {−11.9} [−4.6]
**8** ***S***	1.0 (1.5) {−0.4} [0.7]	7.4 (8.1) {−1.6} [1.1]	−5.7 (−6.8) {−21.3} [−13.8]
			
**9** ***R***	1.1 (0.9) {0.5} [1.6]	8.1 (8.7) {3.0} [4.8]	−5.9 (−6.6) {−20.5} [−13.2]
**10** ***S***	0.0 (0.0) {0.0} [0.0]	8.1 (8.4) {−0.8} [2.7]	−4.5 (−5.2) {−14.1} [−8.7]
			
**11** ***R***	0.7 (1.4) {0.4} [1.4]	9.0 (9.7) {1.0} [4.1]	−1.4 (−3.4) {−16.8} [−9.4]
			
**12** ***R***	1.3 (1.0) {−0.2} [1.2]	7.3 (7.5) {−3.2} [0.6]	−5.9 (−6.6) {−20.5} [−13.2]

In such a case (i.e., low barriers that result in thermodynamic control of the reaction products), the course of the reaction will be determined by the stability of the final *R* and *S* products. The most stable conformations of the final *R* and *S* complexes are essentially equally stable (Table 2). Thus, the experimentally observed low ee can be explained by the high catalytic activity of **7**. To lower its activity and to increase the selectivity of the reaction one can modify catalyst **7**, either by modifying a substituent, e.g., by introducing the bulky groups (*t-*Bu) into the phenyl ring, or by modifying the guanidine moiety. Preliminary semiempirical calculations suggest that the introduction of two *t-*Bu-groups into the 1,3- aryl positions would not improve the performance of the catalyst; the system remains too flexible. On the other hand, modification of the guanidine moiety by introducing a rigid aza-heterocycle (1,5,7-triazabicyclo[4.4.0]dec-5-ene (TABD)) gave promising results.

**Figure 5:**
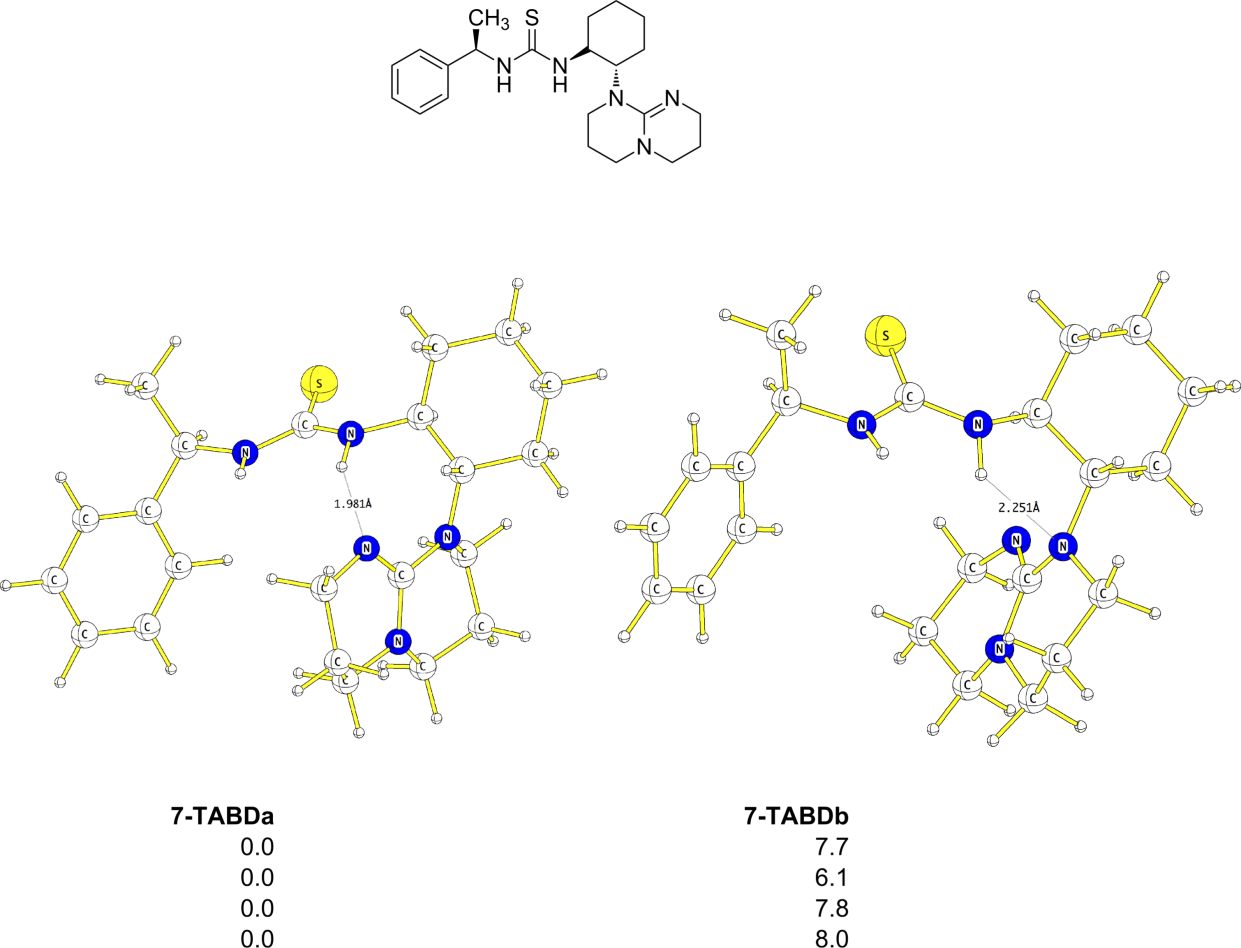
B3PW91/6–31G(d) (first entry), DFT-PCM (second entry), MP2/6–31G(d)//B3PW91/6–31G(d) (third entry) and DFT-D (fourth entry) optimized geometries of modified **7-TABD** catalyst. Energies are in kcal·mol^−1^, bond lengths are in Å.

**Figure 6:**
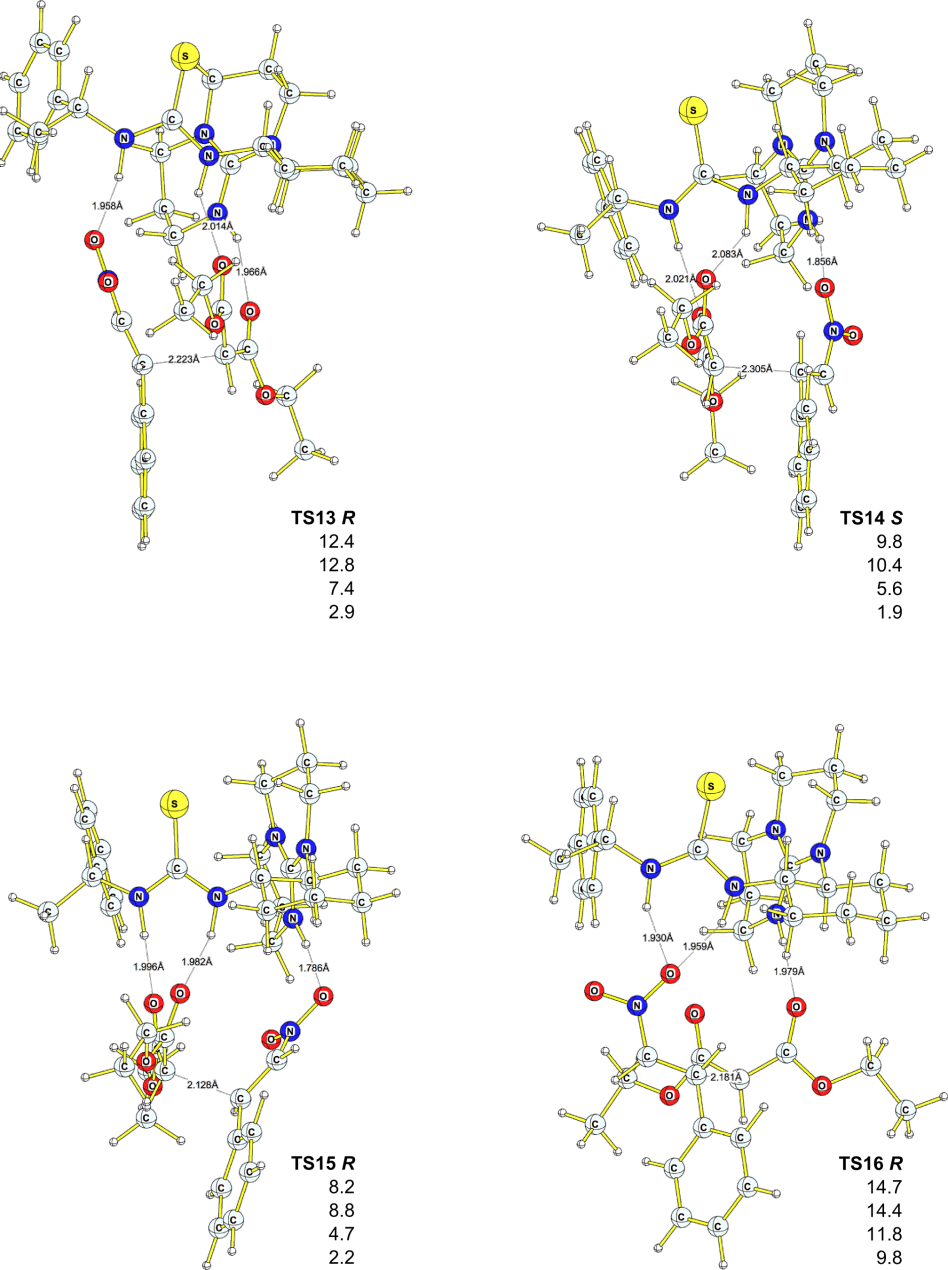
Geometries of transition states for *R* and *S* products with **7-TABD** catalyst. Relative energies (to **Init13**) are in kcal·mol^−1^, B3PW91/6–31G(d) (first entry), DFT-PCM (second entry), MP2/6–31G(d)//B3PW91/6–31G(d) (third entry), DFT-D (fourth entry). Bond lengths are in Å.

For the modified **7-TABD** catalyst, we found only two conformers that differ by ca. 6–8 kcal·mol^−1^ (Figure 5). Assuming that the first part of the reaction pathway (coordination of either malonate or nitrostyrene to the catalyst, followed by addition of the third molecule) proceeds similarly to the pathway described above for the catalyst **7**/nitrostyrene/malonate system (Scheme 5), we focused our attention on the second part of the reaction, the C–C bond formation. We found four transition states, three of which lead to the *R* product and only one to the *S* equivalent (Figure 6).

Compared to the initial complexes **INIT1**–**INIT12**, the energy difference between the initial complexes **INIT13**–**INIT16** is much higher (between 4.6 and 8.0 kcal·mol^−1^, Table 3). Thus, in the case of the **7-TABD** catalyst, interconversion between the initial complexes is less probable. Again, as in the case of catalyst **7**, the activation barriers seem to be overestimated at the gas-phase DFT and DFT-PCM levels, and underestimated with MP2/6–31G(d) and DFT-D corrections. Nevertheless, compared to catalyst **7**, the presence of the cyclic tri-aza moiety in **7-TABD** increases the activation barriers. The reaction is calculated to favour the *R* product, as both the initial complex and the TS leading to the *R* product were found to be lower in energy than the *S* equivalents.

**Table 3:** Computed energy differences between initial and final complexes and activation barriers with respect to the **Init13** complex. Energies are in kcal·mol^−1^, B3PW91/6–31G(d), (DFT-PCM), {MP2/6–31G(d)//B3PW91/6–31G(d)} and [DFT-D].

	INIT	TS	FIN

**13** ***R***	0.0 (0.0) {0.0} [0.0]	12.4 (12.8) {7.4} [6.8]	−2.4 (−2.6) {−13.2} [−8.5]
**14** ***S***	5.8 (4.6) {7.2} [6.9]	9.8 (10.4) {5.6} [5.4]	−3.4 (−7.0) {−9.4} [−3.3]
			
**15** ***R***	7.7 (8.0) {7.2} [6.5]	8.2 (8.8) {4.7} [5.5]	−2.4 (−2.7) {−12.5} [−7.5]
			
**16** ***R***	2.9 (1.6) {5.2} [5.6]	14.7 (14.4) {11.8} [12.7]	−2.2 (−4.2) {−11.4} [−6.6]

## Conclusion

In summary, we have demonstrated that the new guanidine derived chiral thiourea **7** can catalyse the asymmetric nitro-Michael addition of diethyl malonate with trans-β-nitrostyrene, giving high yields (up to 96%) but low enantioselectivities. Based on extensive computational studies, the low observed enantioselectivity of this nitro-Michael reaction can be explained in terms of the high activity and high conformational flexibility of the guanidine-thiourea catalyst, which lead to thermodynamic control of the reaction products. For further experimental studies, a modified more conformationally rigid catalyst is proposed.

We would like to stress here that studies on systems of this size are influenced quite strongly by dispersion contributions [60–66] and that standard DFT calculations should be supplemented by at least single-point MP2 energy corrections with an extensive, large basis set. The DFT-D method agrees very well with the MP2 results.

## Experimental

**General:** Reagents obtained from commercial sources were used without further purification. Dichloromethane was dried by heating under reflux over P_2_O_5_ and by distillation. Diethylether was dried by heating under reflux over sodium/benzophenone and by distillation prior to use. Solvents for chromatography were distilled prior to use. TLC chromatography was performed on precoated aluminium silica gel ALUGRAM SIL G/UV254 plates (Macherey-Nagel GmbH & Co.). Flash chromatography was performed on silica gel 60 Å (Acros particle size: 0.035–0.070 mm). NMR spectra were recorded on a Bruker Avance 300. FAB mass spectra were measured with a Micromass: ZabSpec. The enantiomeric excess of products was determined by chiral HPLC analysis in comparison with authentic racemic material. HPLC measurements were performed using Agilent 1200 Series equipment: vacuum degasser G1322-90010, quaternary pump G1311-90010, thermostated column compartment G1316-90010, diode array and multiple-wavelength detector SL G1315-90012, standard and preparative autosampler G1329-90020, and Agilent Chemstation for LC software.

**(*R*)-(−)-α-Methylbenzyl isothiocyanate (2):** To a solution of (*R*)-1-phenylethylamine (3.000 g, 3.151 mL, 24.76 mmol, 1.0 equiv) in anhydrous diethyl ether (20 mL), cooled to 0 °C and under a nitrogen atmosphere, carbon disulfide (12.065 g, 158.46 mmol, 9.6 mL, 6.4 equiv) and *N*,*N′*-dicyclohexylcarbodiimide (5.109 g, 24.76 mmol, 1.0 equiv) were added. The reaction mixture was stirred overnight and allowed to warm to ambient temperature during this time. The precipitated *N*,*N″*-dicyclohexylthiourea was filtered off, washed three times with diethyl ether and discarded. After evaporation of the filtrate, the residue was subjected to flash chromatography over silica gel (petrol ether/ethyl acetate 8:1) to yield **2** (3.909 g, 23.94 mmol, 97%) as a colourless oil. [α]_D_^25^ −4.3 (*c* 1.0, acetone); ^1^H NMR (300 MHz, CDCl_3_) δ 1.68 (d, *J* = 6.7 Hz, 3H), 4.92 (q, *J* = 6.7 Hz, 1H), 7.27–7.50 (m, 5H) ppm; ^13^C NMR (75 MHz, CDCl_3_) δ 24.96, 57.00, 125.39, 128.18, 128.88, 140.11 ppm.

**Primary amine-thiourea 3:** To a solution of (*S*,*S*)-1,2-diaminocyclohexane (**1**) (1.273 g, 11.15 mmol, 1.0 equiv) in anhydrous dichloromethane (100 mL), at ambient temperature and under a nitrogen atmosphere, a solution of **2** (1.820 g, 11.15 mmol, 1.0 equiv) in anhydrous dichloromethane (60 mL) was added dropwise over 7 h. The solvent was evaporated and flash chromatography on silica gel (first with ethyl acetate to elute impurities, then with ethyl acetate/ethanol 8:1) gave **3** (1.731 g, 6.24 mmol, 56%) as a colourless solid. [α]_D_^25^ −85 (*c* 1.0, chloroform); ^1^H NMR (300 MHz, DMSO-*d*_6_) δ 0.90–1.30 (m, 4H), 1.39 (d, *J* = 7.0 Hz, 3H), 1.49–1.67 (m, 2H), 1.72–1.84 (m, 1H), 1.88–2.02 (m, 1H), 2.40–2.46 (m, 1H), 5.34–5.52 (m, 1H), 7.14–7.42 (m, 5H) ppm; ^13^C NMR (75 MHz, DMSO-*d*_6_) δ 22.81, 24.71, 24.84, 31.82, 34.69, 52.63, 54.53, 59.73, 126.42, 126.95, 128.59, 144.85, 182.03 ppm; MS–FAB (*m*/*z*): 181, 262, 278 [M + H]^+^, 289, 391.

**Compound 5:** A solution of **3** (0.100 g, 0.36 mmol, 1.0 equiv), 1,3-bis(*tert*-butoxycarbonyl)-2-(trifluoromethylsulfonyl)-guanidine (0.141 g, 0.36 mmol, 1.0 equiv) and triethylamine (0.036 g, 0.049 mL, 0.36 mmol, 1.0 equiv) in dichloromethane (5 mL) was stirred for 24 h at ambient temperature and evaporated. The residue was purified by flash chromatography over silica gel (petrol ether/ethyl acetate 6:1) to obtain **5** (0.182 g, 0.35 mmol, 98%) as a colourless solid. ^1^H NMR (300 MHz, DMSO-*d*_6_) δ 1.17–1.36 (m, 4H) 1.41 (s, 3H), 1.45 (s, 9H), 1.47 (s, 9H), 1.51–1.60 (m, 2H), 1.63–1.79 (m, 1H), 1.89–2.07 (m, 1H), 2.60–2.68 (m, 1H), 3.83–3.98 (m, 1H), 4.15–4.32 (m, 1H), 7.16–7.39 (m, 5H), 7.77 (m, 1H), 8.33 (d, *J* = 7.7 Hz, 1H), 9.02 (m, 1H), 11.56 (m, 1H) ppm; MS–FAB (*m*/*z*): 193, 199, 205, 225, 260, 287, 320 [M − 2Boc + H]^+^, 420 [M – Boc + H]^+^, 521 [M + H]^+^.

**Compound 6:** A solution of **5** (0.160 g, 0.31 mmol, 1.0 equiv) in dichloromethane (3 mL) was treated with trifluoroacetic acid (1.535 g, 1.0 mL, 13.46 mmol, 43.4 equiv) at ambient temperature for 7 h. The solvents were evaporated and the residue was purified by column chromatography over silica gel (dichloromethane/methanol 95:5) to yield **6** (0.112 g, 0.26 mmol, 85%) as a colourless, hygroscopic solid. [α]_D_^25^ −13.62 (*c* 0.1, ethanol); ^1^H NMR (300 MHz, DMSO-*d*_6_) δ 1.05–1.35 (m, 4H), 1.41 (d, *J* = 6.2 Hz, 3H), 1.51–1.74 (m, 2H), 1.79–1.93 (m, 1H), 1.93–2.19 (m, 1H), 2.56–2.68 (m, 1H), 3.37–3.47 (m, 1H), 4.11 (d, *J* = 6.2 Hz, 1H), 6.65–6.97 (m, 2H), 7.12–7.58 (m, 9H), 7.91–8.06 (m, 1H) ppm; ^13^C NMR (75 MHz, DMSO-*d*_6_) δ 14.15, 20.72, 22.39, 23.54, 30.92, 31.45, 53.55, 59.72, 115.64, 118.76, 125.52, 126.03, 126.23, 128.33, 144.46, 156.41, 158.44, 170.34 ppm. MS–FAB (*m*/*z*): 107, 120, 124, 136, 154, 199, 286, 289, 307, 320 [M − CF_3_COO^−^]^+^, 376, 391; Anal. calcd: C, 49.87; H, 6.05; N, 16.16; S, 7.40; found: C, 47.41; H, 5.37; N, 13.98; S, 7.26.

**Guanidine-thiourea 7:** Compound **6** (0.115 g, 0.27 mmol, 1.0 equiv) was treated with Amberlyst A26 (OH^−^ form) (1.885 g) in methanol (20 mL) for 15 min. The ion exchanger was filtered off over Celite and the filtrate was evaporated to yield **7** (0.081 g, 0.25 mmol, 93 %) as a white solid. [α]_D_^25^ +19.92 (*c* 0.1, methanol); ^1^H NMR (300 MHz, DMSO-*d*_6_) δ 0.97–1.44 (m, 4H), 1.44–1.58 (m, 3H), 1.58–1.80 (m, 2H), 1.81–1.94 (m, 1H), 1.94–2.37 (m, 1H), 2.48–2.57 (m, 1H), 3.44–3.58 (m, 1H), 3.71–3.98 (m, 1H), 4.60 (br s, 4H), 7.22–7.50 (m, 5H) ppm; MS–FAB (*m*/*z*): 107, 120, 136, 154, 176, 199, 286, 307, 320 [M + H]^+^, 376, 391.

**Henry reaction of 3-phenylpropionaldehyde (8) and nitromethane (9), mediated by 7: 4-Phenyl-1-nitro-2-butanol (10):** A solution of **7** (0.1 equiv), 3-phenylpropionaldehyde (1.0 equiv) and nitromethane (3.0 equiv) in toluene ([aldehyde] = 0.11 M) was stirred for the appropriate time and at the corresponding temperature. The organic phase was diluted with toluene, washed with saturated aqueous ammonium chloride solution, and dried over magnesium sulfate. Product **10** was isolated by preparative thin-layer chromatography over silica gel. ^1^H NMR (300 MHz, DMSO-*d*_6_) δ 1.58–1.83 (m, 2H), 2.55–2.82 (m, 2H), 4.05–4.17 (m, 1H), 4.39 (dd, *J* = 9.3 Hz, *J* = 12.2 Hz, 1H), 4.71 (dd, *J* = 3.0 Hz, *J* = 12.2 Hz, 1H), 5.48 (d, *J* = 6.4 Hz, 1H), 7.10–7.36 (m, 5H) ppm; ^13^C NMR (75 MHz, DMSO-*d*_6_): δ 30.62, 35.36, 67.21, 81.34, 125.56, 128.09, 141.33 ppm; MS–FAB (*m*/*z*): 107, 120, 124, 137, 149 [M − NO_2_^−^]^+^, 154, 167, 289, 307, 391.

**Michael addition of 2,4-pentanedione (12) and *trans*-β-nitrostyrene (11), mediated by 7: 3-(2-Nitro-1-phenylethyl)-pentane-2,4-dione (13):** A solution of **7** (8.99 mg, 28.14 μmol, 0.2 equiv) and acetylacetone (140.87 mg, 1407.00 μmol, 10.0 equiv) was stirred for 5 min at ambient temperature. *trans*-β-Nitrostyrene was added and the mixture was stirred for 5 d at ambient temperature. The solution is diluted with ethyl acetate (10 mL), washed with aqueous 20% potassium hydrogen sulfate solution (3 mL) and brine (3 mL). After drying over magnesium sulfate, the organic phase was evaporated and purified by column chromatography over silica gel (petrol ether/ethyl acetate 2:1) to yield **13** (19.00 mg, 76.23 μmol, 54%). ^1^H NMR (300 MHz, CDCl_3_) δ 1.87 (s, 3H), 2.21 (s, 3H), 4.12–4.23 (m, 1H), 4.30 (d, *J* = 10.7 Hz, 1H), 4.48–4.64 (m, 2H), 7.07–7.33 (m, 5H) ppm; ^13^C NMR (75 MHz, CDCl_3_) δ 29.54, 30.38, 42.74, 70.62, 78.11, 127.90, 128.48, 129.27, 135.97, 200.98, 201.70 ppm.

**Michael addition of diethylmalonate (14) and *trans*-β-nitrostyrene (11), mediated by 7: Diethyl 2-(2-nitro-1-phenylethyl)malonate (15):** A solution of *trans*-β-nitrostyrene (1.0 equiv), diethyl malonate (5.0 equiv) and **7** (0.2 equiv) in the desired solvent ([*trans*-β-nitro-styrene] = 0.1 M) is stirred at a certain temperature and for the appropriate time. The reaction is quenched with concentrated aqueous hydrochloric acid/methanol 1:10, and subjected to flash chromatography over silica gel (petrol ether/ethyl acetate 6:1). ^1^H NMR (300 MHz, CDCl_3_) δ 0.98 (2t, *J* = 7.2 Hz, 3H), 1.20 (2t, *J* = 7.2 Hz, 3H), 3.76 (2d, *J* = 9.4 Hz, 1H), 3.94 (2q, *J* = 7.2 Hz, 2H), 4.10–4.24 (m, 3H), 4.73–4.92 (m, 2H), 7.11–7.33 (m, 5H) ppm; ^13^C NMR (150.8 MHz, CDCl_3_) δ 13.66, 13.90, 42.89, 54.88, 61.82, 62.10, 77.59, 127.96, 128.28, 128.86, 136.14, 166.76, 167.40 ppm.

### Computational methods

Geometries of all structures were fully optimized at the B3PW91 [67–69] level of theory by using the 6–31G(d) [70–80] basis set within the Gaussian 03 program package [81]. Stationary points were confirmed to be minima or transition states by calculating the normal vibrations within the harmonic approximation. The reaction pathways along both directions from the transition structures were followed by the IRC method [82–83]. DFT-computed energies were corrected for zero-point vibrational energies (ZPVE). Single-point self-consistent reaction field (SCRF) [84] calculations were used to calculate the solvation energies in tetrahydrofuran within the PCM model (denoted as DFT-PCM). Single-point MP2 [66] energies with DZ and TZ basis sets were computed at the B3PW91 optimized geometries (denoted as MP2/6–31G(d)//B3PW91/6–31G(d) and MP2/6–311+G(d,p)//B3PW91/6–31G(d), respectively). Single-point DFT calculations with empirical van der Waals corrections [63] were performed with the ORCA program [85].

## Supporting Information

The Supporting Information File features data from DFT computations (computed absolute energies (Hartree) and zero-point vibrational energies (ZPVE, kcal·mol^−1^) at different levels of theory) as well as the respective GAUSSIAN archive entries.

File 1 Detailed information about the DFT calculations

## References

[1] Ma J-A, Cahard D (2004). Angew Chem, Int Ed.

[2] Shibasaki M, Yoshikawa N (2002). Chem Rev.

[3] Gröger H (2001). Chem–Eur J.

[4] Shibasaki M, Sasai H, Arai T (1997). Angew Chem, Int Ed Engl.

[5] Dalko P I, Moisan L (2001). Angew Chem, Int Ed.

[6] Berkessel A, Groeger H (2004). Asymmetric Organocatalysis - From Biomimetic Concepts to Applications in Asymmetric Synthesis.

[7] Pihko P M (2004). Angew Chem, Int Ed.

[8] Seayad J, List B (2005). Org Biomol Chem.

[9] Schreiner P R (2003). Chem Soc Rev.

[10] Takemoto Y (2005). Org Biomol Chem.

[11] Taylor M S, Jacobsen E N (2006). Angew Chem, Int Ed.

[12] Connon S J (2006). Chem–Eur J.

[13] Doyle A G, Jacobsen E N (2007). Chem Rev.

[14] Okino T, Hoashi Y, Takemoto Y (2003). J Am Chem Soc.

[15] Okino T, Nakamura S, Furukawa T, Takemoto Y (2004). Org Lett.

[16] Maher D J, Connon S J (2004). Tetrahedron Lett.

[17] Fuerst D E, Jacobsen E N (2005). J Am Chem Soc.

[18] Yoon T P, Jacobsen E N (2005). Angew Chem, Int Ed.

[19] Li B-J, Jiang L, Liu M, Chen Y-C, Ding L-S, Wu Y (2005). Synlett.

[20] Hoashi Y, Okino T, Takemoto Y (2005). Angew Chem, Int Ed.

[21] Vakulya B, Varga S, Csampai A, Soos T (2005). Org Lett.

[22] Okino T, Hoashi Y, Furukawa T, Xu X, Takemoto Y (2005). J Am Chem Soc.

[23] McCooey S H, Connon S J (2005). Angew Chem, Int Ed.

[24] Berkessel A, Cleemann F, Mukherjee S, Müller T N, Lex J (2005). Angew Chem, Int Ed.

[25] Berkessel A, Cleemann F, Mukherjee S (2005). Angew Chem, Int Ed.

[26] Berkessel A, Mukherjee S, Cleemann F, Müller T N, Lex J (2005). Chem Commun.

[27] Wang J, Li H, Yu X, Zu L, Wang W (2005). Org Lett.

[28] Inokuma T, Hoashi Y, Takemoto Y (2006). J Am Chem Soc.

[29] Xu X, Furukawa T, Okino T, Miyabe H, Takemoto Y (2006). Chem–Eur J.

[30] Marcelli T, van der Haas R N S, van Maarseveen J H, Hiemstra H (2006). Angew Chem, Int Ed.

[31] Cao Y-J, Lu H-H, Lai Y-Y, Lu L-Q, Xiao W-J (2006). Synthesis.

[32] Cao C-L, Ye M-C, Sun X-L, Tang Y (2006). Org Lett.

[33] Zuend S J, Jacobsen E N (2007). J Am Chem Soc.

[34] Wang B, Wu F, Wang Y, Liu X, Deng L (2007). J Am Chem Soc.

[35] Dinér P, Nielsen M, Bertelsen S, Niess B, Jørgensen K A (2007). Chem Commun.

[36] Tsogoeva S B, Wei S (2006). Chem Commun.

[37] Huang H, Jacobsen E N (2006). J Am Chem Soc.

[38] Yalalov D A, Tsogoeva S B, Schmatz S (2006). Adv Synth Catal.

[39] Lalonde M P, Chen Y, Jacobsen E N (2006). Angew Chem, Int Ed.

[40] Wei S, Yalalov D A, Tsogoeva S B, Schmatz S (2007). Catal Today.

[41] Liu K, Cui H-F, Nie J, Dong K-Y, Li X-J, Ma J-A (2007). Org Lett.

[42] Yalalov D A, Tsogoeva S B, Shubina T E, Martynova I M, Clark T (2008). Angew Chem, Int Ed.

[43] Tsogoeva S B, Hateley M J, Yalalov D A, Meindl K, Weckbecker C, Huthmacher K (2005). Bioorg Med Chem.

[44] Tsogoeva S B, Yalalov D A, Hateley M J, Weckbecker C, Huthmacher K (2005). Eur J Org Chem.

[45] Berner O M, Tedeschi L, Enders D (2002). Eur J Org Chem.

[46] Tsogoeva S B (2007). Eur J Org Chem.

[47] Almasi D, Alonso D A, Nájera C (2007). Tetrahedron: Asymmetry.

[48] Armarego W L F, Chai C L L (2003). Purification of Laboratory Chemicals.

[49] Ishikawa T, Kumamoto T (2006). Synthesis.

[50] Sohtome Y, Hashimoto Y, Nagasawa K (2005). Adv Synth Catal.

[51] Sohtome Y, Takemura N, Iguchi T, Hashimoto Y, Nagasawa K (2006). Synlett.

[52] Sohtome Y, Hashimoto Y, Nagasawa K (2006). Eur J Org Chem.

[53] Sohtome Y, Takemura N, Takada K, Takagi R, Iguchi T, Nagasawa K (2007). Chem–Asian J.

[54] Feichtinger K, Zapf C, Sings H L, Goodman M (1998). J Org Chem.

[55] Dodd D S, Wallace O B (1998). Tetrahedron Lett.

[56] Ley S V, Massi A (2000). J Chem Soc, Perkin Trans 1.

[57] Hamza A, Schubert G, Soos T, Pápai I (2006). J Am Chem Soc.

[58] (2005). VAMP.

[59] Schenker S, Schneider C, Tsogoeva S B, Clark T (2011). J Chem Theory Comput.

[60] He X, Fusti-Molnar L, Cui G, Merz K M (2009). J Phys Chem B.

[61] Schreiner P R, Chernish L V, Gunchenko P A, Tikhonchuk E Y, Hausmann H, Serafin M, Schlecht S, Dahl J E P, Carlson R M K, Fokin A A (2011). Nature.

[62] Grimme S, Schreiner P R (2011). Angew Chem, Int Ed.

[63] Grimme S (2006). J Comput Chem.

[64] Sieffert N, Bühl M (2009). Inorg Chem.

[65] Seebach D, Grošelj U, Schweizer W B, Grimme S, Mück-Lichtenfeld C (2010). Helv Chim Acta.

[66] Møller C, Plesset M S (1934). Phys Rev.

[67] Becke A D (1993). J Chem Phys.

[68] Perdew J P, Burke K, Wang Y (1996). Phys Rev B.

[69] Perdew J P, Chevary J A, Vosko S H, Jackson K A, Pederson M R, Singh D J, Fiolhais C (1992). Phys Rev B.

[70] Ditchfield R, Hehre W J, Pople J A (1971). J Chem Phys.

[71] Hariharan P C, Pople J A (1973). Theor Chim Acta.

[72] Hariharan P C, Pople J A (1974). Mol Phys.

[73] Hehre W J, Ditchfield R, Pople J A (1972). J Chem Phys.

[74] Gordon M S (1980). Chem Phys Lett.

[75] Blaudeau J-P, McGrath M P, Curtiss L A, Radom L (1997). J Chem Phys.

[76] Francl M M, Pietro W J, Hehre W J, Binkley J S, Gordon M S, DeFrees D J, Pople J A (1982). J Chem Phys.

[77] Binning R C, Curtiss L A (1990). J Comput Chem.

[78] Rassolov V A, Pople J A, Ratner M A, Windus T L (1998). J Chem Phys.

[79] Rassolov V A, Ratner M A, Pople J A, Redfern P C, Curtiss L A (2001). J Comput Chem.

[80] Frisch M J, Pople J A, Binkley J S (1984). J Chem Phys.

[81] (2004). Gaussian 03.

[82] Gonzalez C, Schlegel H B (1990). J Phys Chem.

[83] Gonzalez C, Schlegel H B (1989). J Chem Phys.

[84] Foresman J B, Keith T A, Wiberg K B, Snoonian J, Frisch M J (1996). J Phys Chem.

[85] (2012). Orca, an ab initio, DFT and semiempirical SCF-MO package.

