# Supplementary material for: Synthesis and evaluation of new guanidine-thiourea organocatalyst for the nitro-Michael reaction: Theoretical studies on mechanism and enantioselectivity
Source: Beilstein J Org Chem. 2012 Sep 7;8:1485–98. doi: 10.3762/bjoc.8.168 (PMC3458773; doi:10.3762/bjoc.8.168)
Supplement: File 1 — Detailed information about the DFT calculations [file Beilstein_J_Org_Chem-08-1485-s001.pdf]

Supporting Information

for

Synthesis and evaluation of new guanidine-thiourea organocatalyst for the nitro-Michael reaction: Theoretical studies on mechanism and enantioselectivity

Tatyana E. Shubina*[1], Matthias Freund[2], Sebastian Schenker[2], Timothy Clark*[1] and Svetlana B. Tsogoeva*[2]

Address: [1]Computer Chemistry Center and Interdisciplinary Center for Molecular Materials, University of Erlangen-Nuremberg, Nägelsbachstr. 25, 91052 Erlangen, Germany and [2]Department of Chemistry and Pharmacy, Chair of Organic Chemistry I, University of Erlangen-Nuremberg, Henkestraße 42, 91054, Erlangen, Germany

E-mail: Tatyana E. Shubina - tatyana.shubina@chemie.uni-erlangen.de; Timothy Clark - clark@chemie.uni-erlangen.de; Svetlana B. Tsogoeva - tsogoeva@chemie.uni-erlangen.de

*Corresponding author

Detailed information about the DFT calculations

Calculations of the enantiomeric excess ee

For calculation of ee the theory of the activated complex was used. Based on this theory, the kinetic constant *k* is a function of the free activation energy (Equation 1) [1].

$$k = \frac{k_B \cdot T}{h} e^{-\frac{\Delta G^{\neq}}{RT}} \qquad (1)$$

The enantiomeric excess ee is defined as the ratio of the difference of the product concentrations to their sum. If it is assumed that the transition states are totally converted into the products, the product concentrations can be replaced by the transition state (TS) concentrations in the expression for ee. The TS concentrations are the product of the reactant concentrations and the kinetic constants. This results in the following expression (Equation 2):

$$\mathrm{ee} = \frac{[\mathrm{TS}_R] - [\mathrm{TS}s]}{[\mathrm{TS}_R] + [\mathrm{TS}s]} = \frac{k_R[\mathrm{C_R}] - k_S[\mathrm{C_S}]}{k_R[\mathrm{C_R}] + k_S[\mathrm{C_S}]} \qquad (2)$$

Since the starting point is the same for the *R* and *S* pathways, the reactant concentration is the same ($[C_R]=[C_S]$) and Equation 2 can be simplified to Equation 3.

$$\mathrm{ee} = \frac{k_R - k_S}{k_R + k_S} = \frac{1 - k_S / k_R}{1 + k_S / k_R} \qquad (3)$$

$$\frac{k_S}{k_R} = e^{-\frac{\Delta G_S^{\neq} - \Delta G_R^{\neq}}{RT}} = e^{-\frac{\Delta G_{RS}}{RT}} \qquad (4)$$

$$\mathrm{ee} = \frac{1 - e^{-\frac{\Delta G_{RS}}{RT}}}{1 + e^{-\frac{\Delta G_{RS}}{RT}}} \qquad (5)$$

Thus, ee can be expressed simply as a function of the free-energy difference between the two TS.

Table S1: Computed absolute energies (Hartree) and zero-point vibrational energies (ZPVE, kcal·mol^{-1}) at different levels of theory.

	B3PW91/6–31G(d)		MP2/6–311++G(d,p)//B3PW91/6–31G(d)
	E, Hartree	*ZPE*, kcal·mol^{-1}	*E*
**7a**	-1296.36288	257.4	-1293.82784
**7b**	-1296.35890	256.7	-1293.81598
**7c**	-1296.35385	257.1	-1293.81306
**7d**	-1296.34879	257.0	-1293.80697
**14**	-574.67641	120.2	-573.511336
**11**	-513.95497	86.3	-512.868865
**Complex1**	-1871.05130	378.6	-1867.36309
**TS1 abst**	-1871.04057	376.3	-1867.34815
**Complex2**	-1871.05200	379.1	-1867.36063
**Complex2a**	-1871.02988	378.0	-1867.32660
**CatN1**	-1810.33177	344.3	-1806.72577
**CatN2**	-1810.31678	343.8	-1806.69915
**CatN3**	-1810.31548	343.9	-1806.68976
**CatN4**	-1810.31588	344.2	-1806.69807
**CatN5**	-1810.31522	344.2	-1806.70782
**Init1**	-2385.01243	466.1	-2380.25480
**TS1**	-2385.01044	466.4	-2380.25619
**Fin1**	-2385.02907	467.5	-2380.28565
**Init2**	-2385.01606	466.2	-2380.25641
**TS2**	-2385.00825	466.6	-2380.25465
**Fin2**	-2385.02826	468.3	-2380.28306
**Init3**	-2385.01807	465.9	-2380.25365
**TS3**	-2385.01461	466.7	-2380.25991
**Fin3**	-2385.02947	467.9	-2380.28666
**Init5**	-2385.01579	466.4	-2380.25323
**TS5**	-2385.01391	466.6	-2380.25725
**Fin5**	-2385.03207	467.5	-2380.28914
**Init6**	-2385.01739	466.0	-2380.25536

**TS6**	-2385.01400	466.6	-2380.25929
**Fin6**	-2385.02935	467.9	-2380.28825
**Init7**	-2385.02332	466.5	-2380.26253
**TS7**	-2385.00833	466.9	-2380.26401
**Fin7**	-2385.02404	468.1	-2380.28770
**Init8**	-2385.02833	466.4	-2380.26937
**TS8**	-2385.01900	467.0	-2380.27124
**Fin8**	-2385.04179	468.1	-2380.30268
**Init9**	-2385.02758	466.0	-2380.26793
**TS9**	-2385.01711	466.4	-2380.26405
**Fin9**	-2385.04204	468.0	-2380.30140
**Init10**	-2385.03004	466.4	-2380.26876
**TS10**	-2385.01786	466.9	-2380.27004
**Fin10**	-2385.04011	468.2	-2380.29130
**Init11**	-2385.02891	466.5	-2380.26811
**TS11**	-2385.01568	466.4	-2380.26712
**FIN11**	-2385.03455	467.8	-2380.29556
**Init12**	-2,385.0275	466.2	-2380.26903
**TS12**	-2385.01904	466.8	-2380.27386
**Fin12**	-2385.04204	468.0	-2380.30144
	B3PW91/6–31G(d)		MP2/6–31G(d)// B3PW91/6–31G(d)
	E, Hartree	ZPE, kcal mol^{-1}	E, Hartree
**3-TABDa**	-1529.74038	338.9	-1526.59967
**3-TABDb**	-1529.72748	338.5	-1526.58731
**Init13**	-2618.39687	548.7	-2611.69081
**TS13**	-2618.37707	548.7	-2611.67899
**Fin13**	-2618.40341	550.4	-2611.71183
**Init14**	-2618.38723	548.5	-2611.67932
**TS14**	-2618.38103	548.6	-2611.68187
**Fin14**	-2618.40422	549.9	-2611.70584
**Init15**	-2618.38441	548.6	-2611.67939
**TS15**	-2618.38408	548.9	-2611.68330
**Fin15**	-2618.40307	550.2	-2611.71068
**Init16**	-2618.39143	548.2	-2611.68258
**TS16**	-2618.37362	548.8	-2611.67205
**Fin16**	-2618.40280	550.2	-2611.70900

References

[1] Benson, S.W. *Thermochemical Kinetics,* 2nd ed.; John Wiley & Sons: New York, 1976; p 94.

Gaussian Archive Entries:

Catalyst 7

7a

1\1\FAU-CCC-ALTIX2\SP\RMP2-FC\6-31G(d)\C16H25N5S1\SHUBINA\17-Mar-2008\
0\\#P MP2/6-31G(D) SCF=TIGHT NAME=SHUBINA\\catalyst01\\0,1\S,0,0.18473
,1.913306,-2.300628\N,0,-0.913453,-0.36176,-1.36578\N,0,1.327745,-0.44
9093,-1.678304\N,0,-0.100623,-1.753507,1.014044\N,0,0.799901,-0.162644
,2.527567\N,0,-1.182663,0.270481,1.406763\C,0,-2.130887,0.283486,-0.90
416\C,0,-2.339352,-0.06018,0.58703\C,0,-3.598139,0.629011,1.122061\C,0
,-4.829798,0.258729,0.29031\C,0,-4.621071,0.560971,-1.195667\C,0,-3.35
6555,-0.115299,-1.730677\C,0,0.199961,0.316213,-1.750759\C,0,2.689975,
0.041854,-1.861274\C,0,3.479431,-0.924268,-2.743255\C,0,3.329198,0.319
501,-0.505288\C,0,3.279988,1.612814,0.030268\C,0,3.922523,-0.694824,0.
25659\C,0,3.810757,1.885317,1.290144\C,0,4.45096,-0.426658,1.520277\C,
0,4.39753,0.866787,2.041542\C,0,-0.136873,-0.598716,1.606054\H,0,-3.73
9751,0.353921,2.174015\H,0,-2.461291,-1.146863,0.679126\H,0,-1.974756,
1.365276,-1.009029\H,0,-5.037616,-0.813849,0.41708\H,0,-5.709372,0.792
759,0.670852\H,0,-5.495375,0.238838,-1.774645\H,0,-4.53709,1.648261,-1
.338052\H,0,-3.47446,-1.208695,-1.699425\H,0,-3.177316,0.155927,-2.777
021\H,0,-3.452547,1.72017,1.094242\H,0,-0.721421,-1.2297,-0.855576\H,0
,1.241952,-1.27846,-1.097122\H,0,2.580944,0.994746,-2.387267\H,0,4.509
206,-0.574518,-2.872099\H,0,3.516945,-1.935938,-2.321825\H,0,3.005641,
-0.993024,-3.727016\H,0,2.809962,2.403683,-0.548971\H,0,3.987491,-1.70
6034,-0.138874\H,0,3.773254,2.898845,1.682696\H,0,4.91825,-1.225908,2.
090983\H,0,4.82005,1.079724,3.020425\H,0,-0.962128,1.256196,1.465671\H
,0,0.702581,0.782911,2.871766\H,0,1.760445,-0.401168,2.313959\H,0,0.62
2747,-2.336127,1.433423\\Version=IA64L-G03RevC.02\State=1-A\HF=-1290.2
316019\MP2=-1293.2365847\RMSD=9.679e-09\PG=C01 [X(C16H25N5S1)]\\@

7b

1\1\FAU-CCC-ALTIX2\SP\RMP2-FC\6-31G(d)\C16H25N5S1\SHUBINA\17-Mar-2008\
0\\#P MP2/6-31G(D) SCF=TIGHT NAME=SHUBINA\\catalyst01-minimumconf\\0,1
\S,0,-1.430386,2.547568,-0.113303\N,0,-0.686452,0.015252,0.427643\N,0,
0.908535,1.398463,-0.520342\N,0,0.859119,-1.821768,2.083379\N,0,0.8730
37,-1.180379,4.372813\N,0,-1.070016,-1.069772,3.115398\C,0,-2.017554,-
0.309066,0.921559\C,0,-1.930819,-1.411467,1.991469\C,0,-3.331026,-1.75
9976,2.511177\C,0,-4.28085,-2.163313,1.381552\C,0,-4.348084,-1.082721,
0.30158\C,0,-2.952289,-0.739044,-0.217635\C,0,-0.362174,1.233048,-0.05
8603\C,0,1.901359,0.368122,-0.83752\C,0,2.994544,0.296681,0.234083\C,0
,2.455521,0.616012,-2.231338\C,0,2.102379,-0.223724,-3.291584\C,0,3.30
6709,1.697171,-2.490453\C,0,2.587373,0.005443,-4.577786\C,0,3.787896,1
.933538,-3.776225\C,0,3.431549,1.086298,-4.824261\C,0,0.268623,-1.3572
08,3.138069\H,0,-3.241454,-2.562153,3.253413\H,0,-1.481354,-2.304713,1
.540528\H,0,-2.428291,0.603835,1.37722\H,0,-3.928912,-3.104439,0.93430
9\H,0,-5.278215,-2.366965,1.791121\H,0,-4.98862,-1.410201,-0.526613\H,
0,-4.814083,-0.177117,0.716155\H,0,-2.513377,-1.611727,-0.723496\H,0,-
2.995832,0.074477,-0.949108\H,0,-3.7454,-0.884855,3.036428\H,0,0.0495,
-0.647439,0.696791\H,0,1.056963,2.31438,-0.922025\H,0,1.376361,-0.5930
68,-0.858633\H,0,3.72914,-0.476841,-0.01681\H,0,3.521052,1.25231,0.328
518\H,0,2.545449,0.054418,1.201556\H,0,1.437614,-1.065243,-3.108134\H,
0,3.601067,2.365068,-1.683429\H,0,2.302273,-0.660592,-5.388309\H,0,4.4
45255,2.780016,-3.958605\H,0,3.809285,1.26839,-5.827057\H,0,-1.400501,
-0.332417,3.722631\H,0,0.328563,-0.764267,5.115153\H,0,1.837898,-0.884
602,4.356328\H,0,1.793289,-2.158695,2.308178\\Version=IA64L-G03RevC.02
\State=1-A\HF=-1290.229047\MP2=-1293.2281158\RMSD=7.586e-09\PG=C01 [X(
C16H25N5S1)]\\@

7c

1\1\FAU-CCC-ALTIX2\SP\RMP2-FC\6-31G(d)\C16H25N5S1\SHUBINA\17-Mar-2008\
0\\#P MP2/6-31G(D) SCF=TIGHT NAME=SHUBINA\\SSR-MF30//HF/3-21G\\0,1\S,0
,-3.536599,2.663017,3.097267\N,0,-3.389838,1.170495,0.872945\N,0,-2.08

0978,3.032172,0.876637\N,0,-0.125499,-0.67431,1.036154\N,0,-1.546066,-
0.556363,-0.901063\N,0,-2.264619,-1.524381,1.110374\C,0,-4.171457,0.09
0691,1.450577\C,0,-3.684767,-1.26419,0.893672\C,0,-4.502172,-2.408563,
1.50407\C,0,-6.005379,-2.224951,1.286483\C,0,-6.483188,-0.871234,1.814
943\C,0,-5.67457,0.273494,1.204426\C,0,-2.977214,2.270315,1.556438\C,0
,-1.434718,4.239777,1.380777\C,0,-2.188918,5.489857,0.93156\C,0,0.0421
4,4.192714,1.011849\C,0,0.7897,3.055453,1.354647\C,0,0.691822,5.24402,
0.359774\C,0,2.146703,2.978062,1.056335\C,0,2.052792,5.166424,0.05833\
C,0,2.785035,4.034883,0.405008\C,0,-1.233796,-0.901456,0.433291\H,0,-4
.152985,-3.356557,1.07827\H,0,-3.866991,-1.269465,-0.19063\H,0,-3.9920
89,0.111049,2.534084\H,0,-6.227172,-2.295183,0.211059\H,0,-6.551286,-3
.044543,1.769776\H,0,-7.550829,-0.734032,1.604167\H,0,-6.375979,-0.846
881,2.90886\H,0,-5.85508,0.324852,0.119988\H,0,-5.974405,1.238025,1.62
7581\H,0,-4.292574,-2.451278,2.583618\H,0,-2.920186,0.95162,-0.002399\
H,0,-1.762289,2.703298,-0.025972\H,0,-1.521981,4.171969,2.472731\H,0,-
1.758274,6.392517,1.378472\H,0,-2.17796,5.601561,-0.159132\H,0,-3.2281
62,5.410917,1.262747\H,0,0.300413,2.223073,1.855855\H,0,0.139898,6.137
447,0.083131\H,0,2.709458,2.092275,1.341438\H,0,2.538181,5.996621,-0.4
49142\H,0,3.845651,3.976145,0.173522\H,0,-2.006704,-1.641997,2.084448\
H,0,-2.078475,-1.271119,-1.388503\H,0,-0.715157,-0.324671,-1.433496\H,
0,0.582791,-0.325622,0.390866\\Version=IA64L-G03RevC.02\State=1-A\HF=-
1290.2245545\MP2=-1293.2249167\RMSD=8.640e-09\PG=C01 [X(C16H25N5S1)]\\

7d

1\1\FAU-CCC-ALTIX2\SP\RMP2-FC\6-31G(d)\C16H25N5S1\SHUBINA\17-Mar-2008\
0\\#P MP2/6-31G(D) SCF=TIGHT NAME=SHUBINA\\SSR-M30/B3PW91//6-31G(d)\\0
,1\S,0,-1.312537,3.933885,-1.327591\N,0,-0.795147,1.293619,-1.306358\N
,0,0.697005,2.566358,-2.459543\N,0,-0.452104,0.984542,2.402484\N,0,1.5
15087,-0.368452,2.138277\N,0,-0.254944,-0.589033,0.685062\C,0,-1.87410
2,0.993477,-0.368896\C,0,-1.630181,-0.416742,0.200508\C,0,-2.726359,-0
.796002,1.205214\C,0,-4.112906,-0.716672,0.561182\C,0,-4.357597,0.6615
63,-0.05592\C,0,-3.250058,1.029787,-1.044551\C,0,-0.44643,2.546025,-1.
710825\C,0,1.216699,3.740975,-3.155433\C,0,0.53873,3.92607,-4.512154\C
,0,2.735962,3.651254,-3.189572\C,0,3.434464,3.57441,-1.975991\C,0,3.46
9537,3.651403,-4.378237\C,0,4.823092,3.501842,-1.95241\C,0,4.863866,3.
577045,-4.358205\C,0,5.545177,3.501619,-3.147394\C,0,0.23569,0.08977,1
.799724\H,0,-2.533636,-1.811683,1.576898\H,0,-1.697848,-1.112309,-0.65
1527\H,0,-1.847511,1.720862,0.45055\H,0,-4.198183,-1.484196,-0.223466\
H,0,-4.882906,-0.948927,1.307708\H,0,-5.332071,0.687955,-0.559242\H,0,
-4.395831,1.417378,0.74115\H,0,-3.24874,0.324863,-1.889571\H,0,-3.4114
15,2.030571,-1.455555\H,0,-2.659122,-0.11902,2.063003\H,0,-0.036478,0.
619628,-1.255372\H,0,1.026795,1.677363,-2.816782\H,0,0.932042,4.590464
,-2.52275\H,0,0.889695,4.838049,-5.007418\H,0,0.724185,3.075983,-5.180
01\H,0,-0.53989,4.016682,-4.356368\H,0,2.87244,3.575464,-1.044665\H,0,
2.957832,3.714999,-5.334092\H,0,5.345951,3.451773,-1.000169\H,0,5.4151
5,3.579533,-5.295405\H,0,6.630788,3.446775,-3.131565\H,0,0.041788,-1.5
58109,0.677695\H,0,2.122745,-0.536315,1.344564\H,0,1.975168,0.191176,2
.84441\H,0,0.104537,1.460782,3.111799\\Version=IA64L-G03RevC.02\State=
1-A\HF=-1290.2193781\MP2=-1293.2186229\RMSD=7.685e-09\PG=C01 [X(C16H25N5S1)]\\@

14

1\1\FAU-CCC-CCDA091\Freq\RB3PW91\6-31G(d)\C7H12O4\SCHENKER\17-Oct-2007
\0\\#P Geom=AllCheck Guess=Read SCRF=Check GenChk RB3PW91/6-31G(d) Fre
q\\malonicester //B3PW91/6-31G(d)\\0,1\C,1.4583619263,0.1501954286,-0.
517789549\C,0.0455533893,0.233031336,0.0180276572\H,1.5155200098,0.566
4278046,-1.5287783704\H,1.7537365263,-0.9020014036,-0.5665911615\C,2.4
37012906,0.8924189646,0.3746605399\O,-0.5331094769,-0.6682846273,0.577
6614886\O,-0.4809314885,1.4433956668,-0.2206543467\O,3.652189341,0.920
3723002,-0.1965813625\O,2.1774281682,1.3758229386,1.4513508957\C,-1.80
17515907,1.6620143648,0.3110958875\C,-2.2019723204,3.079981475,-0.0307
090801\H,-1.7745780599,1.490711017,1.3919620142\H,-2.4835442183,0.9240
132822,-0.1248923552\H,-3.203504116,3.2873428102,0.3611450116\H,-1.504
0405758,3.7988396352,0.4096877408\H,-2.2186020281,3.2332861247,-1.1145
555179\C,4.686532821,1.5688394998,0.5713938281\C,5.9655829825,1.493014
6786,-0.2322397374\H,4.7757315348,1.0624835906,1.5381871048\H,4.382554
2611,2.6023892786,0.7669432016\H,6.7787629092,1.9765965026,0.319435470

8\H,6.2492506146,0.4528140941,-0.4208296291\H,5.8542184844,2.000682237
9,-1.1955527311\\Version=IA32L-G03RevD.02\State=1-A\HF=-574.6764089\RM
SD=3.175e-09\RMSF=1.276e-06\ZeroPoint=0.1915746\Thermal=0.2044008\ZPE=
120.2148522\Dipole=0.5839846,0.6095285,-0.7391198\DipoleDeriv=-0.40536
0763073,0.0305066,0.0761377,-0.084751\Polar=104.5490099,-1.1279425,78.
4755916,-3.6779024,0.9711052,71.2920057\PG=C01 [X(C7H12O4)]\NImag=0\\0
0.00059445,-0.00346183,0.00665313,0.02644117,-0.12162385,0.27885754\\0
0114,0.00000164\\\@

11

1\1\FAU-CCC-CCDO112\Freq\RB3PW91\6-31G(d)\C8H7N1O2\SHUBINA\13-Mar-2008
\0\\#P Geom=AllCheck Guess=Read SCRF=Check GenChk RB3PW91/6-31G(d) Fre
q\\nitrostyrene\\0,1\C,0.4825283049,0.4402521276,0.0893156526\C,-0.946
2372307,0.1618059984,0.0301131328\C,-1.4735727479,-1.1196896909,-0.211
9687889\C,-2.846114489,-1.3212146253,-0.2565826722\C,-3.7218773415,-0.
2502241583,-0.0609206528\C,-1.8398576673,1.2281622195,0.2248857887\C,-
3.2152172196,1.0252778891,0.1800072517\H,-0.8058076323,-1.9625154522,-
0.3660634159\H,-3.2383848524,-2.3169662801,-0.4446488059\H,-4.79579990
93,-0.4127059835,-0.0967717142\H,-1.4443326091,2.2236041101,0.41294494
96\H,-3.8908295608,1.8623542253,0.333069333\H,0.7769730799,1.470186162
4,0.2827373426\C,1.4845521001,-0.434711449,-0.0692128655\N,2.86027698,
0.0035644666,0.0200856992\H,1.4129173737,-1.4952617849,-0.2674932762\O
,3.1022039288,1.1865811315,0.2430215591\O,3.7089144924,-0.8711249063,-
0.1391875178\\Version=AM64L-G03RevD.02\State=1-A\HF=-513.954973\RMSD=1
.806e-09\RMSF=6.033e-05\ZeroPoint=0.1375321\Thermal=0.146658\ZPE=86.30
27094\Dipole=-2.2805758,-0.2199882,-0.0531002\DipoleDeriv=1.131409,0.0
2642637,-0.6952789,-0.0771496,0.0447473,-0.0765608,-0.2903388\Polar=18
0.4451701,-6.3709142,99.3184357,-0.4435557,12.2998334,35.6417995\PG=C0
1 [X(C8H7N1O2)]\NImag=0\\0.73089363,-0.10312621,0.72751110,-0.01632792
897,-0.05066345,0.06943599,0.04006592\\-0.00002345,-0.00007176,0.00003
13769,0.00009362,0.00002221\\\@

Complex1

1\1\GINC-LX64I161\Freq\RB3PW91\6-31G(d)\C23H37N5O4S1\SCHENKER\27-Jan-2
008\0\\#P Geom=AllCheck Guess=Read SCRF=Check GenChk RB3PW91/6-31G(d)
Freq\\deprotDFT-init.com\\0,1\S,-1.2771312627,-3.3542422293,0.78910574
22\N,-1.8861225442,-0.7333522049,0.8596602629\N,0.1988082757,-1.273110
3305,1.6032398814\N,-1.2381523943,1.1377529307,-2.6570776761\N,0.04596
55237,-0.7722786199,-2.4908783576\N,-2.2127923545,-0.9352759605,-1.977
7967324\C,-3.2040322229,-0.9361161559,0.2895281721\C,-3.2889422399,-0.
4054850688,-1.1570763397\C,-4.676368751,-0.6891108958,-1.7443534074\C,
-5.7845808028,-0.0874573749,-0.8768065784\C,-5.6891443156,-0.575066972
4,0.5704141479\C,-4.3005050397,-0.3151756398,1.1621247363\C,-0.9743409
706,-1.7177488624,1.0923287459\C,1.3095501815,-2.1264434592,1.99921382
89\C,2.0752692437,-1.4298167811,3.1295810234\C,2.2421563075,-2.4921306
833,0.8541988257\C,2.5870414226,-1.5597428001,-0.1277910748\C,2.822233
0087,-3.7623711859,0.8081872368\C,3.4928943997,-1.8890829482,-1.135877
5597\C,3.7318286772,-4.0937953313,-0.1942167558\C,4.0709398925,-3.1573
535965,-1.1697376611\C,-1.1578930524,-0.1227349663,-2.3638710752\H,-4.
7180497162,-0.2947296979,-2.7665458823\H,-3.1494878969,0.6841513036,-1
.1194858398\H,-3.343734093,-2.0235581072,0.2688217785\H,-5.7045900668,
1.0100891759,-0.8958461481\H,-6.7676883712,-0.3327679859,-1.2970926954
\H,-6.4558999998,-0.092406044,1.1891818066\H,-5.8945079072,-1.65451324
68,0.6044569303\H,-4.1329975403,0.7708594333,1.24399877\H,-4.225805623
9,-0.7259798402,2.1757749471\H,-4.8122405665,-1.7772897888,-1.82394387
43\H,-1.5658991557,0.2239261179,0.9635576349\H,0.3573607911,-0.2701907
635,1.617586972\H,0.8660430502,-3.0508314002,2.3821373554\H,2.90391750
42,-2.0599524228,3.4661110923\H,2.5014230577,-0.4784689372,2.787984430
3\H,1.4150755323,-1.2329944123,3.9815303703\H,2.1330214585,-0.57224186
21,-0.1229541931\H,2.546811566,-4.5037130365,1.5556807655\H,3.73732629
81,-1.1479522144,-1.8919404509\H,4.1661908557,-5.0903182951,-0.2200342
157\H,4.7742448823,-3.4177932279,-1.9567919535\H,-1.9580242264,-1.8935
162148,-1.7584266241\H,0.2540052411,-1.5036820409,-1.8220239523\H,0.82
53340607,-0.144226494,-2.6536622989\O,2.4614656334,1.3435656486,-1.870
2950203\C,2.1935657687,2.3707288518,-1.2837941424\C,0.8046730058,2.949
3947425,-1.1280411461\O,0.2929362983,1.7205226624,0.8981722626\C,0.259

6072232,2.7620794344,0.2635328473\O,-0.3235797263,3.8746647455,0.71748
85164\C,-0.9302748195,3.8002435453,2.0239686789\C,-1.4299104932,5.1844
687912,2.3718664471\O,3.1048585319,3.1674668579,-0.7022397421\C,4.4896
743428,2.7664454825,-0.7906987193\C,4.8938992102,1.9235386115,0.402595
9745\H,5.9683368513,1.7128151496,0.3580928333\H,4.687121769,2.45063022
32,1.3396250738\H,4.3620764905,0.9679987254,0.4073631087\H,4.640355038
2,2.229830896,-1.7306322705\H,5.041818516,3.7093803976,-0.819576913\H,
-0.1835733135,3.4374420855,2.7372521241\H,-1.7447256317,3.0681222995,1
.9919953208\H,-1.9024443341,5.1696661533,3.3596034953\H,-0.6046114384,
5.9026493844,2.3956820614\H,-2.1681584185,5.5301643976,1.6417523253\H,
0.819035554,4.013999524,-1.3720711378\H,-2.2107792902,1.4338613764,-2.
7175407967\H,0.1200841919,2.4064149046,-1.8146852074\\Version=IA64L-G0
3RevD.01\State=1-A\HF=-1871.0513047\RMSD=3.603e-09\RMSF=8.258e-06\Zero
Point=0.6033383\Thermal=0.6395027\Dipole=0.2355631,2.435849,1.4779559\
2,0.1417466,0.2100218,0.1840004,0.2072439\Polar=337.3905057,-22.689679
7,316.9533931,-16.6448138,-1.7466225,251.8571024\PG=C01 [X(C23H37N5O4S
1)]\NImag=0\\0.06550803,0.03931577,0.29857759,0.02368001,0.04524595,0.
42,0.00131590,0.00179152,-0.00000169,0.08900402,0.07177990,0.15412984\
\0.00000076,0.00000073,0.00000374,0.00000072,0.00000008,0.00000134,0.0
001207,0.00000129\\\@

TS H-abs

1\1\FAU-RRZE-ALTIX-BATCH\Freq\RB3PW91\6-31G(d)\C23H37N5O4S1\SCHENKER\2
0-Jan-2008\0\\#P B3PW91/6-31G(D) FREQ=NORAMAN NAME=SCHENKER GUESS=READ
 GEOM=CHECK\\deprotts\\0,1\S,2.0181544314,2.9941592529,-0.4818915701\N
,2.0917869436,0.5171997734,0.5752940677\N,0.3560893288,1.8239353075,1.
2646300154\N,0.391322588,-2.0265228297,-1.7496023725\N,-0.3604960197,0
.1458638602,-1.944887077\N,1.9185583846,-0.2697016126,-2.1492226903\C,
3.3012159118,0.175596799,-0.1446167432\C,3.0270440338,-0.7697188026,-1
.3358494173\C,4.3080043711,-0.9863925196,-2.1429806307\C,5.416985385,-
1.5565203337,-1.2527579991\C,5.6667731082,-0.6780312113,-0.023542204\C
,4.3789551104,-0.4204401319,0.7660222395\C,1.4626591224,1.7255451821,0
.4992889515\C,-0.439640261,3.0351875879,1.4316884877\C,-1.0572934667,3
.0151432852,2.833664951\C,-1.5077616961,3.2220634856,0.3651491542\C,-2
.3467403312,2.1683326717,-0.0103509486\C,-1.703181237,4.4801253212,-0.
211145909\C,-3.3591266776,2.3707357824,-0.9474449611\C,-2.7211340703,4
.6867170556,-1.1403183864\C,-3.5528794049,3.6312445575,-1.5112660946\C
,0.6485736295,-0.7453476421,-1.920348817\H,4.0979213028,-1.6552789026,
-2.9859903933\H,2.7116707522,-1.7393606474,-0.9296084685\H,3.669673594
9,1.1273868674,-0.5459600543\H,5.1310023203,-2.5670907563,-0.924394296
7\H,6.3412427686,-1.6701385253,-1.8319888557\H,6.4189413902,-1.1408349
267,0.6269726992\H,6.0837631006,0.2864645259,-0.3460737582\H,4.0161994
862,-1.3640622127,1.2035426696\H,4.5656984085,0.2669463627,1.598979008
2\H,4.6280299479,-0.0268530367,-2.5737097735\H,1.5936679116,-0.2226303
859,1.0620367988\H,-0.023488057,0.9612938864,1.6482525124\H,0.26192287
31,3.8717282888,1.3593916038\H,-1.637602366,3.9279196663,2.9986065188\
H,-1.7389977467,2.1642532251,2.9521120489\H,-0.2778371055,2.9503608354
,3.6009485621\H,-2.204288125,1.173545735,0.4048593216\H,-1.0435266028,
5.3013763399,0.0612729294\H,-3.988045511,1.5325188552,-1.2342184471\H,
-2.8557111605,5.6705204583,-1.5836088251\H,-4.3426864013,3.7888888723,
-2.2418159164\H,1.9547033202,0.7396602859,-2.2791493001\H,-0.163457776
,1.1090383565,-1.7000698515\H,-1.2811257646,-0.1922413955,-1.660067610
4\O,-2.774135899,-0.9982718209,-0.698798255\C,-2.6011916838,-2.1855601
062,-0.4397330678\C,-1.3944934416,-2.7977311699,0.1219496587\O,-0.5277
210351,-0.9732108772,1.4507901557\C,-0.7318187481,-2.1707072092,1.2620
115489\O,-0.2402872697,-3.1084952082,2.1087297049\C,0.503916344,-2.630
5332113,3.2355202988\C,0.8150474147,-3.823541973,4.1139732672\O,-3.521
629136,-3.1258038578,-0.7508715316\C,-4.7010620337,-2.674808747,-1.437
8232012\C,-5.7663223969,-2.2080114893,-0.4632118865\H,-6.681278063,-1.
9436491736,-1.0058661754\H,-6.0090288469,-2.9980708796,0.2549335003\H,
-5.4238336863,-1.3258444342,0.0847182452\H,-4.4266992233,-1.8769984439
,-2.1335767849\H,-5.0362023013,-3.5478490505,-2.0048216418\H,-0.085960
6561,-1.8756391085,3.7658786914\H,1.423247129,-2.1425883572,2.88641082
01\H,1.3910075365,-3.5045960899,4.9893416719\H,-0.1069585389,-4.299392
1227,4.4622104216\H,1.4009752193,-4.569799321,3.5680645572\H,-1.487776
5606,-3.8750865562,0.2390756267\H,1.1813704871,-2.6342172448,-1.933387
4491\H,-0.4968197078,-2.4754795013,-0.8825894259\\Version=IA64L-G03Rev

C.02\State=1-A\HF=-1871.0405724\RMSD=4.386e-09\RMSF=5.971e-06\ZPE=376.
2563738\Dipole=0.673967,-1.4683188,0.6845032\DipoleDeriv=-0.5582568,-0
01426\Polar=349.4573498,-7.4619519,321.2561429,21.4128421,-20.1888357,
260.4898297\PG=C01 [X(C23H37N5O4S1)]\NImag=1\\0.07490186,0.04982676,0.
.00736676,-0.00534093,0.00296118,0.04122123,0.02898499,0.01513604\\0.0
.00000880,0.00000531,0.00000395\\\@

Complex2

1\1\GINC-ALTIX2\Freq\RB3PW91\6-31G(d)\C23H37N5O4S1\SCHENKER\24-Jan-200
8\0\\#P Geom=AllCheck Guess=Read SCRF=Check GenChk RB3PW91/6-31G(d) Fr
eq\\deprotDFT-end.com\\0,1\S,1.5659446325,3.1877743012,-0.0810902746\N
,2.0465689414,0.6206328663,0.6006252826\N,0.1871711779,1.546044814,1.5
313150721\N,0.5946047197,-1.6626868418,-2.0080920582\N,-0.4695977837,0
.3654499976,-1.8069188233\N,1.8321620569,0.305139267,-2.1955706826\C,3
.2532101943,0.5826598243,-0.1939208768\C,3.0525695971,-0.1693335849,-1
.5282418105\C,4.2971350027,-0.0439598491,-2.4055234801\C,5.5223060069,
-0.5965769336,-1.6679323244\C,5.7137309815,0.0678213834,-0.3012734797\
C,4.4477390034,-0.0112000594,0.5588028898\C,1.2522241989,1.7236737934,
0.7272033808\C,-0.755401561,2.5887919563,1.9190927015\C,-1.2779335551,
2.2638134457,3.3225236609\C,-1.9021746701,2.7800611132,0.9386504256\C,
-2.5912644595,1.6857230862,0.4064288484\C,-2.3226137882,4.07076422,0.6
059519124\C,-3.6759809297,1.881456872,-0.4476900356\C,-3.414736633,4.2
681651865,-0.2368163698\C,-4.0947269335,3.1725700916,-0.7669862491\C,0
.6488878673,-0.3246771236,-1.9699087011\H,4.1371580151,-0.5736095796,-
3.352357019\H,2.9033727768,-1.2305388779,-1.2942003696\H,3.4745327614,
1.6306622618,-0.430175301\H,5.3998029246,-1.6814081696,-1.5308560486\H
,6.4188671674,-0.4629525734,-2.2847588737\H,6.5558260464,-0.3946420805
,0.2275619622\H,5.978941161,1.1245377791,-0.4462864752\H,4.2388997455,
-1.0595351572,0.8231825977\H,4.5838696484,0.5335244031,1.4996428545\H,
4.4552495026,1.0143320647,-2.6567735482\H,1.6398360712,-0.2564681713,0
.9296732131\H,-0.0511633069,0.5814809109,1.7642281031\H,-0.1818571278,
3.5199493412,1.9559942416\H,-1.965423138,3.0469502666,3.6559022589\H,-
1.829098264,1.3158109525,3.3274680276\H,-0.4510238069,2.1933551047,4.0
378208304\H,-2.2745291028,0.6697528071,0.630943487\H,-1.7820002699,4.9
280625608,1.0019185874\H,-4.1815557919,1.0146330806,-0.8638529369\H,-3
.7261112934,5.2791430771,-0.4887189128\H,-4.9421352969,3.3248346736,-1
.4310454144\H,1.7477884209,1.3214606744,-2.1646490144\H,-0.409355064,1
.3236642299,-1.4788515633\H,-1.3142948763,-0.1727622435,-1.5362348599\
O,-2.4890136607,-1.3164343949,-0.982385092\C,-2.3446137593,-2.51260506
68,-0.663164931\C,-1.3315363343,-3.1220279327,0.1240552866\O,-0.163890
6133,-1.2296157699,1.0455793155\C,-0.4468088774,-2.4450378992,0.995589
9638\O,0.1713632544,-3.3217655024,1.8506771615\C,1.0717393418,-2.76686
96645,2.8022517896\C,1.4505687802,-3.8689719356,3.7707010725\O,-3.2328
871708,-3.4477329408,-1.1178435066\C,-4.2891544547,-2.9902039366,-1.96
45165972\C,-5.5144148756,-2.5934560041,-1.1594518832\H,-6.3368971894,-
2.3174249614,-1.830112666\H,-5.8487186936,-3.423230322,-0.5279210887\H
,-5.2891086037,-1.7364651378,-0.5180132686\H,-3.9344931242,-2.15274030
17,-2.5720867897\H,-4.5106435149,-3.8391392021,-2.6196578145\H,0.59708
11685,-1.9261394456,3.3210792584\H,1.9635603808,-2.3732035516,2.292825
6192\H,2.1545416823,-3.4890948543,4.5194104938\H,0.5639940799,-4.24698
96886,4.2893409995\H,1.9215776445,-4.7060654234,3.2455005177\H,-1.4173
786442,-4.192392539,0.2659551858\H,1.4115193434,-2.1797323533,-2.28935
89572\H,-0.1175671285,-2.1772363327,-1.478208904\\Version=IA64L-G03Rev
D.01\State=1-A\HF=-1871.0520031\RMSD=6.696e-09\RMSF=1.531e-05\ZeroPoin
t=0.6041341\Thermal=0.6395171\Dipole=1.7716808,-0.6874995,-0.4639503\D
2417424,-0.1156651,0.4537434\Polar=358.9821013,-7.1488475,318.8306412,
31.7559474,-3.5435083,259.2432901\PG=C01 [X(C23H37N5O4S1)]\NImag=0\\0.
08,-0.14319039,-0.08380734,0.11651819\\0.00000560,-0.00000100,0.000006
000743\\\@

Complex2a

1\1\FAU-CCC-SNODE127\Freq\RB3PW91\6-31G(d)\C23H37N5O4S1\BCO166\26-Oct-
2007\0\\#P GEOM=ALLCHECK GUESS=READ SCRF=CHECK GENCHK RB3PW91/6-31G(D)
 FREQ\\SSR-MH30-malon-c3\\0,1\S,-2.6789905077,1.8053198523,1.380815893
7\N,-3.5405673627,0.669772145,-0.8934740131\N,-2.3201836221,2.58265320
08,-1.1608241852\N,-0.9391593588,-1.1294846461,1.0369407083\N,0.464324

2018,-1.8488768344,-0.636560814\N,-1.7819926323,-1.5948341312,-1.11299
56838\C,-3.9297884846,-0.5624126373,-0.235438977\C,-3.2125093489,-1.78
615625,-0.8512455564\C,-3.5383725167,-3.0501968705,-0.0447257141\C,-5.
0516776901,-3.2886568632,-0.0036890135\C,-5.8067084447,-2.0661770883,0
.5234970728\C,-5.4446711985,-0.7973845025,-0.2540866147\C,-2.837658645
1,1.679306733,-0.2993643121\C,-1.2932462624,3.5897606238,-0.8406831392
\C,-1.7979902393,4.9815808977,-1.2073271294\C,0.0132207901,3.162193101
,-1.4971930667\C,0.7450265105,2.1138052617,-0.9203666919\C,0.478575592
7,3.7295526411,-2.687173174\C,1.903008255,1.6300105289,-1.52309631\C,1
.6454466236,3.2553274201,-3.2893499303\C,2.3538763196,2.2033624217,-2.
7137318477\C,-0.7493432376,-1.5191030917,-0.228970999\H,-3.026801058,-
3.9080728571,-0.4977326478\H,-3.6361972123,-1.9209604615,-1.8579877634
\H,-3.6324494767,-0.4424325746,0.8107673318\H,-5.4057063705,-3.5256504
359,-1.0181079094\H,-5.270533995,-4.1679930432,0.6134012934\H,-6.88845
84917,-2.2383179673,0.4749489229\H,-5.5629227657,-1.9160243479,1.58452
54863\H,-5.7934972367,-0.8803115262,-1.2952292683\H,-5.937027704,0.079
8036104,0.1793765707\H,-3.1392959324,-2.9415121285,0.9714539331\H,-3.5
441618275,0.6473497479,-1.9062171897\H,-2.3734232207,2.3605485617,-2.1
48964304\H,-1.1752617089,3.5334343457,0.2452210499\H,-1.0329862824,5.7
341618816,-0.9907403721\H,-2.0612166243,5.060354428,-2.2685981856\H,-2
.6922005289,5.2146307427,-0.6217422582\H,0.4111258581,1.6698632086,0.0
148667721\H,-0.0584665663,4.5541359148,-3.1494049079\H,2.4404625835,0.
8020646444,-1.0673331067\H,1.998534004,3.7127780364,-4.2106843555\H,3.
260179519,1.8321072709,-3.1855503367\H,-1.4918847995,-1.8360229731,-2.
0488594068\H,1.3257869791,-1.5721463517,-0.1159688094\H,-0.0512546142,
-0.9862683675,1.5510660058\O,3.1426028548,-1.7247386132,-0.66879947\C,
4.0366919311,-1.6956553334,0.1979896667\C,3.9567000952,-1.2838067596,1
.54639283\O,1.6490482886,-0.6713382914,1.5716931958\C,2.7742179225,-0.
8074064964,2.1194385566\O,2.9225646231,-0.4493959729,3.4344122989\C,1.
774150465,0.0718489874,4.089473859\C,2.2047477692,0.5148148538,5.47393
52823\O,5.3064355059,-2.1036424395,-0.1370849621\C,5.4828292007,-2.543
2198731,-1.4771773611\C,6.9295725402,-2.9707771917,-1.6281583561\H,7.1
198510686,-3.3197407208,-2.6497287564\H,7.605433095,-2.1356143136,-1.4
165338883\H,7.1680118572,-3.7840712736,-0.9347829429\H,4.7971092991,-3
.3724466632,-1.6932740292\H,5.2316944415,-1.733320687,-2.1742079456\H,
4.8494810299,-1.3239285145,2.1561577616\H,0.9932865185,-0.6989834394,4
.1518763319\H,1.3593308821,0.9084043172,3.5143067914\H,1.3496586594,0.
9195415695,6.0271707646\H,2.6170488115,-0.3268436104,6.0400947962\H,2.
974338769,1.2908114911,5.4090817346\H,-1.6459016743,-0.4170500949,1.21
9902691\H,0.6234163912,-2.2827437349,-1.5326891828\\Version=AM64L-G03R
evC.02\State=1-A\HF=-1871.0298757\RMSD=4.002e-09\RMSF=1.900e-05\ZPE=37
8.0134707\Dipole=-4.2392139,-0.5010171,-3.1142609\DipoleDeriv=-0.44678
-0.0463698,0.1719287\Polar=352.9756016,-12.1967427,280.0327206,-35.267
031,-1.0847571,307.0678505\PG=C01 [X(C23H37N5O4S1)]\NImag=0\\0.0368743
0.00063087,-0.05606856,0.18225559,0.38594361\\0.00000037,0.00000935,0.
,0.00000369\\\@

CatN1

1\1\FAU-CCC-SNODE130\Freq\RB3PW91\6-31G(d)\C24H32N6O2S1\SCHENKER\28-Oc
t-2007\0\\#P GEOM=ALLCHECK GUESS=READ SCRF=CHECK GENCHK RB3PW91/6-31G(
D) FREQ\\SSH-MH30-nitrostyrene//HF/3-21G\\0,1\S,-1.3056385877,3.172063
0228,1.959841427\N,-0.9782475107,2.258928232,-0.5570505029\N,0.9437488
636,2.6489933943,0.5917405444\N,-1.1467899255,-1.608013235,0.241394630
1\N,-1.1023500521,-0.6522126856,2.4311563707\N,-2.4906689022,0.2084939
32,0.8092071493\C,-2.3957874582,2.057087714,-0.8077871065\C,-2.8108954
315,0.5883262834,-0.552130676\C,-4.3001696304,0.3920802328,-0.86168279
86\C,-4.6423502932,0.819158957,-2.2895001136\C,-4.2247379273,2.2688381
985,-2.5465848554\C,-2.7414619921,2.4856986984,-2.2380665043\C,-0.4138
741904,2.6590735031,0.6042460864\C,1.795186422,2.8977209058,1.75016749
83\C,3.114509495,3.5104289282,1.2780268295\C,2.0082852036,1.6426932464
,2.5883596133\C,1.6175945835,1.6201494598,3.9302681596\C,2.5992424212,
0.496974247,2.042528538\C,1.8141071693,0.4825773085,4.7137415241\C,2.7
946814553,-0.6423339588,2.8209869566\C,2.4028267792,-0.6535651013,4.16
10681959\C,-1.5269711339,-0.7216677197,1.1085703456\H,-4.5551044373,-0
.659979019,-0.6909073022\H,-2.2223024109,-0.0664532198,-1.2060088311\H
,-2.9301091549,2.700672317,-0.0984725969\H,-4.1230188462,0.1600438441,
-3.0010308546\H,-5.7163143505,0.693165498,-2.4757737925\H,-4.432223451

8,2.5516512626,-3.5862494298\H,-4.8270631212,2.937885815,-1.9149444138
\H,-2.1329347577,1.902096173,-2.9455152819\H,-2.464284821,3.5379625947
,-2.3735701302\H,-4.8934553555,0.9801810483,-0.1458537512\H,-0.3569666
425,1.8438891454,-1.2483382209\H,1.4093501146,2.2836168784,-0.23399627
77\H,1.2601184276,3.6269137856,2.3650049208\H,3.7571465593,3.723884443
5,2.1376328812\H,3.6611726811,2.8300923692,0.614430087\H,2.9288796731,
4.4446058368,0.7377120363\H,1.1422919567,2.4989127844,4.3591018974\H,2
.9054473792,0.4893710395,0.9986832077\H,1.5013418228,0.4835133607,5.75
49855116\H,3.260866295,-1.5220697779,2.3830290304\H,2.5588707037,-1.54
09162958,4.7696481155\H,-2.5804005833,0.9643359778,1.4810153284\H,-1.0
966186741,0.2828511866,2.8287307533\H,-0.2208606917,-1.1170760878,2.60
56646042\H,-0.446584101,-2.213730549,0.6688132699\C,-0.1226390891,-5.7
226244351,-1.7223828456\C,-0.1149439554,-4.3317911527,-1.7081186504\C,
1.030798208,-3.6228163238,-2.1133377081\C,1.0043515178,-6.4221319234,-
2.1519094877\C,2.1447355462,-5.72802801,-2.5664744416\C,2.1608200953,-
4.3404411349,-2.5467637644\H,-0.9811736237,-3.7722554699,-1.3610612363
\H,-1.0097830149,-6.2611442108,-1.4006936691\H,0.9960673309,-7.5088626
281,-2.1697998467\H,3.0188608316,-6.2742666401,-2.9103845579\H,3.04475
45378,-3.8085851015,-2.8890068661\C,0.9758405864,-2.171868989,-2.06790
61406\C,2.02272666,-1.3276571516,-2.1253146143\H,-0.0041057397,-1.7290
799475,-1.891542834\N,1.7852845096,0.07685438,-2.0050328523\O,2.722771
8081,0.7787142087,-1.6128209033\O,0.6689689221,0.5226664441,-2.2822045
895\H,3.0763379,-1.5718788183,-2.138492257\\Version=AM64L-G03RevC.02\S
tate=1-A\HF=-1810.3317668\RMSD=2.396e-09\RMSF=2.917e-05\ZPE=344.320256
\Dipole=1.4229983,-2.1614795,-1.0830238\DipoleDeriv=-0.6196797,0.10208
-0.0433314,-0.0926641,0.17123\Polar=302.0139914,-20.3099069,397.922223
1,5.4359407,24.0921952,277.2352454\PG=C01 [X(C24H32N6O2S1)]\NImag=0\\0
4,0.00016947,-0.00019124,0.00351517,-0.00441128,0.00339774,0.02097062\
\0.00002108,0.00000276,0.00001682,0.00000247,0.00007340,-0.00007905,0.
00000037\\\@

CatN2

1\1\FAU-CCC-SNODE126\Freq\RB3PW91\6-31G(d)\C24H32N6O2S1\SCHENKER\25-Oc
t-2007\0\\#P GEOM=ALLCHECK GUESS=READ SCRF=CHECK GENCHK RB3PW91/6-31G(
D) FREQ\\ssr-mh30-nist-c4\\0,1\S,-3.2453959396,2.6241428143,0.91735805
92\N,-2.601251671,0.4391738669,-0.5040671187\N,-0.970573573,2.01877283
73,-0.3693663923\N,-3.237397501,-1.8684032257,2.4438699757\N,-0.991890
9355,-2.6214705526,2.0636797557\N,-2.4563412776,-2.3390227345,0.302603
2198\C,-3.8333925183,-0.2742756477,-0.1912781399\C,-3.6076115656,-1.78
77051933,-0.4050602513\C,-4.8940333356,-2.5713960539,-0.1097000543\C,-
6.0690772072,-2.0711893839,-0.9513511229\C,-6.2837205513,-0.5693160998
,-0.758996278\C,-5.002375608,0.2081260082,-1.0613592475\C,-2.239199752
5,1.6508680099,-0.0233424223\C,-0.3809441968,3.3257765897,-0.124336027
7\C,-0.6297796143,4.2746066789,-1.296481425\C,1.0775347459,3.141062822
9,0.2704557746\C,1.3787503388,2.318140154,1.366743389\C,2.1329653332,3
.7671823121,-0.3980514323\C,2.6922212288,2.1367947926,1.7867248336\C,3
.4538424536,3.5861361423,0.0195236018\C,3.7385404714,2.7739212665,1.11
42537851\C,-2.2913651362,-2.2596739334,1.6686444662\H,-4.7004415329,-3
.6341469676,-0.3010872939\H,-3.3707632381,-1.9343832415,-1.4712683628\
H,-4.0616284772,-0.1022456116,0.8656251675\H,-5.8750712761,-2.27490325
83,-2.016061515\H,-6.9774620739,-2.628794845,-0.6889457417\H,-7.099936
9413,-0.2112114733,-1.3994589406\H,-6.5892463569,-0.3736314777,0.27849
38343\H,-4.7315538281,0.0814476722,-2.121199111\H,-5.1421612381,1.2800
362698,-0.8880691132\H,-5.1163214279,-2.4691288781,0.9575840562\H,-1.8
985279194,-0.0791253242,-1.0197216997\H,-0.4302291415,1.3807537786,-0.
9420055547\H,-0.924003544,3.7217557925,0.7440359862\H,-0.2458151948,5.
2790804298,-1.0852424495\H,-0.1662185099,3.9112090692,-2.2217612196\H,
-1.708753245,4.3512691782,-1.4580639961\H,0.5648071426,1.823919961,1.8
920433294\H,1.9307448334,4.411261745,-1.2488114416\H,2.9013257247,1.50
62182108,2.6476072573\H,4.2581598842,4.0918327577,-0.509931473\H,4.764
2718484,2.6465344818,1.4524006503\H,-1.5951309106,-2.3719148559,-0.227
6283299\H,-0.568078459,-3.3405796799,1.4881690703\H,-0.9344760672,-2.8
63684173,3.0444352851\H,-2.9480631508,-1.9124068608,3.4201857015\C,6.8
014123416,-2.8309915344,-0.022259185\C,5.4308560912,-2.9631641992,-0.2
115862624\C,4.6186898967,-1.8320420746,-0.408967441\C,7.3811631359,-1.
5631399196,-0.0195417895\C,6.5849497028,-0.4293458116,-0.2034366199\C,
5.2167784158,-0.5573999178,-0.3963624528\H,4.9761347652,-3.9510253794,

-0.2136398418\H,7.4161659612,-3.7144010646,0.1250843586\H,8.4519765414
,-1.45523883,0.1309275748\H,7.0363081381,0.5587959489,-0.191687256\H,4
.6088931688,0.3349281132,-0.5142111089\C,3.1969352289,-2.0395034167,-0
.6154254376\C,2.2871159388,-1.1120973527,-0.962023232\H,2.8258408498,-
3.055028344,-0.4917498957\N,0.9160668212,-1.4844563492,-1.1377493657\O
,0.1625393421,-0.6088853485,-1.5784252361\O,0.5435302595,-2.62507693,-
0.865495228\H,2.4552138281,-0.0621822712,-1.1559258567\\Version=AM64L-
G03RevC.02\State=1-A\HF=-1810.3167779\RMSD=2.407e-09\RMSF=6.072e-06\ZP
E=343.757065\Dipole=5.0107801,-2.0961556,-0.8835587\DipoleDeriv=-0.804
24785,0.0331171,-0.0605777,0.0607281,0.1460147\Polar=489.1004044,-8.68
28772,299.9002463,23.6352612,-20.6672333,216.3537474\PG=C01 [X(C24H32N
6O2S1)]\NImag=0\\0.15087115,-0.08253632,0.13595883,-0.09911185,0.09748
.03453144\\0.00000050,0.00000042,-0.00000011,0.00000217,-0.00000246,0.
.00000184,0.00000120,0.00000039\\\@

CatN3

1\1\FAU-CCC-SNODE133\Freq\RB3PW91\6-31G(d)\C24H32N6O2S1\SCHENKER\27-Oc
t-2007\0\\#P GEOM=ALLCHECK GUESS=READ SCRF=CHECK GENCHK RB3PW91/6-31G(
D) FREQ\\ssr-mh30-nitrostyrene//B3PW91/6-31G(d)\\0,1\S,-2.0095072348,4
.6139456083,0.9572048725\N,-1.9299877411,1.9378817905,0.6484223157\N,-
0.4941928555,3.1141425834,-0.665586523\N,-0.7827663369,0.989436181,4.0
360673568\N,0.6533349108,-0.7585392418,3.2475856863\N,-1.3356483143,-0
.3010365977,2.1752629264\C,-2.8147536006,1.6729324163,1.7807398936\C,-
2.7204193916,0.1659692886,2.0963307333\C,-3.5974899698,-0.1878810087,3
.304426046\C,-5.0550845137,0.2127700424,3.0605513006\C,-5.1697624771,1
.6896674925,2.6773671888\C,-4.2753221713,2.0287025843,1.4832818851\C,-
1.4671214034,3.1668656813,0.2945138573\C,0.0690240509,4.2749482352,-1.
3464386304\C,-0.8073952861,4.7090522294,-2.5208630246\C,1.5234466967,3
.982342873,-1.6883303216\C,2.3967332005,3.6032559238,-0.6585146801\C,2
.0336774355,4.0894137045,-2.9843371768\C,3.7379769778,3.3420312077,-0.
9174373233\C,3.3795572722,3.8266058524,-3.2478273763\C,4.2360568742,3.
4521654247,-2.2170877697\C,-0.4852277588,0.0446372098,3.2191751262\H,-
3.5223991461,-1.2667965364,3.4944832369\H,-3.1267896213,-0.3608922451,
1.218422707\H,-2.4628508258,2.2411172549,2.6498236683\H,-5.4733440932,
-0.4028201794,2.2490234976\H,-5.6560316491,-0.0019127891,3.9537325023\
H,-6.2122038555,1.9448357486,2.4479700175\H,-4.8764482754,2.3130846318
,3.5338162693\H,-4.6030891037,1.4694002352,0.5938068793\H,-4.339928260
9,3.0931835278,1.2412476164\H,-3.1958928774,0.3256429349,4.1843164451\
H,-1.2795419636,1.1677466518,0.5032488615\H,-0.3673582072,2.2293408313
,-1.1423136387\H,0.041369087,5.0780722405,-0.5992295348\H,-0.423642665
4,5.6245291622,-2.9846737088\H,-0.8716233965,3.9306144795,-3.291134085
6\H,-1.8144268468,4.9145335281,-2.1471594809\H,2.0094075927,3.51554450
26,0.3539065807\H,1.382329254,4.3842632076,-3.8017990633\H,4.399248602
,3.0563254883,-0.102713667\H,3.7552878989,3.917202717,-4.2642919503\H,
5.2847417723,3.2503446175,-2.4211867199\H,-1.2099940973,-1.2545061727,
1.8517870322\H,0.9454712812,-1.1459786983,2.3555117882\H,1.4230352381,
-0.3679819543,3.7731634085\H,-0.0178797949,1.155208186,4.6897658557\C,
4.181697203,-6.5662046113,-3.183904982\C,3.708823271,-5.5664943628,-2.
3413386934\C,2.3645725987,-5.5468713204,-1.9302094272\C,3.3166919593,-
7.563683607,-3.6302170964\C,1.9772305972,-7.5576841734,-3.2308702133\C
,1.5035967017,-6.5612395832,-2.3898215075\H,4.3824116438,-4.7874314105
,-1.9922447393\H,5.2233233606,-6.5669930322,-3.4921829965\H,3.68249431
75,-8.3465974839,-4.2890059907\H,1.302761268,-8.3349630333,-3.57898489
31\H,0.4601546196,-6.5700830658,-2.0880909801\C,1.934370417,-4.4754309
525,-1.0479405264\C,0.7037630783,-4.2885938539,-0.5431491418\H,2.68664
5114,-3.7427764047,-0.7620528274\N,0.4346274056,-3.1736611866,0.321080
6743\O,-0.720651509,-3.0838982605,0.7402227333\O,1.3378076788,-2.38222
78044,0.5948492053\H,-0.1757900202,-4.8966030117,-0.7012314532\\Versio
n=AM64L-G03RevC.02\State=1-A\HF=-1810.3154797\RMSD=5.616e-09\RMSF=1.17
9e-05\ZPE=343.9419253\Dipole=2.6474444,-4.9809638,-2.6875203\DipoleDer
17534,0.0466431,-0.0793858,0.0258003,-0.0683351,0.1088343\Polar=373.17
01181,-42.1266924,318.69212,-81.4157688,59.0931909,308.7435514\PG=C01
[X(C24H32N6O2S1)]\NImag=0\\0.07127811,-0.07196346,0.25343420,-0.055930
0.00039459,0.03180134,0.04408853,0.04255104\\0.00000066,-0.00000029,0.
00001710,0.00001393,-0.00000101,-0.00000161,-0.00000112\\\@

CatN4

1\1\FAU-CCC-SNODE131\Freq\RB3PW91\6-31G(d)\C24H32N6O2S1\SCHENKER\28-Oc
t-2007\0\\#P GEOM=ALLCHECK GUESS=READ SCRF=CHECK GENCHK RB3PW91/6-31G(
D) FREQ\\ssr-mh30-nist-c6\\0,1\S,-0.8306210198,-2.648177093,-1.0204152
516\N,1.0278555,-2.6239112111,0.9277176517\N,1.6113102294,-1.542539647
5,-0.9862460821\N,-0.9880289023,-0.7102148064,3.3674695792\N,0.8930393
344,0.6723638041,3.9144978416\N,1.1796233356,-1.578505504,3.4909168653
\C,0.1329239942,-3.211464985,1.9226029876\C,0.7551499223,-2.970700654,
3.3122306847\C,-0.1555695428,-3.5296217409,4.4132763327\C,-0.415487350
2,-5.0235713481,4.20122836\C,-0.9737025687,-5.2981137688,2.8030355372\
C,-0.0663200477,-4.7169317458,1.7169418443\C,0.6531764254,-2.264241165
,-0.3290916335\C,1.5193843682,-1.1045120517,-2.3719200366\C,2.03550586
25,-2.1817701508,-3.3273800853\C,2.1804891959,0.2610032738,-2.51419406
94\C,1.9959589381,1.2273133726,-1.5142673688\C,2.9434369718,0.60953520
54,-3.6334567077\C,2.5528216297,2.4987188497,-1.630766101\C,3.49920933
28,1.8841482402,-3.7550627647\C,3.3072241975,2.8345267982,-2.756074044
9\C,0.2623409138,-0.5240876747,3.5727543973\H,0.317489037,-3.358959042
,5.3899835603\H,1.6982002447,-3.5407943866,3.333312391\H,-0.8353942856
,-2.7002601544,1.8734247476\H,0.5255651989,-5.580789052,4.3290603926\H
,-1.1033474223,-5.3954762421,4.9712819177\H,-1.0994251595,-6.376964962
8,2.6468835116\H,-1.9734127278,-4.850226532,2.7145910583\H,0.915946979
4,-5.2127823762,1.7405781818\H,-0.492544871,-4.8838879267,0.723278796\
H,-1.0934075771,-2.9649801327,4.4042960676\H,1.7275674467,-2.026051539
5,1.361866968\H,2.5418770677,-1.5381597617,-0.5857527165\H,0.442254474
3,-0.9917952328,-2.5542730377\H,1.8424152819,-1.916610227,-4.372380441
\H,3.1128543083,-2.3478814561,-3.2060522789\H,1.506604945,-3.115854172
7,-3.1187689905\H,1.4159384799,0.9745376735,-0.6306966316\H,3.11384508
68,-0.1152549277,-4.4237366738\H,2.401431395,3.2224040026,-0.833355645
6\H,4.0907783293,2.1285779301,-4.6341285882\H,3.7471326674,3.824483219
9,-2.8477149825\H,1.9147120499,-1.4838726646,4.1825003121\H,1.80183111
98,0.7919270035,3.4812939682\H,0.3128021843,1.4897026218,3.7479084732\
H,-1.482153048,0.1819485947,3.3588559214\C,-2.3898652438,3.6866343766,
-4.2432992878\C,-1.6334631188,3.4562223867,-3.099548755\C,-2.238324266
7,3.4382333721,-1.830492604\C,-3.762110136,3.9053840141,-4.1360857508\
C,-4.3766026816,3.8925477471,-2.8807549865\C,-3.6247292427,3.661548338
6,-1.738016757\H,-0.5635143213,3.2791346801,-3.1790881513\H,-1.9091991
212,3.6933507073,-5.2175160583\H,-4.3560633085,4.0851265499,-5.0282624
09\H,-5.4464326752,4.0626545826,-2.7972879572\H,-4.1152107984,3.655216
8426,-0.7688174985\C,-1.3914763805,3.1874796865,-0.677111138\C,-1.7801
863302,3.0722337658,0.6035176433\H,-0.3279288341,3.0690902204,-0.87278
25908\N,-0.8151549854,2.8099123412,1.6324029781\O,-1.2683504197,2.6636
521638,2.7738459916\O,0.3814170378,2.7428569026,1.3592511989\H,-2.7850
294091,3.1283874287,0.9976588721\\Version=AM64L-G03RevC.02\State=1-A\H
F=-1810.3158802\RMSD=1.177e-09\RMSF=1.194e-05\ZPE=344.2478858\Dipole=0
.8133554,1.9196062,-1.1761684\DipoleDeriv=-0.7787501,-0.2299486,-0.157
0.0369458,0.0069757,0.156822,0.0339672,0.0155282,0.0080943,0.0639351\P
7\PG=C01 [X(C24H32N6O2S1)]\NImag=0\\0.26280143,0.06871793,0.04669132,0
09994518\\-0.00001975,0.00000244,-0.00000715,0.00000719,-0.00000695,-0
.00000172\\\@

CatN5

1\1\FAU-CCC-SNODE122\Freq\RB3PW91\6-31G(d)\C24H32N6O2S1\SCHENKER\26-Oc
t-2007\0\\#P B3PW91/6-31G(D) FREQ=NORAMAN NAME=SCHENKER GEOM=CHECK\\SS
R-MH30 + Nitrostyrene//B3PW91/6-31G(d)\\0,1\S,1.4939765573,-0.02084114
73,-3.2013176064\N,1.9096287931,-1.2243210122,-0.8431893852\N,1.996944
1573,1.0548527831,-0.7912847341\N,-1.2154106175,-3.5544577941,-1.29245
54791\N,-1.926030992,-2.9912262065,0.943586187\N,0.3008706746,-3.30567
32702,0.4675435895\C,1.8412978277,-2.566431997,-1.4132297551\C,1.50799
44714,-3.5879543791,-0.3030979943\C,1.4947850978,-5.0176626921,-0.8631
585005\C,2.80992029,-5.3745776433,-1.5559104111\C,3.1380968617,-4.3643
282435,-2.6550475756\C,3.167054269,-2.9442826699,-2.092361647\C,1.8175
757471,-0.0668756471,-1.5433725349\C,2.0413862691,2.4216487773,-1.2977
92883\C,3.3862752651,3.056835057,-0.9505387931\C,0.817191711,3.2115245
706,-0.8477341639\C,-0.4426068423,2.8096837136,-1.3164768863\C,0.89177
63654,4.3283531727,-0.0119161556\C,-1.5908111777,3.5128119973,-0.96690
44259\C,-0.2599003157,5.0348829619,0.3427448585\C,-1.5038106701,4.6329
390024,-0.1367837471\C,-0.9687621809,-3.3051889168,-0.0603602315\H,1.2

906284587,-5.70684249,-0.03428807\H,2.3208504404,-3.537724538,0.439506
7377\H,1.0310078195,-2.5687683783,-2.1487629226\H,3.6277149708,-5.3869
442378,-0.8190188161\H,2.7466291129,-6.3899100891,-1.9678639001\H,4.10
17321256,-4.6016319753,-3.1233944716\H,2.3792147065,-4.4253888596,-3.4
477583723\H,3.9784482285,-2.8566956744,-1.3526268519\H,3.3675488231,-2
.2126865532,-2.8815881684\H,0.6622945172,-5.1030975352,-1.569316607\H,
2.1789259339,-1.1605835847,0.1329542896\H,2.1108662722,0.9596792582,0.
2126768299\H,1.9776892468,2.3103082584,-2.3868137416\H,3.4545073047,4.
0734291141,-1.3524472047\H,3.5510629712,3.1065856912,0.1329498409\H,4.
1925832156,2.4604886595,-1.3877070803\H,-0.5058578247,1.9439992717,-1.
9727864428\H,1.8534080861,4.6657538979,0.3641301175\H,-2.5564254791,3.
1935099768,-1.3514841322\H,-0.1776225633,5.9081824573,0.9860095519\H,-
2.3994218787,5.1924330589,0.1230750941\H,0.430650891,-2.6397812963,1.2
206685347\H,-1.7647263212,-3.5276862931,1.7916348503\H,-2.87234464,-3.
1496871645,0.6160556426\H,-2.2186485108,-3.6137965472,-1.4639946014\C,
-4.8581120835,2.9482409779,2.5578191892\C,-3.4819870732,2.7507244073,2
.5369552182\C,-2.9427164358,1.4768418549,2.2876838045\C,-5.716918166,1
.8767248578,2.3186969235\C,-5.1942887692,0.6071944827,2.0566852426\C,-
3.8216090595,0.4048588878,2.0418271147\H,-2.8081735044,3.5854334525,2.
7109191979\H,-5.2605709116,3.9377773909,2.7558834611\H,-6.7929217864,2
.0289191955,2.3290319056\H,-5.8648391773,-0.2246370073,1.8585765613\H,
-3.4231751707,-0.5806243144,1.8166202604\C,-1.4981103782,1.3270533877,
2.2956743494\C,-0.8257085191,0.1639628497,2.2313270439\H,-0.9049059752
,2.2366952746,2.3633606466\N,0.602984032,0.1637929295,2.2316753412\O,1
.1599930339,-0.9440274072,2.1747825981\O,1.2277780686,1.2220486279,2.2
702436318\H,-1.2429004758,-0.8300644556,2.1271728504\\Version=AM64L-G0
3RevC.02\State=1-A\HF=-1810.3152246\RMSD=3.898e-09\RMSF=7.392e-06\ZPE=
344.2200451\Dipole=-2.4099513,0.2337456,2.9765612\DipoleDeriv=-0.33977
760422,-0.0238059,0.0553852,0.1942002\Polar=384.2843458,-53.2344868,35
5.7200523,-29.1600343,50.020873,231.3071152\PG=C01 [X(C24H32N6O2S1)]\N
Imag=0\\0.03138204,-0.00099685,0.06506557,0.05496116,-0.00415611,0.303
74900,-0.00001563,0.00413982,0.01066654,0.02708009,0.02507627\\-0.0000
0001571,-0.00001493,0.00001707,0.00001369,-0.00000932,0.00000626\\\@

Init1

1\1\GINC-A04\Freq\RB3PW91\6-31G(d)\C31H44N6O6S1\SHUBINA\10-Nov-2007\0\
\#P Geom=AllCheck Guess=Read SCRF=Check GenChk RB3PW91/6-31G(d) Freq\\
Init TS1\\0,1\S,-0.5918336994,5.4216509415,2.0540517686\N,0.4641721685
,3.0253089904,1.4407877447\N,-1.8077142038,3.0829630756,1.5006403408\N
,0.4305083961,5.0257950453,-1.047667841\N,0.2459894176,3.4537377533,-2
.7324200777\N,1.8185281929,3.1089392305,-1.1099620441\C,1.802526099,3.
5681715588,1.4060141075\C,2.5612070845,3.0819931675,0.1592728206\C,3.9
182893527,3.7888714873,0.0556209254\C,4.7607214094,3.5018261016,1.3023
430999\C,4.0164374678,3.8757220216,2.5864450441\C,2.6321934745,3.22323
37314,2.6512750673\C,-0.6493691643,3.7606723638,1.6507601501\C,-3.0964
571602,3.743799003,1.3244027957\C,-4.214185377,2.7686033582,1.69218218
06\C,-3.2417357315,4.2916337039,-0.0930139526\C,-3.1346677363,3.445253
3335,-1.2048717888\C,-3.4598278377,5.6566045425,-0.3063526187\C,-3.260
5784686,3.9565475231,-2.4976919631\C,-3.5759076125,6.1712499697,-1.598
8512621\C,-3.4789983147,5.322083759,-2.7008982059\C,0.8240814356,3.857
2559733,-1.5777560574\H,4.4394122867,3.4466253569,-0.8467079873\H,2.73
78432654,2.0094063799,0.3079274081\H,1.7036342427,4.660261435,1.396519
4181\H,5.0080902908,2.4304652106,1.3237934546\H,5.7140282065,4.0407352
481,1.2402424649\H,4.6083749344,3.5897869295,3.4645836168\H,3.89885075
8,4.9680469464,2.6335427959\H,2.7168673582,2.1309419915,2.7295747007\H
,2.0801652386,3.5713751418,3.5311295462\H,3.7592282634,4.8705585322,-0
.0583050175\H,0.3827370354,2.0202916228,1.2258624045\H,-1.7436997912,2
.1316921414,1.1172616027\H,-3.1122973754,4.5905368406,2.0165069528\H,-
5.1915705211,3.2419482985,1.5549712545\H,-4.1734738396,1.8714808598,1.
0660334939\H,-4.115252232,2.4608225605,2.7383508048\H,-2.9287345757,2.
3868246215,-1.0556429702\H,-3.5190602647,6.3235865127,0.5498938261\H,-
3.2052144901,3.2820458012,-3.3507199939\H,-3.74146293,7.2361801218,-1.
7432698179\H,-3.5813003903,5.7178782682,-3.7086263255\H,2.0544408607,2
.3179296008,-1.7141374154\H,0.3021400635,2.4539692209,-2.9383574546\H,
-0.640260189,3.8816404839,-2.9680527837\H,-0.49219448,5.3503096891,-1.
3121626568\O,-1.893915282,0.61604254,0.0259208047\C,-1.6959335817,-0.6

066560117,-0.0361678455\C,-0.5989491318,-1.378635399,0.411119138\O,0.9
969518952,0.3283220737,0.9887583809\C,0.6108773223,-0.8503395055,0.921
3509294\O,1.4437148703,-1.8565091796,1.3442786114\C,2.6881451911,-1.45
5115675,1.9060327208\C,3.4149608428,-2.7118845071,2.3416698052\O,-2.64
66738031,-1.4173707593,-0.6312693378\C,-3.8516752796,-0.7836920732,-1.
0490451138\C,-4.7589423689,-1.8526052209,-1.6248238173\H,-5.7066088409
,-1.408905849,-1.949689386\H,-4.2948180224,-2.3370878537,-2.4908275121
\H,-4.9774808472,-2.6219263462,-0.8770253534\H,-4.3242878618,-0.285431
5849,-0.194026168\H,-3.6289533151,-0.0102274546,-1.7940718768\H,-0.715
3304522,-2.4537707677,0.4301020189\H,2.5134859363,-0.781900431,2.75427
21202\H,3.2717681542,-0.8944704584,1.1645579702\H,4.3870017443,-2.4567
636335,2.7785871119\H,2.8318532256,-3.2571441603,3.0907637226\H,3.5825
753203,-3.3796869223,1.4901446761\H,0.6234119359,5.2518891485,-0.07156
44091\C,-0.7384136705,-5.6335812811,-1.6889750539\C,-0.6897353589,-4.2
542901936,-1.851077412\C,0.5329692437,-3.6033104657,-2.0963212133\C,0.
4314585045,-6.3880737531,-1.7745028925\C,1.6521579288,-5.7556527344,-2
.0208154562\C,1.7047668923,-4.3775437473,-2.1800572466\H,-1.5932113952
,-3.6569736381,-1.7594489692\H,-1.6894231282,-6.120984269,-1.492438769
5\H,0.3945272708,-7.4671793101,-1.6499292313\H,2.5644009133,-6.3419610
484,-2.0911137289\H,2.6600645268,-3.9018522451,-2.3829130483\C,0.51901
48514,-2.1618368013,-2.280637762\C,1.6120180378,-1.3761815475,-2.43439
64802\H,-0.4520845824,-1.6843106156,-2.3568805136\N,1.4705568791,0.008
9394474,-2.6359080914\O,2.5091852198,0.6970059698,-2.5938955915\O,0.35
118312,0.5004912404,-2.8429319357\H,2.6457341771,-1.6718314268,-2.3303
833498\\Version=IA64L-G03RevD.01\State=1-A\HF=-2385.0124264\RMSD=3.321
e-09\RMSF=3.811e-05\ZeroPoint=0.7427878\Thermal=0.7885751\Dipole=0.710

TS1

1\1\GINC-A09\Freq\RB3PW91\6-31G(d)\C31H44N6O6S1\SHUBINA\09-Nov-2007\0\
\#P B3PW91/6-31G(d) geom=check gues=read Freq=NoRaman Name=SHUBINA\\Ma
20Nc10\\0,1\S,-4.2952136649,1.0978971576,2.0121500045\N,-1.804719205,1
.5121228015,1.0856104415\N,-2.1126683292,-0.4657665361,2.1689572933\N,
-3.8327963438,0.5874553446,-1.234933339\N,-2.2592372402,-0.5274302079,
-2.5178587894\N,-1.7589905784,1.5791392764,-1.8030107225\C,-2.19711453
82,2.7401509893,0.4315630935\C,-1.6518859347,2.8055415328,-1.005875681
4\C,-2.223374407,4.032681024,-1.7285359217\C,-1.8443718992,5.314659628
7,-0.9804653888\C,-2.2744766709,5.2635666633,0.4874765703\C,-1.7583349
382,4.0025434951,1.1873103373\C,-2.6581637953,0.6958626334,1.746131592
1\C,-2.9095968078,-1.6121276927,2.5945635319\C,-2.0540848371,-2.517224
1074,3.4809292387\C,-3.4938673183,-2.3544214733,1.3957065377\C,-2.6615
169341,-2.8564012203,0.3866474129\C,-4.8758182144,-2.5308115536,1.2754
258047\C,-3.2030366142,-3.5281817649,-0.7101756556\C,-5.4201305995,-3.
1976214107,0.1764950179\C,-4.5852337747,-3.6999011468,-0.8209362878\C,
-2.6133288324,0.5616266435,-1.8044526734\H,-1.8394067235,4.0583040593,
-2.7551177767\H,-0.5654747801,2.9399794202,-0.9224121133\H,-3.29342112
46,2.7548313325,0.4287670391\H,-0.7540396466,5.4479173381,-1.036029177
1\H,-2.2895015193,6.1835852392,-1.4802338958\H,-1.9238683551,6.1578324
187,1.0172684529\H,-3.3723704956,5.2789156062,0.5460009962\H,-0.661679
4926,4.0162002079,1.256103334\H,-2.1482770192,3.9380933911,2.209085402
7\H,-3.3163931585,3.9387842449,-1.7982320037\H,-0.8236378883,1.2324430
672,0.9804095149\H,-1.1621075734,-0.6808894385,1.8551626948\H,-3.74103
36433,-1.2125232256,3.1819850812\H,-2.6341943987,-3.3876351248,3.80310
48216\H,-1.1693778667,-2.8729828403,2.9428059155\H,-1.7191517176,-1.96
93733561,4.3679618117\H,-1.5862645088,-2.7042224164,0.4527426947\H,-5.
5310295481,-2.1247641433,2.042017813\H,-2.5415775898,-3.933442384,-1.4
739716225\H,-6.4976629709,-3.3210742121,0.099517822\H,-5.006047042,-4.
2269817438,-1.6739026366\H,-0.9240635324,1.4197927788,-2.389009248\H,-
1.2485724444,-0.6755587851,-2.6401462103\H,-2.807138629,-1.3657052145,
-2.3802453966\H,-4.2505929144,-0.3125590269,-1.0316524737\O,0.39350018
6,-1.5353691821,1.1071912356\C,1.6105090097,-1.5708046684,0.9314062491
\C,2.5495852129,-0.4829856767,0.8158975626\O,1.0355775657,1.3804621002
,0.7147640973\C,2.1462992991,0.8915836731,0.9252261897\O,3.2122249404,
1.6874603513,1.2142575538\C,2.9456964781,3.0894043831,1.3111667574\C,4
.2346650497,3.7704196952,1.7215184555\O,2.2635372493,-2.7635954164,0.8
100511736\C,1.468400303,-3.9457971869,0.9348830149\C,2.3939328075,-5.1
367385104,0.7974885205\H,1.8246623966,-6.0670433328,0.8993437764\H,2.8
841477381,-5.141581842,-0.181656794\H,3.1685175991,-5.1200416877,1.570

7612872\H,0.9628268231,-3.942888082,1.9072134125\H,0.6937741227,-3.948
0191619,0.159883423\H,3.5639351072,-0.7090457277,1.1171924624\H,2.1506
27844,3.2572990822,2.0464959632\H,2.5801950854,3.4598555351,0.34597167
26\H,4.0782393955,4.8513355963,1.8067768856\H,4.5811620271,3.394304497
,2.6892686357\H,5.0244083805,3.5927198364,0.9845772511\H,-4.0373556677
,1.252640878,-0.4925719471\C,6.6771275639,-1.827443052,-0.9019431624\C
,5.2937532364,-1.7760980861,-1.0348709923\C,4.6333463498,-0.5544406521
,-1.2432431631\C,7.4292310006,-0.654936237,-0.9656753371\C,6.785058429
,0.5676529489,-1.1573913801\C,5.4021407909,0.619397121,-1.2930807678\H
,4.7061341458,-2.687947433,-0.9701814237\H,7.1693128797,-2.7842976587,
-0.747899176\H,8.5105749351,-0.6920647265,-0.86208735\H,7.364199259,1.
4865993543,-1.1996578874\H,4.9154836188,1.5805921018,-1.4259088626\C,3
.1751976239,-0.5632244349,-1.4274562654\C,2.522803768,0.4349466692,-2.
1367354051\H,2.7041033948,-1.5395987759,-1.4345811757\N,1.2185865042,0
.2846860779,-2.5269913361\O,0.6653947638,1.242496565,-3.1372388937\O,0
.5940133705,-0.7788528777,-2.2872345327\H,2.9321714732,1.4022416737,-2
.3855639841\\Version=IA64L-G03RevD.01\State=1-A\HF=-2385.0104382\RMSD=

Fin1

1\1\GINC-A04\Freq\RB3PW91\6-31G(d)\C31H44N6O6S1\TS\15-Nov-2007\0\\#P B
3PW91/6-31G(d) geom=check guess=read Freq=NoRaman Name=TS\\Fin for TS1
\\0,1\S,4.5403644682,0.8968184863,-1.2751374407\N,1.9292124872,1.31561
80668,-0.7659933942\N,2.4366703746,-0.7255258273,-1.6425196859\N,3.632
2350765,0.9697478622,1.982426883\N,1.8498149594,-0.4086637925,2.520494
2934\N,1.4144471145,1.7785100199,2.0334180608\C,2.1803209654,2.6397168
747,-0.2394391902\C,1.4041077707,2.8842679128,1.0726944548\C,1.8242210
007,4.2290041243,1.6805224796\C,1.544748709,5.3717673732,0.6996452653\
C,2.2122205928,5.1299603223,-0.6567220289\C,1.8461567012,3.7597171137,
-1.2349799106\C,2.8915586745,0.4779831404,-1.2252283659\C,3.3023428579
,-1.8584602042,-1.9437732165\C,2.575976235,-2.8044962904,-2.9015464951
\C,3.7693055363,-2.576765812,-0.6825568265\C,2.8520200273,-3.001448110
3,0.2858763975\C,5.1262981531,-2.841635627,-0.479944296\C,3.2815103665
,-3.6844851279,1.4233775402\C,5.5602948724,-3.5235970188,0.6568732651\
C,4.639149632,-3.9489394686,1.6122277705\C,2.2975127787,0.7873909602,2
.1442649297\H,1.282028721,4.3815989186,2.6207321639\H,0.3430339868,2.9
675949566,0.8005793646\H,3.2614362458,2.6881655551,-0.0679526287\H,0.4
572591013,5.4592325767,0.5594731147\H,1.880023422,6.3245625926,1.12715
22974\H,1.9357816667,5.9223640407,-1.3633940343\H,3.3042478307,5.18648
95898,-0.5404997664\H,0.7754332621,3.7165106166,-1.4790340568\H,2.4001
034939,3.5700198171,-2.1612046086\H,2.8949266819,4.2029450359,1.933312
938\H,0.9567751541,1.0061845897,-0.7733063731\H,1.4433897002,-0.926244
7406,-1.5333140669\H,4.1858282334,-1.4499840403,-2.4434126279\H,3.2145
801218,-3.6611609038,-3.1378687488\H,1.6488626093,-3.1831587486,-2.457
8438676\H,2.324497323,-2.2853180777,-3.8323959714\H,1.7952280877,-2.78
1113365,0.1540155613\H,5.8504411065,-2.4950486579,-1.213390068\H,2.554
0260311,-4.0191329767,2.1603732407\H,6.621145601,-3.7144752002,0.79951
23464\H,4.9750228008,-4.4810432148,2.4988454417\H,0.4673092297,1.52370
55643,2.414791461\H,0.8415383782,-0.6267761643,2.365557201\H,2.5069519
748,-1.1430773263,2.7362894519\H,4.1392443086,0.2401160548,1.491812840
6\O,-0.4152506807,-1.5581843426,-1.3007961757\C,-1.5176651806,-1.67566
07456,-0.8026944104\C,-2.5937394246,-0.6101740868,-0.6975794241\O,-1.0
294765737,1.2622439874,-0.8771570973\C,-2.1101181081,0.7622407119,-1.1
11922424\O,-3.0831837908,1.4136077778,-1.766574724\C,-2.7929477671,2.7
717749748,-2.1505827576\C,-4.0228145136,3.325241747,-2.8351973191\O,-2
.0253446852,-2.8551321982,-0.418724502\C,-1.1723102214,-4.0055048013,-
0.5396606322\C,-2.0111468758,-5.2286564704,-0.2405774043\H,-1.39435688
88,-6.1298335914,-0.3232384324\H,-2.4218575769,-5.1844931264,0.7728647
154\H,-2.8417089849,-5.3147217788,-0.9484587125\H,-0.7558884551,-4.032
3749889,-1.5517405878\H,-0.3433035944,-3.8989721735,0.1666954969\H,-3.
3658863943,-0.9134776336,-1.4140165281\H,-1.9193665273,2.7699143573,-2
.8108416404\H,-2.5295916758,3.3407774295,-1.2526814806\H,-3.8454210304
,4.3647830992,-3.1309468775\H,-4.2653882565,2.7484322235,-3.7329940783
\H,-4.8879514646,3.2986922797,-2.165815556\H,3.9320289524,1.8893871222
,1.6944233744\C,-7.0187319411,-1.3793043728,0.2983363072\C,-5.64096030
32,-1.5110835478,0.4577856539\C,-4.8137127345,-0.3871120898,0.56786674
55\C,-7.5980402679,-0.1128322627,0.2484457924\C,-6.7865569027,1.015034
9338,0.354686719\C,-5.4082995003,0.8784590689,0.5096531069\H,-5.195321

4319,-2.5028330849,0.5016328186\H,-7.6405653831,-2.2679840422,0.221908
0207\H,-8.6734687467,-0.0053879288,0.1316665328\H,-7.2280585078,2.0082
794897,0.3209787431\H,-4.7912064533,1.7688466457,0.5884493629\C,-3.316
6868863,-0.5845715115,0.730083673\C,-2.7239735292,0.3839314038,1.69256
76737\H,-3.1559352482,-1.6008328946,1.107672261\N,-1.4942938033,0.2156
694679,2.1311087232\O,-0.9867065702,1.024800997,2.9941589849\O,-0.7752
317288,-0.7646022587,1.687494691\H,-3.2650327689,1.2091597439,2.130074
2741\\Version=IA64L-G03RevD.01\State=1-A\HF=-2385.0290668\RMSD=2.638e-

Init5

1\1\GINC-A09\Freq\RB3PW91\6-31G(d)\C31H44N6O6S1\SHUBINA\21-Nov-2007\0\
\#P Geom=AllCheck Guess=Read SCRF=Check GenChk RB3PW91/6-31G(d) Freq\\
Init for TS5\\0,1\S,0.0554263464,4.7136172639,1.4317427319\N,1.1136859
544,2.237604468,1.2842764597\N,-1.030680527,2.3339408699,2.0450605049\
N,0.443931744,3.9586655056,-1.7057145974\N,-0.4771427184,2.1983100353,
-2.9042530064\N,1.3205567565,1.7891013896,-1.5242093193\C,2.3018212553
,2.7297182164,0.6242303465\C,2.5302568014,2.0018871531,-0.7190667211\C
,3.6771272064,2.6629467884,-1.4903873392\C,4.9612734332,2.6030040478,-
0.6545875938\C,4.7697715081,3.2122405063,0.7374096124\C,3.5724353907,2
.6005425228,1.4724161571\C,0.0466936569,3.0053295181,1.5915217596\C,-2
.3603631159,2.9235066675,2.1430377641\C,-3.1952255958,2.1026414674,3.1
270846485\C,-3.035930911,3.0296310974,0.77884225\C,-3.0574282455,1.938
9672301,-0.0992855155\C,-3.6539138985,4.2205880364,0.3869447035\C,-3.6
913937296,2.0406301385,-1.3383342505\C,-4.2871236344,4.325316095,-0.85
11046952\C,-4.308269186,3.2343755999,-1.7199419776\C,0.432006464,2.646
3730853,-2.0075834407\H,3.8197313221,2.147547076,-2.4479895428\H,2.822
7993955,0.9783324856,-0.4624790123\H,2.1411032006,3.7985461454,0.44965
3038\H,5.2662209316,1.5515850894,-0.550950389\H,5.7745490509,3.1127918
108,-1.1857819685\H,5.6824418853,3.0841154475,1.332437561\H,4.61237250
42,4.2961583729,0.6392533553\H,3.7471257783,1.539914825,1.6957027933\H
,3.4046409685,3.1117636209,2.426916938\H,3.418805464,3.7054374977,-1.7
200512059\H,1.0465143944,1.2128326634,1.3588572137\H,-0.9995656198,1.3
105192192,1.9738669334\H,-2.2310039455,3.9362168172,2.5351356187\H,-4.
1980079138,2.53088947,3.2217685383\H,-3.2964358581,1.0682567981,2.7824
077841\H,-2.7201367822,2.0943186395,4.113791135\H,-2.5653829287,1.0103
951429,0.18494963\H,-3.6236183436,5.0795616197,1.0535794438\H,-3.71166
55726,1.1783679628,-2.0021774521\H,-4.7600585087,5.2614221591,-1.13880
46092\H,-4.8101177827,3.3099955773,-2.682100613\H,1.1223494113,0.80771
37142,-1.7264201459\H,-0.5151654694,1.1940930324,-3.0933686754\H,-1.39
22472935,2.6302882929,-2.8600698679\H,-0.2815389684,4.5055395153,-2.15
07474346\O,-1.1116178403,-0.42267532,1.3800004948\C,-0.8635192969,-1.6
379321837,1.4592114261\C,0.3738777952,-2.309816063,1.3377519668\O,1.91
16984219,-0.4656838132,1.1382809378\C,1.6226231966,-1.6711567621,1.170
7654715\O,2.6314486364,-2.609245134,1.0156693923\C,3.9584381292,-2.098
330777,0.9344824336\C,4.9049805645,-3.2816318341,0.896331063\O,-1.8852
879881,-2.5331101525,1.665198605\C,-3.1882289546,-1.9919304918,1.84188
17127\C,-4.1466176246,-3.1567906541,1.9935223964\H,-5.1697605068,-2.79
06651479,2.1341721989\H,-4.1261381878,-3.7943078652,1.1034157169\H,-3.
8770326217,-3.7717054949,2.8582639214\H,-3.2067951843,-1.3493180366,2.
7312933085\H,-3.4578255269,-1.3653507288,0.9829085961\H,0.3684391197,-
3.3831483188,1.4718508057\H,4.1597915273,-1.455172037,1.7990375952\H,4
.0660692206,-1.4776777993,0.0358429359\H,5.9428821661,-2.9350506738,0.
839102072\H,4.7961241676,-3.8951348583,1.7966928172\H,4.7077135441,-3.
9139994405,0.0239027183\H,0.5830004171,4.2453121335,-0.7208640832\C,-1
.2301267989,-7.0506678303,-1.4192641439\C,-1.0763893881,-5.7108025488,
-1.7463623566\C,-0.0011841506,-4.9675097304,-1.224394271\C,-0.31672248
19,-7.6745439838,-0.5655576682\C,0.7532366049,-6.9508165516,-0.0402367
645\C,0.9120972645,-5.6095347003,-0.3677715466\H,-1.7904290643,-5.2413
80721,-2.4171298338\H,-2.0631279838,-7.6146085822,-1.8302047628\H,-0.4
417955313,-8.7238199044,-0.3111846513\H,1.4626090602,-7.4316775783,0.6
275835536\H,1.7329248369,-5.0329981576,0.0514500719\C,0.2320180822,-3.
5704401986,-1.5422769954\C,-0.5652117808,-2.7805109076,-2.2988136484\H
,1.1485381624,-3.1294722684,-1.1633824348\N,-0.2027133194,-1.438624205
9,-2.5403245378\O,-1.0265376564,-0.7239493813,-3.1378283829\O,0.899393
4357,-1.0194189632,-2.159398815\H,-1.5276647405,-3.0293180309,-2.72194
9863\\Version=IA64L-G03RevD.01\State=1-A\HF=-2385.0157922\RMSD=4.966e-

TS5

1\1\GINC-A06\Freq\RB3PW91\6-31G(d)\C31H44N6O6S1\SHUBINA\16-Nov-2007\0\
\#P B3PW91/6-31G(d) geom=check guess=read Freq=NoRaman Name=SHUBINA\\M
e123Nc40-e\\0,1\S,3.5327542699,3.0862094411,1.0095796105\N,2.358757893
6,0.6624382516,1.1760187309\N,1.0008941696,2.4397707314,1.6137112211\N
,3.6323882075,1.8554049231,-2.0196934401\N,1.6892935742,1.3937594902,-
3.2233782407\N,2.3637966432,-0.0687413931,-1.5849532218\C,3.5361709923
,-0.0041038193,0.666447901\C,3.2242626546,-0.7793377402,-0.634232276\C
,4.5182165254,-1.3232279411,-1.2498691309\C,5.2162172084,-2.2545417942
,-0.2519281718\C,5.4584005292,-1.5706189391,1.0972251119\C,4.173455828
3,-0.9675877371,1.6741815565\C,2.2307237083,2.0048000252,1.2706520843\
C,0.5774331989,3.8309736242,1.5188532331\C,-0.6212424732,4.0482506634,
2.4442990671\C,0.2577249778,4.2425248331,0.0858060246\C,-0.48827222,3.
4117505185,-0.7576757994\C,0.6869859295,5.4810080884,-0.400305433\C,-0
.8002025776,3.8137841136,-2.0565552895\C,0.3739235847,5.8882879799,-1.
6962497641\C,-0.3714099157,5.0548106981,-2.5301492748\C,2.5577807739,1
.0646027714,-2.2434211885\H,4.2820778912,-1.8608376737,-2.1760796234\H
,2.5983660699,-1.6284525651,-0.3391914176\H,4.2694889866,0.7836232462,
0.4672837316\H,4.5900388025,-3.1457395804,-0.0996314389\H,6.1641100148
,-2.6090223592,-0.6753299586\H,5.8915884122,-2.2843235011,1.8089175122
\H,6.201625363,-0.7705115338,0.9683670632\H,3.451218577,-1.7550468172,
1.9267103086\H,4.3876999659,-0.4148239378,2.5957907012\H,5.1808981926,
-0.4908910192,-1.5207438616\H,1.5322137737,0.0676327545,1.2966938026\H
,0.2544929228,1.7422597566,1.6538147826\H,1.4159665663,4.4406976515,1.
867616367\H,-0.9464809443,5.0921583004,2.3997436936\H,-1.4640361724,3.
4174563005,2.1424301644\H,-0.355211947,3.8055715494,3.4788222602\H,-0.
8227295099,2.4385019549,-0.4056810726\H,1.2850079595,6.1264193903,0.23
96746976\H,-1.3806604885,3.1496033386,-2.6921168731\H,0.7188994159,6.8
546469092,-2.0567568442\H,-0.6205459352,5.372295068,-3.5402407022\H,1.
4632333301,-0.534387584,-1.7670765279\H,0.807508858,0.8635813377,-3.25
60525684\H,1.5516343627,2.3850236951,-3.3741257732\H,3.6566855353,2.69
74521238,-2.5805221093\O,-1.1491221177,0.5146006328,1.3310284778\C,-1.
8864351443,-0.4417147538,1.5821786422\C,-1.6207098472,-1.8356070392,1.
3826921652\O,0.7914439662,-1.7539439396,1.2757233738\C,-0.2811871431,-
2.3558674763,1.2883396212\O,-0.3165009349,-3.7196237973,1.2041477873\C
,0.9470097889,-4.3840589647,1.1283245109\C,0.6789884192,-5.8745349106,
1.1081968384\O,-3.1552422856,-0.2522095374,2.037808846\C,-3.561605531,
1.1001748356,2.2563181946\C,-4.9876203841,1.0703474578,2.7657356021\H,
-5.3444298325,2.0905476333,2.9444241316\H,-5.6516622498,0.5925752644,2
.0385827333\H,-5.0531253986,0.5116370433,3.7047086802\H,-2.8882819697,
1.567827564,2.9839570423\H,-3.4772472727,1.6657960346,1.3208705485\H,-
2.3677838921,-2.524243323,1.7540307574\H,1.5608460323,-4.0963581709,1.
9893902813\H,1.4748468221,-4.0599041139,0.2237554738\H,1.6243382506,-6
.4256184199,1.0561441992\H,0.1472265685,-6.1873690057,2.012518349\H,0.
073024717,-6.1515265247,0.2392674797\H,3.8570431514,2.0654132542,-1.03
37589787\C,-6.1720620913,-2.3946642952,-0.1861063749\C,-4.9278634508,-
1.8502525517,-0.4847724213\C,-3.7739599052,-2.6501188962,-0.4712271734
\C,-6.2906996681,-3.746696108,0.1362885347\C,-5.1519419078,-4.55161243
73,0.1631654469\C,-3.9077142927,-4.0076841795,-0.134794785\H,-4.854647
7459,-0.7939210412,-0.7246798892\H,-7.0549124847,-1.7607112352,-0.2046
992254\H,-7.264847334,-4.1692195299,0.3683523898\H,-5.2336874121,-5.60
53681439,0.4167893644\H,-3.0176478455,-4.6311631699,-0.1003483944\C,-2
.4434504691,-2.1418004659,-0.8255535592\C,-2.2684987609,-0.9950801937,
-1.5829487407\H,-1.6509994115,-2.8792753968,-0.8816324988\N,-1.0483909
985,-0.6942001275,-2.1339889499\O,-0.9446100382,0.3549126253,-2.821892
24\O,-0.0654902211,-1.4601676224,-1.9634877205\H,-3.022401161,-0.24993
2568,-1.7867221444\\Version=IA64L-G03RevD.01\State=1-A\HF=-2385.013908
2\RMSD=6.445e-09\RMSF=3.046e-06\ZeroPoint=0.7435696\Thermal=0.7888106\
Dipole=-0.5948719,-0.6558382,-0.7448806\DipoleDeriv=-0.7884761,-0.1684
591,0.0465248,-0.2146857,-0.6673579,-0.0437278,0.079586,-0.0649885,-0.

Fin5

1\1\GINC-A09\FOpt\RB3PW91\6-31G(d)\C31H44N6O6S1\SHUBINA\20-Nov-2007\0\
\#P B3PW91/6-31G(d) Opt Freq=NoRaman Name=SHUBINA\\Fin for TS5\\0,1\S,
-0.0667119821,4.5939060387,1.3044609144\N,1.0642280622,2.1461990487,1.
338980841\N,-1.18053239,2.1982306064,1.746106406\N,0.8699271558,4.0747

247127,-1.8669758143\N,-0.0948661514,2.3109013706,-3.067707651\N,1.464
0837292,1.8403294121,-1.4645627094\C,2.3065003001,2.6449237031,0.78784
49095\C,2.6131869699,1.9940766473,-0.5809789264\C,3.8297801452,2.66800
977,-1.2250472241\C,5.0478645527,2.5248465527,-0.3058445655\C,4.768825
0342,3.0549746017,1.1043749486\C,3.5071797502,2.4360608833,1.715999472
\C,-0.0588280833,2.8940164543,1.4585697181\C,-2.5173308203,2.776245999
8,1.7657037029\C,-3.4199009749,1.9044473882,2.6424731608\C,-3.10815564
18,2.9549792527,0.3717702672\C,-2.9399265939,1.9884973266,-0.625105754
5\C,-3.876177078,4.0877726368,0.0854460931\C,-3.5336443489,2.148506243
4,-1.8774641725\C,-4.4730218856,4.2502189761,-1.1632256912\C,-4.304834
4286,3.2788039779,-2.1501183252\C,0.7403935447,2.7425469698,-2.1044437
517\H,4.0258457289,2.2041107134,-2.199030752\H,2.884533993,0.952449306
4,-0.3698376554\H,2.1677527823,3.7240698638,0.6662635219\H,5.324499369
1,1.4615751342,-0.2459904688\H,5.9094525407,3.0439145798,-0.743635913\
H,5.6320528661,2.8679432014,1.7551653002\H,4.6434646347,4.1463447036,1
.0605498055\H,3.6479227113,1.3601697385,1.8890793212\H,3.2855292723,2.
8925401569,2.6872401346\H,3.6142043714,3.7278533991,-1.4115076699\H,0.
9821090489,1.1300737516,1.3980998225\H,-1.127536474,1.1829853045,1.695
3571192\H,-2.4231936532,3.7660639487,2.2221136717\H,-4.4226743474,2.33
89197034,2.6969529451\H,-3.5145546811,0.8963972749,2.2233185372\H,-3.0
139380731,1.8277637271,3.6571389207\H,-2.3311794815,1.1065917145,-0.44
24206875\H,-3.9955499588,4.8572381799,0.8457990372\H,-3.3884041517,1.3
784394026,-2.6314795582\H,-5.0628945389,5.1407869073,-1.3675627033\H,-
4.7723938171,3.4029089009,-3.1241868716\H,1.1954790359,0.8338355617,-1
.6131757238\H,-0.2595667833,1.2822935235,-3.0910689081\H,-0.9302957546
,2.8623630195,-3.2134910491\H,0.2397203977,4.6593006809,-2.4009686066\
O,-1.0193800244,-0.7354933934,1.1936972977\C,-0.8183489195,-1.93255354
12,1.212535807\C,0.41735741,-2.6584141791,0.7317137298\O,1.8237440739,
-0.7750120533,1.4468615179\C,1.6960189388,-1.8645749574,0.9253303022\O
,2.7407103882,-2.6264916029,0.5609456477\C,4.0499434471,-2.0646072014,
0.7557043035\C,5.0614142632,-3.1657725871,0.524176508\O,-1.7219543162,
-2.8197033667,1.6516300595\C,-2.996730187,-2.2891361779,2.0623403379\C
,-3.852460705,-3.4531611721,2.5098127538\H,-4.837051404,-3.0892864505,
2.8220557398\H,-3.9911024162,-4.172389093,1.6970966196\H,-3.3928866848
,-3.9744565843,3.3551238424\H,-2.8324459389,-1.5682517907,2.8698940837
\H,-3.4398807668,-1.7517404776,1.217285872\H,0.5341321002,-3.550861139
2,1.3564157923\H,4.1118053099,-1.6561061135,1.7692923423\H,4.181235852
,-1.2383780284,0.0490775489\H,6.0746310448,-2.7725461803,0.6601457821\
H,4.9144356332,-3.9864868554,1.2335502408\H,4.981785987,-3.5659799182,
-0.4912933905\H,0.8811880924,4.3489569884,-0.8767972071\C,-1.943145999
,-6.3723857613,-0.7695076671\C,-1.5775859872,-5.0273402321,-0.80241452
04\C,-0.2322705316,-4.6487067406,-0.7496470524\C,-0.9702312606,-7.3652
322037,-0.6762617875\C,0.3742571209,-7.0020739381,-0.6178247189\C,0.73
49741825,-5.6577301532,-0.6564218\H,-2.3516387949,-4.2687779168,-0.867
8195563\H,-2.9947687525,-6.6446198347,-0.8191780275\H,-1.2570540123,-8
.4135309025,-0.6527441735\H,1.1448282815,-7.7663126585,-0.5501777804\H
,1.7859168184,-5.3788551142,-0.6132707249\C,0.231115124,-3.2026116349,
-0.7672562056\C,-0.6099250915,-2.3190427299,-1.6229425255\H,1.25136515
32,-3.1916098837,-1.166382184\N,-0.1374249903,-1.1442962622,-1.9903703
246\O,-0.8171924866,-0.3474406043,-2.7292131193\O,1.0442201032,-0.7945
53427,-1.5874024406\H,-1.5710249579,-2.5885920985,-2.0331413399\\Versi
on=IA64L-G03RevD.01\State=1-A\HF=-2385.0320677\RMSD=7.124e-09\RMSF=2.9
53e-05\Thermal=0.\Dipole=0.2378263,-0.8429541,0.5378458\PG=C01 [X(C31H
44N6O6S1)]\\@

Init2

1\1\GINC-A02\FOpt\RB3PW91\6-31G(d)\C31H44N6O6S1\TS\08-Nov-2007\0\\#P B
3PW91/6-31G(d) Opt Freq=NoRaman Name=TS\\TS2 Init\\0,1\S,-4.8519214,0.
6041913873,0.835229306\N,-2.331080475,1.4539510597,1.1930050292\N,-2.6
171753093,-0.8126649216,1.2702435903\N,-3.0162540579,1.2908655092,-2.0
599898311\N,-0.8952557875,0.6589520738,-2.7599874246\N,-1.0688030449,2
.3019817386,-1.1801203408\C,-2.6665903305,2.7845580305,0.7259192812\C,
-1.590850907,3.3007522944,-0.2503116997\C,-2.0737337385,4.595468536,-0
.9131545477\C,-2.3439432591,5.6538561295,0.1634499308\C,-3.3153531361,
5.1536924318,1.23787375\C,-2.8700532496,3.8163346468,1.840051826\C,-3.
1861879581,0.4080103498,1.1147653915\C,-3.3364945119,-2.074742439,1.11
20962332\C,-2.552443849,-3.1763467344,1.8279394566\C,-3.6063871861,-2.

4242593449,-0.3475862275\C,-2.5598939054,-2.5147309657,-1.2723057776\C
,-4.9083128489,-2.6879793526,-0.7796884442\C,-2.8126677318,-2.86341839
81,-2.5991897269\C,-5.1645143978,-3.036500682,-2.1055886782\C,-4.11752
90047,-3.1239010988,-3.0215147332\C,-1.6753833445,1.4189396891,-1.9570
059586\H,-1.3118147737,4.9491298302,-1.6174598658\H,-0.7063452701,3.53
89099977,0.3516058878\H,-3.6331085389,2.7013319159,0.2148091921\H,-1.3
888957837,5.9245298868,0.6368254111\H,-2.7304837375,6.5689788303,-0.30
16346252\H,-3.4219855527,5.9062882491,2.0286928639\H,-4.3134055988,5.0
262548134,0.7944147708\H,-1.9345743082,3.9378300992,2.4048247646\H,-3.
6252247561,3.4336441929,2.5352349066\H,-2.9843289444,4.3978683564,-1.4
952440006\H,-1.3265060756,1.2734881844,1.3079632321\H,-1.604100367,-0.
864219758,1.2298513756\H,-4.3022276367,-1.9395602394,1.608208764\H,-3.
0845125756,-4.1286845648,1.7445825433\H,-1.5583114354,-3.3086668825,1.
3855584509\H,-2.4321081995,-2.9338428388,2.889491052\H,-1.5377479947,-
2.3139248565,-0.9632163573\H,-5.7310290213,-2.5996545636,-0.0744285413
\H,-1.980644206,-2.9562676256,-3.2942770847\H,-6.1856059934,-3.2328654
102,-2.4235360518\H,-4.3151088295,-3.4001394094,-4.0545341885\H,-0.048
9908258,2.1285391591,-1.0963737951\H,0.1125040845,0.7670864405,-2.6091
929783\H,-1.2071870546,-0.2853084515,-2.9446652871\H,-3.342548737,0.52
71969915,-2.637327061\O,0.4983071807,1.4219097627,1.0938944991\C,1.678
6690477,1.05981758,1.241332959\C,2.725075985,1.0331965045,0.2981351802
\O,1.5263445191,1.6296903687,-1.7004567217\C,2.5621616229,1.305536088,
-1.0710833228\O,3.7337025684,1.1743780864,-1.7640622151\C,3.6830717588
,1.4543978692,-3.1609786013\C,5.1030178046,1.3898114812,-3.6864808698\
O,2.1018224916,0.6074636309,2.4776449578\C,1.1534349809,0.6974127717,3
.5378399181\C,1.8609060587,0.3176597707,4.8230333163\H,1.1667227417,0.
3777065935,5.6685419006\H,2.2474901644,-0.7058728228,4.7711480524\H,2.
7007970966,0.9925790638,5.0177123492\H,0.7528852716,1.7164261723,3.589
4408106\H,0.3071062446,0.0262829158,3.3423844107\H,3.7203180348,0.7840
500051,0.6423307373\H,3.2363416815,2.4412373021,-3.3268564529\H,3.0410
785013,0.7204143803,-3.6653495591\H,5.1173295288,1.59010601,-4.7636714
239\H,5.735031883,2.131910313,-3.1881098514\H,5.5385677084,0.400175328
3,-3.5134071851\H,-3.6050193397,1.4332608329,-1.2401224347\C,6.4162895
477,-2.0238015783,2.4049471748\C,5.0968165813,-1.8338696035,2.00862672
25\C,4.5799003979,-2.5132721784,0.8916799473\C,7.2358151648,-2.9033771
366,1.6992516115\C,6.7317002946,-3.5919921765,0.5928930487\C,5.4171596
311,-3.399708954,0.1906877257\H,4.4539520843,-1.1410468226,2.546070129
\H,6.8051258945,-1.4855651071,3.2649950575\H,8.2659126158,-3.056577653
2,2.0102839808\H,7.3677723483,-4.283063301,0.0463209982\H,5.0328034037
,-3.9512744031,-0.6629202824\C,3.1952892259,-2.2628870463,0.5238270531
\C,2.5920447353,-2.6349858012,-0.6181518203\H,2.5982895638,-1.69381636
97,1.2319373316\N,1.2253930075,-2.2800029229,-0.8522029121\O,0.8049601
445,-2.435363553,-2.002307521\O,0.5449057356,-1.8408391432,0.077092639
2\H,3.0395706533,-3.1228425441,-1.4726961204\\Version=IA64L-G03RevD.01
\State=1-A\HF=-2385.0160589\RMSD=4.432e-09\RMSF=6.766e-05\Thermal=0.\D
ipole=1.4257507,-0.765877,-0.2314087\PG=C01 [X(C31H44N6O6S1)]\\@

TS2

1\1\GINC-A02\Freq\RB3PW91\6-31G(d)\C31H44N6O6S1\SHUBINA\08-Nov-2007\0\
\#P B3PW91/6-31G(d) guess=read geom=check Freq=NoRaman Name=SHUBINA\\M
a2Ne1340\\0,1\S,4.8591144443,-0.2661826584,0.0235509956\N,2.373472476,
-1.1213305532,0.5220190045\N,2.7573955159,1.0827110813,1.0112171315\N,
2.2005180957,-0.595686171,-2.588008181\N,-0.0693305039,-0.2489984715,-
2.865408113\N,0.6143674451,-1.9797525042,-1.5166069526\C,2.6707383935,
-2.3953610782,-0.0988851556\C,1.435847524,-2.9662324995,-0.8145314385\
C,1.8497626805,-4.1625477528,-1.678788598\C,2.4751924748,-5.2475532808
,-0.7923236713\C,3.6293612132,-4.7086189154,0.0596238157\C,3.227292652
4,-3.4608948692,0.8533488426\C,3.25600751,-0.08536421,0.5526904269\C,3
.5684387821,2.2713205976,1.2607794424\C,2.8515107872,3.12511505,2.3119
70301\C,3.8900768872,3.0929050682,0.0190469444\C,2.8979254865,3.440129
1776,-0.9023209269\C,5.1878620327,3.5740197052,-0.1728262696\C,3.20384
45149,4.2528805368,-1.9932979552\C,5.4932224414,4.3910458061,-1.259398
9527\C,4.4998659389,4.734035161,-2.1751489315\C,0.940161711,-0.9491952
394,-2.2834273148\H,0.9711022873,-4.5551080254,-2.2041826079\H,0.75256
76727,-3.3306870141,-0.0394363293\H,3.4717114005,-2.2116399396,-0.8264
334777\H,1.6952189783,-5.6526555014,-0.1309893166\H,2.8185277825,-6.08

31361989,-1.4144553997\H,3.9896670241,-5.4896674984,0.7401729778\H,4.4
759798293,-4.4519860569,-0.5929765939\H,2.4704795841,-3.7083636631,1.6
115684614\H,4.0931028065,-3.0371723839,1.3725374115\H,2.5642064556,-3.
8338590458,-2.4460138928\H,1.4314133243,-1.0051097863,0.8971637058\H,1
.7428462605,1.2172877661,0.9988385827\H,4.5167759143,1.9132550123,1.67
47428105\H,3.4447868515,4.0164133353,2.5373905213\H,1.87240195,3.45423
42272,1.9467538299\H,2.7047715943,2.5569222481,3.2374928854\H,1.883887
3814,3.0659872231,-0.7823784551\H,5.9708316205,3.2921449213,0.52815564
36\H,2.4203093837,4.5153514446,-2.7009726349\H,6.5103298043,4.75117034
77,-1.3955847135\H,4.735348437,5.3693752781,-3.0258138512\H,-0.3575909
386,-1.9356664544,-1.1845052704\H,-0.9935896926,-0.5836655273,-2.59592
5975\H,0.0042480731,0.7622002411,-2.7372854269\H,2.3082088022,0.213474
0848,-3.183298716\O,-0.4912428666,-1.5333472991,1.1035160344\C,-1.5452
856141,-1.1457637496,1.605846032\C,-2.8268328006,-0.9795263887,0.95364
36893\O,-2.1865299993,-1.5549243838,-1.2875409659\C,-3.0351410895,-1.4
330227406,-0.3929504336\O,-4.3430406002,-1.6562517605,-0.6513204355\C,
-4.6754300151,-2.0443753478,-1.990621811\C,-6.1658670397,-2.3052017936
,-2.028201862\O,-1.6280973088,-0.7977096664,2.9130861902\C,-0.40680593
69,-0.8499460394,3.662881682\C,-0.7388331516,-0.5074829473,5.099344170
6\H,0.1707952229,-0.5344162696,5.7088088285\H,-1.1705321411,0.49586087
16,5.1720555998\H,-1.4547918125,-1.2228133792,5.5166655748\H,0.0285287
201,-1.851166527,3.5739848744\H,0.3037507028,-0.1355566173,3.232720095
8\H,-3.6912980185,-1.0131206138,1.6051980083\H,-4.0973863798,-2.934405
5042,-2.2623096927\H,-4.3869232337,-1.2442885183,-2.682286819\H,-6.469
622246,-2.5956248479,-3.0397632927\H,-6.4372563713,-3.1129273668,-1.34
12085453\H,-6.7237444597,-1.4088485086,-1.74043856\H,2.9662082745,-0.7
404425732,-1.9351526091\C,-6.3725721215,1.7625365189,2.0333790244\C,-5
.0038524582,1.5294461193,1.9546654058\C,-4.3530430439,1.4510486109,0.7
131345186\C,-7.1224022889,1.9195941416,0.8682365214\C,-6.4909266372,1.
8392889315,-0.3728314715\C,-5.1218047204,1.6052239252,-0.4504505722\H,
-4.4208511895,1.4066057507,2.8647958956\H,-6.8542240873,1.8257115047,3
.0056974814\H,-8.1917603519,2.1049302677,0.9264263875\H,-7.0675773794,
1.9639065804,-1.2859334815\H,-4.6497496374,1.5463892489,-1.4267923903\
C,-2.8925956634,1.2505197806,0.6927086428\C,-2.1170768336,1.6484166006
,-0.3928862276\H,-2.402574869,1.3104148044,1.6594253889\N,-0.759505151
6,1.8097116451,-0.2747328333\O,-0.1094201867,2.1472973449,-1.299979798
2\O,-0.1916938582,1.6437285818,0.8341375289\H,-2.4867234471,1.84564359
96,-1.3877573873\\Version=IA64L-G03RevD.01\State=1-A\HF=-2385.0082474\
RMSD=7.067e-09\RMSF=1.671e-06\ZeroPoint=0.7435171\Thermal=0.7888057\Di
pole=-2.3677175,-1.2123093,-0.3343087\DipoleDeriv=-0.9355168,0.0649055

Fin2

1\1\GINC-A05\Freq\RB3PW91\6-31G(d)\C31H44N6O6S1\TS\16-Nov-2007\0\\#P B
3PW91/6-31G(d) Freq=NoRaman Name=TS guess=read geom=check\\Fin for TS2
\\0,1\S,-4.8642221544,0.5614881934,-0.5619885643\N,-2.2509767837,0.797
2221354,-1.1320477377\N,-2.7948610387,1.1266111791,1.0567168911\N,-2.2
09513606,-2.3147761956,-0.9593466459\N,0.0092691482,-2.7121809956,-0.4
839911665\N,-0.5183700535,-1.2164894319,-2.1735603141\C,-2.5054958838,
0.3373445274,-2.4827282841\C,-1.2823583527,-0.3651776924,-3.0930223137
\C,-1.6890052378,-1.0645208708,-4.3953437619\C,-2.2262848436,-0.030097
6786,-5.3934539381\C,-3.3521586738,0.8209042034,-4.7974278048\C,-2.955
3707046,1.4384831073,-3.4533254183\C,-3.2368484357,0.8470695594,-0.181
9893154\C,-3.6577237165,1.3143064892,2.215183659\C,-2.9258319614,2.211
4287048,3.2180875159\C,-4.1186767473,0.0207763591,2.8758262733\C,-3.20
85717618,-0.982167272,3.2263681724\C,-5.4668611752,-0.1457876037,3.202
3594001\C,-3.6459366842,-2.1252887171,3.8940834532\C,-5.9035181762,-1.
2863124729,3.8739471653\C,-4.9922165126,-2.2809686928,4.2238630726\C,-
0.918918611,-2.0452201145,-1.2122293217\H,-0.8206959869,-1.586560862,-
4.8146055259\H,-0.553483292,0.4132585437,-3.3439276131\H,-3.3547772607
,-0.3610608668,-2.4374737577\H,-1.3973861689,0.6263118105,-5.696583982
1\H,-2.569432253,-0.5367771297,-6.3038309153\H,-3.642310554,1.60772942
59,-5.5043158187\H,-4.2439029346,0.1959942884,-4.6469256328\H,-2.14169
43882,2.1668522585,-3.5827431435\H,-3.8039532526,1.9627115483,-3.00344
06692\H,-2.4513337398,-1.8261589453,-4.1822970959\H,-1.2910187176,0.80
40612758,-0.7761475483\H,-1.7798625862,1.0805164569,1.2014071496\H,-4.
5506143503,1.8350793248,1.8522571807\H,-3.5653732207,2.4032617396,4.08
49670021\H,-2.009585315,1.7301655683,3.5792815239\H,-2.6581979369,3.16

94035588,2.7583737011\H,-2.1590671556,-0.8794878995,2.9577897324\H,-6.
1841902508,0.6192126247,2.9128040437\H,-2.9275310616,-2.897533444,4.16
12983577\H,-6.9578397601,-1.401224298,4.1148972115\H,-5.3296543025,-3.
1733818881,4.7460442631\H,0.456623215,-0.9282399307,-2.0998305883\H,0.
972467744,-2.4962494898,-0.7393042981\H,-0.1414517528,-2.5271297394,0.
5224698349\H,-2.3817239287,-2.9132673449,-0.1631183891\O,1.4697126973,
0.8064332732,-2.1329536582\C,2.1315687824,1.2927573846,-1.2327366944\C
,3.2026791478,0.5706869177,-0.4328163833\O,2.7757189189,-1.716725853,-
1.1534803938\C,3.5270033888,-0.7621707564,-1.0690574876\O,4.7772884674
,-0.7791343688,-1.5391002644\C,5.2115217009,-2.0070009303,-2.161203794
7\C,6.6438407037,-1.8092882482,-2.6034165684\O,2.0932120205,2.57646722
84,-0.888105614\C,1.0559810413,3.3769731292,-1.4917421262\C,1.17932775
32,4.7756560027,-0.9313159637\H,0.4031542397,5.4153970963,-1.364286417
4\H,1.0527306599,4.7700441325,0.1554442997\H,2.155631698,5.2109369402,
-1.167393861\H,1.1853975225,3.3547314881,-2.578849172\H,0.0918629278,2
.9223033459,-1.248012491\H,4.0966152493,1.1995243265,-0.4909003297\H,4
.544006501,-2.2272668922,-3.0006080475\H,5.111860313,-2.8200102256,-1.
4348543269\H,7.0127328441,-2.7236672427,-3.0799352018\H,6.7221430946,-
0.9899951927,-3.3247874096\H,7.2883686184,-1.5811717444,-1.749003127\H
,-2.9275431484,-1.615959159,-1.1166469855\C,5.9225564102,1.0243286051,
3.2918056595\C,4.7468823697,1.2432865644,2.5761706568\C,4.1106051323,0
.1976046781,1.8998239995\C,6.4840468872,-0.249890752,3.3413610036\C,5.
8600108074,-1.3011932196,2.6717607041\C,4.6848544312,-1.0785016207,1.9
57265649\H,4.3117070867,2.2401297625,2.54663904\H,6.3966432037,1.85071
79903,3.8154936026\H,7.3990186243,-0.4240372754,3.9016823468\H,6.28659
42729,-2.3006368188,2.7087873931\H,4.2047526922,-1.9109190368,1.447416
3905\C,2.8424499782,0.4682196236,1.1081456025\C,1.7698672917,-0.502471
2432,1.4635130166\H,2.4861958188,1.4767404532,1.3511323255\N,0.4948346
169,-0.2311522908,1.2760552811\O,-0.4160327541,-1.065315252,1.64001800
62\O,0.133942168,0.8783055784,0.706831487\H,1.9720299972,-1.4264797048
,1.9852748147\\Version=IA64L-G03RevD.01\State=1-A\HF=-2385.0282555\RMS

Init3

1\1\GINC-A02\Freq\RB3PW91\6-31G(d)\C31H44N6O6S1\TS\29-Nov-2007\0\\#P B
3PW91/6-31G(d) geom=check guess=read Freq=NoRaman Name=TS\\TS3 Init\\0
,1\S,4.8943226117,1.0949006936,-0.9695338973\N,2.2542204782,1.42284707
7,-1.3835908386\N,3.08997092,-0.6095894825,-1.9872117167\N,3.327595049
9,1.3186156334,1.9724648102\N,1.6481806222,0.0032691376,2.8950164402\N
,1.1000514,1.5021275681,1.2413052617\C,2.2273984231,2.6998871129,-0.69
77273002\C,1.1614901513,2.7106941025,0.4162978407\C,1.2584019951,4.004
4504546,1.2337012588\C,1.0706376634,5.2195089582,0.3187495932\C,2.0441
096095,5.2054886828,-0.8627388224\C,1.9784994758,3.8856625661,-1.63826
40224\C,3.3362989562,0.612568121,-1.4643801197\C,4.0328805902,-1.72145
29421,-1.9432548164\C,3.6554210557,-2.7332333797,-3.0276553768\C,4.098
1470756,-2.3706495546,-0.5651367601\C,2.9329472188,-2.7228995274,0.125
0092395\C,5.3351709513,-2.6484929639,0.0226764676\C,3.0055622569,-3.34
05262026,1.3729421597\C,5.4119058737,-3.2691182196,1.268732252\C,4.246
0771486,-3.6169546592,1.9494558587\C,2.0272810461,0.9403926447,2.00009
86765\H,0.4827751455,3.9837854301,2.0064469008\H,0.1772550172,2.706675
0093,-0.0631577061\H,3.2271300603,2.8405718503,-0.2754121122\H,0.03862
73209,5.2185963616,-0.0604618192\H,1.1887647982,6.1439058779,0.8975751
565\H,1.8365673253,6.0463511064,-1.5360252727\H,3.0695828364,5.3491679
915,-0.4927047978\H,0.9949261427,3.7741267395,-2.1174708106\H,2.731239
0019,3.864106379,-2.4342289358\H,2.2319187072,4.0529150467,1.740140219
3\H,1.3451367636,1.0244012927,-1.6215678185\H,2.1172274042,-0.85681339
27,-2.1713858622\H,5.0190845643,-1.3048266131,-2.1679001102\H,4.368595
5761,-3.5630732408,-3.0290380591\H,2.6572140408,-3.1506977421,-2.85333
89303\H,3.6666772839,-2.2589971375,-4.0148714157\H,1.9585065077,-2.508
2734088,-0.3079918319\H,6.2470681957,-2.3606491337,-0.4955333565\H,2.0
887928426,-3.6147487485,1.8900021931\H,6.384031414,-3.4742359734,1.710
6782411\H,4.3033772211,-4.1047034282,2.9197701679\H,0.1368174125,1.142
4452903,1.3497810914\H,0.6873776529,-0.3504708066,2.7753125932\H,2.334
5530824,-0.7072968879,3.1097623951\H,3.9423790246,0.7796078401,2.56733
29525\O,-1.6218029429,1.9884939625,1.2165465675\C,-2.7128475633,1.4075
617362,1.2873536902\C,-2.9941719265,0.0571671015,1.5992028848\O,-0.751
8323244,-0.7453790629,1.8541535028\C,-1.9929938761,-0.880042896,1.9118
435342\O,-2.5204145255,-2.0865114163,2.297220529\C,-1.5902120222,-3.10

8209071,2.6322225745\C,-2.3838411082,-4.3626806161,2.9389504886\O,-3.8
724764052,2.1172660194,1.0280087508\C,-3.7029480473,3.5133698967,0.793
6303454\C,-5.0810876525,4.1230896801,0.6322783347\H,-4.9997344305,5.20
30266508,0.4652450537\H,-5.6088986978,3.6849475019,-0.2218165695\H,-5.
6861834795,3.9574356234,1.5295581732\H,-3.1636381965,3.964137728,1.634
8367742\H,-3.0890296918,3.6715978745,-0.1019706186\H,-4.027210821,-0.2
471429859,1.7018647848\H,-0.9921721361,-2.7997631877,3.5004036959\H,-0
.8932755956,-3.268158306,1.8001690488\H,-1.7095524466,-5.1835515555,3.
2081283144\H,-3.0715725027,-4.1923455504,3.7735116594\H,-2.9739076734,
-4.6705791302,2.0695744065\H,3.7663260745,1.5198753476,1.0668868896\C,
-6.7647792543,-0.7103386498,-0.9797642801\C,-5.4319054874,-0.317103723
,-1.0062244957\C,-4.4500222746,-1.158910335,-1.5592558567\C,-7.1409072
36,-1.9443136047,-1.5098848347\C,-6.1765661621,-2.7875258434,-2.066608
5073\C,-4.8438546332,-2.4001230135,-2.0926922848\H,-5.1298844527,0.630
5090619,-0.5671503597\H,-7.5113856474,-0.0540707402,-0.5410822518\H,-8
.1837751616,-2.2501911239,-1.4918086522\H,-6.4682210035,-3.7467876589,
-2.4856477634\H,-4.1075344509,-3.0581837201,-2.545513607\C,-3.07403743
67,-0.6912018368,-1.5845549202\C,-1.9984960638,-1.4272325149,-1.937846
0171\H,-2.8984907328,0.3492597935,-1.3292920268\N,-0.7072273866,-0.844
8141568,-2.0049890229\O,0.2359309581,-1.6171296073,-2.243092847\O,-0.5
621083303,0.3717629817,-1.8525112682\H,-1.9777412834,-2.483834278,-2.1
62693816\\Version=IA64L-G03RevD.01\State=1-A\HF=-2385.0180737\RMSD=6.5
44e-09\RMSF=1.431e-05\ZeroPoint=0.7425327\Thermal=0.7894032\Dipole=-0.
852388,-0.8721899,-0.1427401\DipoleDeriv=-0.901448,-0.1149009,-0.01215
41,-0.1125449,-0.5330503,-0.0543839,-0.0467328,-0.1191563,-0.5013694,-
1.1338365,0.5896097,0.1007044,0.4066125,-1.1313934,-0.0602889,-0.03372

TS3

1\1\GINC-A05\Freq\RB3PW91\6-31G(d)\C31H44N6O6S1\SHUBINA\08-Nov-2007\0\
\#P B3PW91/6-31G(d) geom=check Freq=NoRaman Name=SHUBINA guess=read\\M
a4Na30\\0,1\S,5.0127740512,1.2179293429,-0.8209369978\N,2.3696277353,1
.5227495809,-1.2366543111\N,3.2376514672,-0.4512953565,-1.9604515058\N
,3.28302783,0.938942598,1.9955822966\N,1.5467395827,-0.4401772239,2.66
07890647\N,1.0720164427,1.3617602737,1.3071071421\C,2.3213494806,2.729
1871625,-0.4394133452\C,1.2033871833,2.6518895275,0.620546054\C,1.2999
544936,3.8462859615,1.5765102174\C,1.1795227527,5.1565597359,0.7891296
086\C,2.1946233178,5.2394703482,-0.3543899851\C,2.1321967555,4.0084042
913,-1.2643997459\C,3.4655478073,0.7379816257,-1.3644085524\C,4.182041
1331,-1.5624853417,-1.9318051067\C,3.8547390067,-2.5223455165,-3.07702
61868\C,4.1830832893,-2.2646606635,-0.5768914031\C,2.9993515782,-2.773
4231373,-0.0278261987\C,5.3731971925,-2.4125465494,0.1415826469\C,3.01
27193817,-3.4233529332,1.2065837751\C,5.3889487761,-3.0582432293,1.377
9795439\C,4.2075558106,-3.5679438473,1.9157036201\C,1.9705452555,0.629
2965667,1.9472956816\H,0.4970119729,3.7707336502,2.3178944372\H,0.2454
213999,2.7277676587,0.0966865068\H,3.2998636787,2.8219456908,0.0437379
621\H,0.1623275862,5.2255361061,0.3771329621\H,1.2989145542,6.01058573
54,1.4672650298\H,2.0285117046,6.1510046952,-0.9416845639\H,3.20838098
98,5.319814837,0.0638067394\H,1.1684829116,3.9677893905,-1.7921849579\
H,2.9181540539,4.0485962626,-2.0264795197\H,2.2546158655,3.8106071569,
2.1190981065\H,1.4617378526,1.1300945881,-1.5158616396\H,2.2563542847,
-0.7088901812,-2.1192162997\H,5.1763220481,-1.1358857441,-2.0921060498
\H,4.561188045,-3.3582917356,-3.0813766973\H,2.8441491968,-2.933743535
5,-2.9774515844\H,3.9205042379,-2.0012702291,-4.0378805364\H,2.0580879
123,-2.650065702,-0.5597475945\H,6.2930872143,-2.0010192767,-0.2663103
165\H,2.0878170224,-3.8330997949,1.6080167583\H,6.3252784744,-3.161932
3649,1.9211766936\H,4.2189470017,-4.0836173901,2.873265195\H,0.1047818
476,1.0159204817,1.3249534078\H,0.5756872649,-0.7189973558,2.506045004
\H,2.1949662366,-1.2162540667,2.7127388342\H,3.8693728459,0.2586395207
,2.4604739597\O,-1.7064537479,1.9216521392,1.1896282617\C,-2.765985611
5,1.3967765528,0.8621286942\C,-3.0929481251,-0.0126300968,0.804347634\
O,-1.0083638434,-0.863136501,1.6096410334\C,-2.2203268387,-0.977559134
5,1.4113574366\O,-2.8663075829,-2.1383519689,1.6831615391\C,-2.0717254
828,-3.1947305556,2.2331088828\C,-2.9799597886,-4.3879739366,2.4416558
109\O,-3.8296919599,2.147487737,0.4643180187\C,-3.6369327272,3.5670828
743,0.4847368887\C,-4.9548416865,4.2128366349,0.1126646415\H,-4.853003
4582,5.3034973971,0.1215518711\H,-5.2742561734,3.9069360114,-0.8889042
214\H,-5.7395784964,3.933955406,0.8230565598\H,-3.304126237,3.87127155

91,1.483048949\H,-2.8414989379,3.8350317564,-0.2204693987\H,-4.1488740
543,-0.2485502187,0.8305740468\H,-1.6297286826,-2.8594162995,3.1789798
601\H,-1.248223178,-3.4241772254,1.5472797522\H,-2.4114803713,-5.22231
2697,2.8666951486\H,-3.7958950823,-4.1407575114,3.1278990372\H,-3.4180
222122,-4.7171823194,1.4941504008\H,3.7513076785,1.3275801948,1.169123
1025\C,-6.499256877,-0.6635001252,-2.250290215\C,-5.221132345,-0.18525
28262,-1.9800666202\C,-4.1874183197,-1.0586092863,-1.608535389\C,-6.77
23320526,-2.0265574026,-2.1454740951\C,-5.7572699071,-2.904413581,-1.7
641821366\C,-4.4784855829,-2.4271065841,-1.4976613977\H,-5.0136202399,
0.8792523451,-2.0481215409\H,-7.2837045186,0.0297813629,-2.5428956208\
H,-7.7700703768,-2.4030642451,-2.3556624312\H,-5.9638113677,-3.9678518
341,-1.6733670162\H,-3.704220942,-3.1218479431,-1.186996497\C,-2.84028
6319,-0.5058751985,-1.3729672441\C,-1.7013100955,-1.2734295177,-1.6091
808293\H,-2.7276018811,0.5540821026,-1.5753508587\N,-0.4730872243,-0.6
917264599,-1.7830376093\O,0.5055689291,-1.443586088,-2.0424334391\O,-0
.3365978916,0.5560446035,-1.7149775317\H,-1.6778489146,-2.3501587337,-
1.6829766275\\Version=IA64L-G03RevD.01\State=1-A\HF=-2385.0146081\RMSD
=1.738e-09\RMSF=6.845e-06\ZeroPoint=0.7437794\Thermal=0.7890701\Dipole
=-1.2567748,-0.5650594,1.4096501\DipoleDeriv=-0.9290526,-0.112011,-0.0

Fin3

1\1\GINC-A08\Freq\RB3PW91\6-31G(d)\C31H44N6O6S1\TS\21-Nov-2007\0\\#P B
3PW91/6-31G(d) geom=check Freq=NoRaman Name=TS guess=read\\Fin for TS3
\\0,1\S,-4.7980227233,1.1581046972,1.2201289503\N,-2.1267198571,1.5110
642059,1.4059983482\N,-2.8741854512,-0.5246904525,2.0724041452\N,-3.52
3114261,1.0774928984,-1.8020642209\N,-1.959259696,-0.4362986756,-2.609
6673417\N,-1.2443249985,1.3110931015,-1.2888747005\C,-2.1724245891,2.7
175039444,0.6112330006\C,-1.224607801,2.6081313151,-0.604400957\C,-1.4
180474705,3.8038739997,-1.541344299\C,-1.1294457911,5.1040866093,-0.78
22421449\C,-1.9607110278,5.2231128621,0.4993651255\C,-1.816984578,3.98
7009719,1.3938691816\C,-3.1888119008,0.6874278835,1.5810130952\C,-3.79
97354967,-1.6483301849,2.1222573864\C,-3.3455434427,-2.6199142058,3.21
38593215\C,-3.9185192029,-2.3333726207,0.7642625698\C,-2.7814011868,-2
.8148472216,0.102439117\C,-5.1680856251,-2.4941000244,0.1581229388\C,-
2.9019511032,-3.4554357828,-1.1312222043\C,-5.2893437228,-3.1271163887
,-1.0790626278\C,-4.1552611414,-3.6137115167,-1.7289495932\C,-2.242350
3724,0.6585940306,-1.8608738832\H,-0.7412393536,3.7011918662,-2.396944
0723\H,-0.2006129419,2.6476118133,-0.2197899088\H,-3.2081384775,2.8379
337082,0.2749585112\H,-0.0606253485,5.1310484843,-0.5235014146\H,-1.31
2197454,5.9656434045,-1.4363244652\H,-1.6728638164,6.125337867,1.05327
34327\H,-3.020470065,5.3469551512,0.2336553671\H,-0.7901647667,3.90648
68546,1.7792917688\H,-2.4815219606,4.0564263007,2.2620347886\H,-2.4444
014857,3.8094122967,-1.9317199028\H,-1.1870973559,1.0809080997,1.48434
75353\H,-1.8678598897,-0.7716336819,2.1053229471\H,-4.7814436623,-1.24
2718487,2.3835343351\H,-4.0251741607,-3.4767328688,3.2641498164\H,-2.3
353580616,-2.9945002948,3.0164252472\H,-3.3400333818,-2.1173083219,4.1
866556747\H,-1.7983527747,-2.6695916535,0.5490113854\H,-6.052344967,-2
.1031951718,0.6554629293\H,-2.0133833672,-3.8464344408,-1.6235680313\H
,-6.2704626568,-3.2413085635,-1.534210941\H,-4.2474430338,-4.122338064
5,-2.6860811971\H,-0.3498136969,0.8183280382,-1.2659014154\H,-0.989645
7981,-0.7428412621,-2.590176093\H,-2.6262938651,-1.1972615128,-2.54303
64773\H,-4.1828774637,0.4896384159,-2.2946637949\O,1.7513518266,1.7987
910626,-1.8377281693\C,2.7065918951,1.3230075663,-1.2632772369\C,2.957
0932634,-0.1330664145,-0.9245055904\O,0.8060976919,-0.8611696844,-1.83
19565153\C,1.975624187,-1.0779060102,-1.5795130005\O,2.5692452844,-2.2
452136393,-1.8579439895\C,1.7217580483,-3.2916385845,-2.3733698304\C,2
.5689620423,-4.5349848722,-2.5252601758\O,3.7496772003,2.0534099455,-0
.8347558271\C,3.6677485032,3.4725256803,-1.0670059936\C,4.9542182065,4
.0902969534,-0.5660248058\H,4.9314739686,5.1737222417,-0.7248256193\H,
5.0893461623,3.9011318322,0.5033022559\H,5.8179949059,3.6817979812,-1.
0997097582\H,3.5159285598,3.6455924147,-2.1374702312\H,2.7913646987,3.
8646948452,-0.5400810567\H,3.9531805457,-0.3774023099,-1.3050224735\H,
1.3037554907,-2.9623164063,-3.330700533\H,0.8941796907,-3.4402848329,-
1.6726219555\H,1.9584712688,-5.3505896253,-2.9269884442\H,3.403369092,
-4.3605087191,-3.2117019597\H,2.9727991732,-4.854036873,-1.5595667828\
H,-3.9004370353,1.3867809482,-0.8918446909\C,6.2114114258,-0.622839482
1,2.6893142171\C,4.9827617018,-0.1826432191,2.2012379066\C,4.353076220

5,-0.8388996263,1.1387726225\C,6.8334515822,-1.7326291737,2.1203586615
\C,6.215008082,-2.3966633865,1.0624439231\C,4.9858660313,-1.9540441548
,0.5769109626\H,4.5012446899,0.6823972913,2.6520918128\H,6.6840564994,
-0.0949576741,3.5140807029\H,7.7930710287,-2.0767270644,2.4977989855\H
,6.6904777476,-3.2649007531,0.6121219681\H,4.5138284799,-2.4805224632,
-0.2488771395\C,2.9945627249,-0.3568122495,0.6649970272\C,1.9119402037
,-1.2486592032,1.1768693922\H,2.8163414665,0.6326063816,1.0982159687\N
,0.7060837001,-0.7463811948,1.3941284659\O,-0.2158753626,-1.4603623547
,1.9255486206\O,0.4587978534,0.4776104608,1.0649935903\H,2.077228282,-
2.2638941479,1.5068596316\\Version=IA64L-G03RevD.01\State=1-A\HF=-2385
.0294663\RMSD=4.652e-09\RMSF=6.664e-06\ZeroPoint=0.7456702\Thermal=0.7
908941\Dipole=1.6003362,-0.2265709,-2.5674293\DipoleDeriv=-0.9220337,0

Init6

1\1\GINC-A02\FOpt\RB3PW91\6-31G(d)\C31H44N6O6S1\SHUBINA\22-Nov-2007\0\
\#P B3PW91/6-31G(d) OPT Freq=NoRaman Name=SHUBINA\\Init for TS6\\0,1\S
,-0.8713459303,4.9960800406,-0.1385299232\N,-1.2330772409,2.7816848968
,1.3480658688\N,-2.0084472068,2.6537682257,-0.7892468813\N,2.056044965
9,3.4097790703,0.2831731231\N,2.6726649151,1.2380868906,-0.2646570334\
N,1.3489394112,1.5699137762,1.5747424275\C,-0.3640554531,3.2552085524,
2.4034491109\C,0.732604592,2.2167157261,2.7315967048\C,1.7390712104,2.
8144928591,3.7241347686\C,1.022422513,3.2415320656,5.0097667691\C,-0.1
405407318,4.1969438971,4.7277710546\C,-1.1113165886,3.6239458321,3.690
5715759\C,-1.3907761923,3.4013942772,0.1510951835\C,-2.1240784883,3.03
34380182,-2.1925348857\C,-3.2577927901,2.2202710659,-2.823197499\C,-0.
8186254023,2.8578634336,-2.9610863505\C,-0.0189538034,1.7232377625,-2.
7897644393\C,-0.4211236941,3.8229581506,-3.8908707125\C,1.1501683346,1
.5538093457,-3.5327831811\C,0.7445349139,3.6585520248,-4.6375333272\C,
1.5344551987,2.5230468152,-4.4604520865\C,1.9980036337,2.0793129107,0.
5286809029\H,2.5141645581,2.0707277506,3.942953072\H,0.2299183151,1.38
0600086,3.2363175557\H,0.0909177505,4.1793160671,2.0319275989\H,0.6395
236026,2.3444362076,5.518470743\H,1.7387941577,3.7059718917,5.69831498
68\H,-0.6757812701,4.4259829396,5.6573148624\H,0.2559443888,5.15183026
17,4.3538966496\H,-1.6187286793,2.7338163635,4.0909022626\H,-1.8894259
153,4.3537584098,3.4414846281\H,2.2407963175,3.6748951178,3.261638254\
H,-1.5677596179,1.8207846353,1.4375145078\H,-2.1140162099,1.6603884296
,-0.5854169258\H,-2.3881141229,4.0950758046,-2.2100545835\H,-3.3827380
448,2.5028492968,-3.87270645\H,-3.0374822713,1.1466532305,-2.791697921
9\H,-4.2010579035,2.3996658499,-2.2958720939\H,-0.2935783406,0.9595352
365,-2.0663259153\H,-1.0235946509,4.719865117,-4.0196620165\H,1.742059
8872,0.6544454887,-3.3768592921\H,1.039560106,4.4237751558,-5.35168576
8\H,2.4432462053,2.3934524575,-5.0437125159\H,1.3960882182,0.542410430
1,1.6088260043\H,2.5303534794,0.2236607688,-0.1443335819\H,2.793261909
4,1.5107089304,-1.2304223524\H,2.5108592201,3.6604746254,-0.5850106158
\O,1.9344250246,-1.0722970413,1.2481411068\C,1.6211402785,-2.261978833
3,1.0245228825\C,1.476472763,-2.9134617791,-0.212412285\O,2.1079191721
,-1.1260003773,-1.6865774736\C,1.7276458786,-2.2796467795,-1.459233080
7\O,1.4826899998,-3.1437907982,-2.5077471053\C,1.7732610806,-2.6411529
786,-3.8100523109\C,1.5915214278,-3.7791672159,-4.7943327477\O,1.36245
83005,-3.092942637,2.0837601031\C,1.551805315,-2.5408024111,3.38274264
49\C,1.3960681203,-3.6685581293,4.383227801\H,0.404355652,-4.126414799
,4.3056622493\H,2.1442631481,-4.4479078864,4.2068775796\H,1.5232450793
,-3.290815851,5.403936236\H,2.5434861437,-2.0789277839,3.4500982804\H,
0.8124828197,-1.7485302157,3.5635910844\H,1.2719390745,-3.9750263507,-
0.2050926738\H,2.7982043525,-2.253145983,-3.8337646611\H,1.104781679,-
1.8032184877,-4.0445642649\H,1.8132530841,-3.437589911,-5.8115798434\H
,2.2625924868,-4.610707498,-4.5554585139\H,0.5622322779,-4.1537690219,
-4.7780359207\H,1.213664673,3.9741143533,0.4311426109\C,-1.1975696467,
-6.5879973885,-1.0909165314\C,-1.1424774599,-5.1998804731,-1.061245029
5\C,-1.6471536436,-4.485916903,0.0408174231\C,-1.7639391431,-7.2875749
816,-0.0252971411\C,-2.2731060696,-6.5916014904,1.0731589059\C,-2.2165
841056,-5.204826361,1.1077190399\H,-0.6718894487,-4.652595453,-1.87394
87238\H,-0.7945913687,-7.1255958144,-1.9447772334\H,-1.8100951319,-8.3
732317589,-0.0491171767\H,-2.7192206098,-7.1341834783,1.9022753902\H,-
2.633051828,-4.6764936901,1.9605164778\C,-1.5930017992,-3.0340569013,0
.0071380417\C,-1.9121326819,-2.2074403209,1.0301622359\H,-1.3281997177
,-2.5709667671,-0.9380091142\N,-1.9069722796,-0.8075336007,0.848333687

\O,-2.0527028042,-0.1042839378,1.8636391391\O,-1.7755655792,-0.3281553
687,-0.2870295434\H,-2.1244017701,-2.4890130847,2.0511561931\\Version=
IA64L-G03RevD.01\State=1-A\HF=-2385.0173925\RMSD=4.803e-09\RMSF=6.558e
-06\Thermal=0.\Dipole=-0.4739882,-1.1285255,0.6938831\PG=C01 [X(C31H44
N6O6S1)]\\@

TS6

1\1\GINC-A04\Freq\RB3PW91\6-31G(d)\C31H44N6O6S1\SHUBINA\21-Nov-2007\0\
\#P Geom=AllCheck Guess=Read SCRF=Check GenChk RB3PW91/6-31G(d) Freq\\
Ma4Na30-e anti TS3\\0,1\S,-5.0910050866,0.4551496867,-0.710974491\N,-2
.9341702077,-1.0005429549,-1.4031852113\N,-2.7400405951,1.2327943066,-
1.765754562\N,-3.3392782253,-0.4306683337,1.9855120612\N,-1.1205546541
,0.0110631583,2.4976673127\N,-1.5916723541,-1.6781649393,1.0184760097\
C,-3.4013842429,-2.171530499,-0.6991062551\C,-2.3167536776,-2.70490555
68,0.2656372474\C,-2.89792654,-3.8230524459,1.1390807147\C,-3.40848759
31,-4.9663880548,0.2542945771\C,-4.4065729333,-4.4769947909,-0.7996776
901\C,-3.8456336509,-3.3125961729,-1.6223554826\C,-3.5146851261,0.2206
249205,-1.3200630609\C,-3.087888871,2.6427618417,-1.6482513821\C,-2.28
12470307,3.4325997411,-2.6823488875\C,-2.8713591915,3.1856913789,-0.23
95332263\C,-1.6838317034,2.9383507869,0.4587254847\C,-3.8540559057,3.9
716900105,0.3684644131\C,-1.4852511411,3.4685893784,1.7346992316\C,-3.
6593241892,4.5043279366,1.6424262055\C,-2.4734658391,4.2540357878,2.33
11321948\C,-2.0264112204,-0.6933788776,1.8010072873\H,-2.1262659779,-4
.1854566836,1.8287632512\H,-1.5295688409,-3.1403805408,-0.3626233464\H
,-4.2911510974,-1.8650225162,-0.1382405733\H,-2.5501719595,-5.43516512
99,-0.2493400585\H,-3.8643937951,-5.7443106736,0.8788838274\H,-4.69086
99013,-5.3039126367,-1.4618116986\H,-5.3290574071,-4.1472698978,-0.300
4765108\H,-2.9933951955,-3.6463157634,-2.2317114189\H,-4.6029280083,-2
.9234062243,-2.3114335488\H,-3.7142614361,-3.4200278911,1.7531808186\H
,-1.9743284716,-1.0538896984,-1.7705844507\H,-1.7461305554,1.019531861
4,-1.8934511457\H,-4.1537224616,2.724936571,-1.8815857173\H,-2.5367239
641,4.4952513134,-2.6274989986\H,-1.2047368049,3.3362023527,-2.4993466
131\H,-2.4950762281,3.0700901524,-3.6934576626\H,-0.9069087486,2.31755
41491,0.0184943477\H,-4.7899761799,4.1528000943,-0.1554419183\H,-0.547
2733657,3.2670375597,2.2474840464\H,-4.4396351202,5.108324451,2.099409
114\H,-2.3186027689,4.6702863564,3.3239050027\H,-0.5736154994,-1.72770
73696,0.9621715293\H,-0.1276687705,-0.0891900337,2.2585942049\H,-1.369
215735,0.9646002205,2.7269089632\H,-3.5319509516,0.3918329171,2.542518
0342\O,1.2012461567,-1.4877769372,1.4339545874\C,2.3971477593,-1.26053
11779,1.2267059916\C,3.0116330351,0.0099030972,0.9628218512\O,1.287412
1895,1.3865517876,1.9382872314\C,2.3904418796,1.2367684487,1.421912367
2\O,3.2160082648,2.2952016513,1.1965383154\C,2.7372159741,3.5730335567
,1.6342369306\C,3.8302211074,4.5870678634,1.3706331344\O,3.2879167592,
-2.2773504468,1.1384883681\C,2.7637865844,-3.5971413301,1.3331409421\C
,3.9306319996,-4.5606986443,1.2940420043\H,4.4510931638,-4.5090846835,
0.3324078698\H,4.651178122,-4.3311684534,2.0852714642\H,3.5742310271,-
5.5863936948,1.4382909278\H,2.2370614119,-3.6374984524,2.2928538566\H,
2.0306515047,-3.8152268648,0.5477030305\H,4.0935576815,0.0167249885,0.
9429835893\H,2.4876949961,3.5172528465,2.699804386\H,1.8169665681,3.82
11402089,1.0936224866\H,3.5060758364,5.5796120706,1.7017973631\H,4.745
2611732,4.3245212312,1.911038268\H,4.0653771937,4.6436858252,0.3027893
679\H,-3.9726280303,-0.4765025981,1.1798958196\C,6.4071814971,1.010841
1574,-2.0029199859\C,5.0585346092,1.0907912837,-1.6709690709\C,4.26394
44592,-0.0639191299,-1.5976192544\C,6.991875579,-0.2283439004,-2.26010
6436\C,6.2164941443,-1.3852382739,-2.1788089018\C,4.8672572438,-1.3053
609577,-1.8515635123\H,4.6076173248,2.0556639957,-1.4553452053\H,7.002
8170188,1.918314714,-2.0604636819\H,8.045419941,-0.2935553999,-2.51935
48917\H,6.6661420312,-2.3559712315,-2.3718549572\H,4.2823986879,-2.216
8384328,-1.7798839515\C,2.8304363721,0.0779669248,-1.2822331195\C,1.89
87279288,-0.8518181952,-1.7404176673\H,2.4658932257,1.0972323033,-1.20
87463243\N,0.5653791772,-0.5503154708,-1.815258686\O,-0.2221843546,-1.
4527053584,-2.2140044946\O,0.1429093713,0.5966767947,-1.5184801028\H,2
.1219026091,-1.8646703639,-2.0400918785\\Version=IA64L-G03RevD.01\Stat
e=1-A\HF=-2385.0139963\RMSD=3.596e-09\RMSF=1.707e-06\ZeroPoint=0.74352
81\Thermal=0.7888435\Dipole=1.3456053,-0.6203456,1.0395684\DipoleDeriv

Fin6

1\1\GINC-A06\FOpt\RB3PW91\6-31G(d)\C31H44N6O6S1\SHUBINA\22-Nov-2007\0\
\#P B3PW91/6-31G(d) OPT Freq=NoRaman Name=SHUBINA\\Fint for TS6\\0,1\S
,0.2451570301,4.651510714,1.8819207318\N,-0.3451434244,2.1106461497,2.
5609995613\N,-1.2606065682,2.8104062546,0.6081756998\N,2.7673655575,2.
5827569756,1.0669743359\N,2.6425723266,0.7516717416,-0.3539918812\N,1.
8650592159,0.5372319058,1.7953643631\C,0.7445003509,2.0369121792,3.505
2488823\C,1.6172597417,0.794945358,3.2167850054\C,2.8847625943,0.82819
87688,4.0761401011\C,2.5028248833,0.856218233,5.5616231872\C,1.5380708
481,2.0011543285,5.8891908709\C,0.3086236786,1.9988516638,4.9738465745
\C,-0.4884839509,3.1210707796,1.670988907\C,-1.5076635009,3.7044674446
,-0.5126797436\C,-2.8309224058,3.3075171484,-1.1730648515\C,-0.3628528
752,3.7169467965,-1.5210817605\C,0.1471382983,2.525423712,-2.051485268
2\C,0.1822158165,4.9288276076,-1.9534384088\C,1.1789051555,2.551259868
7,-2.9918968321\C,1.2108626016,4.9583985909,-2.8952013529\C,1.71482283
48,3.7687220136,-3.4174962526\C,2.3909558272,1.3036423589,0.8457141555
\H,3.5024709383,-0.0505091676,3.8543190382\H,1.0220259256,-0.077207989
8,3.5124531739\H,1.3353070979,2.9518950912,3.3823436177\H,2.0299950046
,-0.1015646966,5.824071565\H,3.408487388,0.9336115968,6.1758631616\H,1
.2274139315,1.9407510422,6.939499441\H,2.0628928054,2.9605868784,5.775
273449\H,-0.3066820697,1.1058229229,5.1543853538\H,-0.3232889993,2.871
5422466,5.1708765284\H,3.4810814461,1.7136414858,3.8188413664\H,-0.787
5121861,1.2000758902,2.3312336331\H,-1.3563827525,1.8000802514,0.42391
43996\H,-1.5979423208,4.7123257993,-0.0966992723\H,-3.0513501213,3.972
0190635,-2.0145658247\H,-2.7860699644,2.2811987704,-1.5559216856\H,-3.
6501789969,3.3688446852,-0.4491577382\H,-0.2478615024,1.5690621692,-1.
7162077832\H,-0.1916897866,5.8589165576,-1.5317516455\H,1.5565541298,1
.6132719226,-3.3948176397\H,1.6235362668,5.9127644803,-3.2135308454\H,
2.5163736265,3.7883182833,-4.1522308528\H,1.4479362646,-0.3152065257,1
.4344989854\H,2.2960857587,-0.1919511658,-0.5373242423\H,2.5780396061,
1.3655099078,-1.1564417863\H,3.144902766,3.05765841,0.2570350655\O,1.6
353798512,-1.9879903669,0.280204227\C,0.9429120377,-2.8612993705,-0.20
73039695\C,0.0625984413,-2.7621411433,-1.4334901182\O,1.5328932603,-1.
0626857532,-2.4220615523\C,0.5980129976,-1.832802996,-2.5024054826\O,-
0.1304423798,-1.9960181152,-3.6171773846\C,0.2299950854,-1.1837206919,
-4.7518248923\C,-0.5950211247,-1.6561810264,-5.9283236057\O,0.85652090
25,-4.0881749881,0.3209196291\C,1.5381978446,-4.2910264076,1.577149585
3\C,1.2846945346,-5.7174931292,2.0097938725\H,0.2152702932,-5.89988399
17,2.1540058229\H,1.6620661216,-6.4272131219,1.2668899012\H,1.79530905
66,-5.9103651038,2.9591890206\H,2.603647486,-4.0860776353,1.4326091671
\H,1.1490851831,-3.5665581929,2.2996416354\H,0.0057620271,-3.758321458
2,-1.8810284341\H,1.3045547862,-1.2887509424,-4.9324033216\H,0.0307025
955,-0.1346914272,-4.508667404\H,-0.3580734319,-1.0538335074,-6.811746
6521\H,-0.3832998583,-2.7049438063,-6.1586771206\H,-1.6649488169,-1.55
82009782,-5.7210297989\H,2.1305264235,3.196103716,1.5910863758\C,-4.50
85204198,-3.9941222307,-2.5530841363\C,-3.5512484958,-3.0494307866,-2.
1878271662\C,-2.4403000035,-3.4111621044,-1.4198276364\C,-4.3695191048
,-5.3216537503,-2.1534115884\C,-3.2672421656,-5.6945440956,-1.38546805
27\C,-2.3118204126,-4.7476691102,-1.0222279715\H,-3.6674258841,-2.0145
214081,-2.5020185734\H,-5.3629593042,-3.6913383795,-3.1535273339\H,-5.
1138023896,-6.0607715916,-2.4387729808\H,-3.1498771087,-6.727655315,-1
.0669727334\H,-1.4540820696,-5.049743187,-0.4258633437\C,-1.4343530441
,-2.3531654178,-1.008223589\C,-1.5573527897,-2.0285170464,0.4439141421
\H,-1.6577074924,-1.4339574157,-1.5573231383\N,-1.2249432076,-0.822990
8756,0.8750717035\O,-1.3007423875,-0.5211649374,2.1194676664\O,-0.7871
867335,0.0651181982,0.0423365478\H,-1.9292285951,-2.7204986115,1.18569
3197\\Version=IA64L-G03RevD.01\State=1-A\HF=-2385.0293488\RMSD=5.772e-
09\RMSF=9.400e-06\Thermal=0.\Dipole=1.4939299,-2.0671573,-1.2485828\PG
=C01 [X(C31H44N6O6S1)]\\@

Init7

1\1\GINC-A07\FOpt\RB3PW91\6-31G(d)\C31H44N6O6S1\TS\26-Jul-2008\0\\#P B
3PW91/6-31G(d) Opt Freq=NoRaman Name=TS\\Init TS 7\\0,1\S,-5.054148417
2,0.4726103552,-0.7534851146\N,-2.6661345266,-0.6904157325,-1.27620146
61\N,-2.6958523706,1.5665265078,-1.4547421357\N,-1.1184674521,-0.63764
60311,1.9264917693\N,-3.034296683,0.6548111965,2.0646980103\N,-3.20961
85303,-1.5459973692,1.3913500499\C,-3.1658076544,-2.0244183513,-1.0317

896552\C,-2.7977535626,-2.5274667169,0.384924878\C,-3.4347042311,-3.89
27997646,0.6467254582\C,-2.9867549788,-4.9066547841,-0.4106092036\C,-3
.2777026601,-4.411186411,-1.8300067091\C,-2.6746312506,-3.025510351,-2
.0822643944\C,-3.3989405334,0.4489799249,-1.1864102345\C,-3.1966122673
,2.9291251452,-1.3646566073\C,-2.5335175206,3.7701015876,-2.460695902\
C,-2.9843491888,3.554084456,0.008573534\C,-1.7871721767,3.3755818224,0
.7133392841\C,-3.9801469252,4.3595893756,0.568458088\C,-1.5958216442,3
.9898030826,1.9516234066\C,-3.7880091109,4.980375912,1.8026896733\C,-2
.5946692348,4.7969264636,2.4989716352\C,-2.4243962685,-0.5062341984,1.
7587964666\H,-3.1648832679,-4.232686788,1.6534403158\H,-1.7102868385,-
2.6433534844,0.4454015723\H,-4.2586212027,-1.9525098297,-1.0899270574\
H,-1.9083498042,-5.0810010815,-0.3005624555\H,-3.483641147,-5.86854092
96,-0.2343376865\H,-2.8885012695,-5.1256998394,-2.5655361169\H,-4.3653
177179,-4.3631847797,-1.9859907268\H,-1.5773534872,-3.086437073,-2.047
6817564\H,-2.9461936847,-2.6580342063,-3.0784881008\H,-4.529948181,-3.
7887470224,0.6266686248\H,-1.6515756367,-0.5548989089,-1.3453736423\H,
-1.6800000595,1.4386290879,-1.5088613021\H,-4.2727320637,2.8736012518,
-1.5531694885\H,-2.9116083992,4.7967935979,-2.4294446765\H,-1.44679970
29,3.8110060355,-2.3209575528\H,-2.7428172168,3.3458473997,-3.44836335
16\H,-1.0071904654,2.7252090678,0.32358317\H,-4.9206019884,4.493287902
3,0.0374514157\H,-0.6621613049,3.8221929765,2.4822875306\H,-4.57735673
19,5.5979482357,2.224558884\H,-2.4455109739,5.2751861258,3.4640021634\
H,-4.1893264818,-1.2849075274,1.3089738237\H,-3.854113354,0.8951236093
,1.5054939212\H,-2.4432553778,1.4439509895,2.2987014163\H,-0.487193247
8,0.1799540673,1.8351847181\O,0.7931251276,1.3823338188,1.8711457275\C
,1.845192141,1.3269945785,1.2136260577\C,2.0692525726,0.9834466728,-0.
143674946\O,-0.1810017291,0.5589248839,-0.7586405828\C,1.0362178687,0.
6414232732,-1.0354292292\O,1.3190115645,0.3406784314,-2.3446046448\C,2
.6348100796,0.4452608356,-2.8801390626\C,2.994216314,1.8733635654,-3.2
588068915\O,3.0340900953,1.6374940481,1.843493639\C,2.9279722308,2.039
3511387,3.2094961958\C,4.3275888242,2.3240785317,3.7153180381\H,4.2900
664536,2.6553140344,4.7590087913\H,4.9552060733,1.4276697871,3.6655051
706\H,4.8043306475,3.111233331,3.1219153601\H,2.2920769001,2.929527955
7,3.2812726451\H,2.4411607554,1.2491074663,3.7927886517\H,3.0904735646
,1.0692709668,-0.4867742544\H,2.6163510525,-0.1887587853,-3.7730391133
\H,3.3687369389,0.0134293068,-2.1888750492\H,3.9846859651,1.9049512706
,-3.727789559\H,2.2635665469,2.2711710397,-3.9706898748\H,3.0073348225
,2.5248172275,-2.379790463\H,-0.6748359771,-1.5302956448,1.7441968362\
C,6.8349012971,-0.5011508949,0.4013536996\C,5.4940850119,-0.7834834839
,0.6420559135\C,4.9383380457,-2.0084441038,0.2311486994\C,7.6415487205
,-1.4384820402,-0.2419332304\C,7.1031713286,-2.6629462727,-0.646701116
1\C,5.7647246737,-2.9471238281,-0.4139871918\H,4.8591787885,-0.0500211
591,1.1342000161\H,7.2500643018,0.4517441609,0.7178029156\H,8.69033478
62,-1.2202142515,-0.4256766677\H,7.733162071,-3.3977709361,-1.14060387
29\H,5.3617737276,-3.9089991177,-0.7191045189\C,3.5292010653,-2.245527
6086,0.5042413997\C,2.7782674165,-3.2463472517,0.0129438627\H,3.026925
6434,-1.528086797,1.1480472446\N,1.3909271046,-3.3453824522,0.35898397
28\O,0.7171346665,-4.1445015135,-0.2897034992\O,0.9468356652,-2.643969
2953,1.2723329874\H,3.072363647,-4.0026358841,-0.7014685293\\Version=I
A64L-G03RevD.01\State=1-A\HF=-2385.0233192\RMSD=6.751e-09\RMSF=
1.129e-06\Thermal=0.\Dipole=2.5808807,-0.9125471,0.1865447\PG=C01 [X(C31H44N6
O6S1)]\\@

TS7

1\1\GINC-A03\FTS\RB3PW91\6-31G(d)\C31H44N6O6S1\TS\29-Nov-2007\0\\#P B3
PW91/6-31G(d) Opt(TS,CalcFc,Noeigen) Freq=NoRaman Name=TS\\TS 7\\0,1\S
,-4.7610525956,0.8960963686,-0.7491140232\N,-2.7927161492,-0.927980933
2,-1.0190880335\N,-2.2196645041,1.215596984,-1.552555796\N,-1.11842346
17,-0.4578969919,1.9821735299\N,-2.8337421491,1.1005542066,2.127779535
\N,-3.3586170878,-1.1130048695,1.7823675123\C,-3.6125770598,-2.0042129
145,-0.4945326174\C,-3.2178093902,-2.3216362412,0.966451583\C,-4.07696
61247,-3.4512507471,1.5361231322\C,-3.9944147347,-4.6990749803,0.65304
16136\C,-4.3903346994,-4.3854144663,-0.7914597125\C,-3.5342035879,-3.2
573724439,-1.373083012\C,-3.1780178574,0.3681620027,-1.1286447636\C,-2
.4018254105,2.6500589665,-1.7472298167\C,-1.4739073391,3.1096623459,-2
.8759306962\C,-2.1796751352,3.4562018599,-0.4738545854\C,-1.0349296635
,3.272772232,0.3122100028\C,-3.1129638332,4.4202394629,-0.0837400346\C

,-0.8327295419,4.0370434435,1.4620032838\C,-2.9105253496,5.1905202373,
1.0619128452\C,-1.7698838508,5.001025436,1.8398279329\C,-2.4064490093,
-0.1686845333,1.9274872939\H,-3.7539183226,-3.6709712646,2.5605375431\
H,-2.167761595,-2.62993897,0.9703098002\H,-4.6431007007,-1.6299646611,
-0.4988818951\H,-2.9666461304,-5.0875717093,0.6726918994\H,-4.63737520
14,-5.4864514064,1.0651211629\H,-4.2936506315,-5.2831076262,-1.4142807
872\H,-5.4510602041,-4.0956274458,-0.8233791623\H,-2.4903274896,-3.584
2576563,-1.4499587917\H,-3.8704804464,-2.996769306,-2.3836140387\H,-5.
1235100481,-3.1143315602,1.5973405076\H,-1.8121920848,-1.1705271618,-1
.1618549388\H,-1.2535201845,0.88141606,-1.5999072584\H,-3.441825569,2.
790333751,-2.0554498545\H,-1.5829686641,4.1868501397,-3.0361600462\H,-
0.4250562182,2.9036549926,-2.6362144047\H,-1.7218533192,2.5891605128,-
3.8072511696\H,-0.3068356556,2.5108964847,0.0461637802\H,-4.0155792383
,4.5573606355,-0.6751076894\H,0.0612855845,3.8715965246,2.0585494963\H
,-3.6520692829,5.9310768602,1.3516963095\H,-1.6127247049,5.5968836736,
2.7356015398\H,-4.2957074827,-0.7233078263,1.7642105462\H,-3.632739575
7,1.3766195017,1.554331214\H,-2.1201298221,1.8192761579,2.1766825617\H
,-0.4096208171,0.2701375062,1.8647058724\O,1.2864787065,1.0531620216,1
.7890233649\C,2.3507135821,0.6781731275,1.3068404593\C,2.7139835252,0.
5023994739,-0.0963969207\O,0.5393803569,0.5381208338,-0.9878687096\C,1
.7317832681,0.8094626422,-1.1117506665\O,2.1162342729,1.288126074,-2.3
250347923\C,3.4154282267,1.8509685486,-2.5523190165\C,3.5531394792,3.2
399145831,-1.9549345875\O,3.4144956222,0.3912601907,2.0991228247\C,3.1
951034805,0.5142796384,3.5126801889\C,4.4796043506,0.1205037851,4.2095
595594\H,4.3560859775,0.2060846045,5.2945272998\H,4.7494688644,-0.9146
52752,3.9768143966\H,5.3066499851,0.7702921385,3.9055276623\H,2.907969
6593,1.5465715663,3.7416781558\H,2.3597255057,-0.1322120097,3.80113528
01\H,3.7547704214,0.7347104237,-0.2848157003\H,3.4943871535,1.89263010
45,-3.6424017875\H,4.1969739229,1.1709883662,-2.1985893103\H,4.5279123
446,3.6661178659,-2.2182708044\H,2.771986368,3.9029682874,-2.340123591
9\H,3.4755948767,3.215638792,-0.8631908846\H,-0.7372945614,-1.39433458
17,1.814507423\C,6.5831649752,-1.7172822275,-1.2310137807\C,5.38908058
44,-1.6502618964,-0.5194951097\C,4.1505048261,-1.6777800861,-1.1792671
41\C,6.563993502,-1.8080570096,-2.6223291802\C,5.3407423177,-1.8275022
103,-3.2922911731\C,4.1462532639,-1.7621889312,-2.5801081001\H,5.40600
78719,-1.5709592497,0.5649568195\H,7.5303247814,-1.7015836849,-0.69767
05011\H,7.4947405791,-1.8631993762,-3.1809653421\H,5.3153321388,-1.896
6460966,-4.3769718092\H,3.2053541681,-1.7702925265,-3.1222147118\C,2.9
10330751,-1.6278487025,-0.3718846495\C,1.7181212514,-2.1628732794,-0.8
697342198\H,3.0588272204,-1.7489702065,0.6965747369\N,0.6580130901,-2.
4003761315,-0.0437594055\O,-0.4428485157,-2.7360911424,-0.5558639452\O
,0.7803441501,-2.297362415,1.2073682728\H,1.4804169938,-2.290311183,-1
.9148149727\\Version=IA64L-G03RevD.01\State=1-A\HF=-2385.0083267\RMSD=
5.069e-09\RMSF=7.929e-07\Thermal=0.\Dipole=1.665672,0.5090117,0.414521
3\PG=C01 [X(C31H44N6O6S1)]\\@

Fin7

1\1\GINC-A06\Freq\RB3PW91\6-31G(d)\C31H44N6O6S1\TS\20-Dec-2007\0\\#P G
eom=AllCheck Guess=Read SCRF=Check GenChk RB3PW91/6-31G(d) Freq\\Fin T
S 7\\0,1\S,-4.7284503295,0.1240848436,-1.2704593448\N,-2.5101318543,-1
.3980499586,-1.07979092\N,-2.1731147948,0.7224944777,-1.845684911\N,-1
.3492968659,-0.0087514122,1.8417250214\N,-3.4180455535,1.06133479,1.79
66132788\N,-3.3453184638,-1.2288209744,1.6731806576\C,-3.2028945734,-2
.4947108998,-0.4292990896\C,-2.8764707351,-2.484301511,1.0842033973\C,
-3.5196037707,-3.669490117,1.8061148588\C,-3.1167852872,-4.9936271681,
1.1531301829\C,-3.4775790445,-5.0080887215,-0.3335360446\C,-2.82782451
04,-3.8357747569,-1.0710838242\C,-3.0535553825,-0.2059752704,-1.401307
6852\C,-2.5178780246,2.0955786205,-2.2037721403\C,-1.5265124817,2.5985
990707,-3.2549390391\C,-2.5777382059,3.0104647938,-0.9862246276\C,-1.4
689189024,3.1658120103,-0.1452789177\C,-3.7505669491,3.7096820351,-0.6
868817179\C,-1.537120224,3.9961614785,0.9746280258\C,-3.8210416395,4.5
447587709,0.4292090064\C,-2.7152777036,4.6889593112,1.2662886867\C,-2.
6659206054,-0.0708380726,1.735802848\H,-3.2317064352,-3.647593803,2.86
40075648\H,-1.7876194252,-2.5397261446,1.1859668388\H,-4.2765043914,-2
.3148048968,-0.5598857205\H,-2.0330825641,-5.1363514025,1.2653666974\H
,-3.6025072035,-5.8264451877,1.6765180175\H,-3.1608369851,-5.953388868

8,-0.791115044\H,-4.5711511325,-4.958156057,-0.4437709501\H,-1.7372544
301,-3.9456813407,-1.0552507603\H,-3.1397007729,-3.8157052468,-2.12214
8591\H,-4.6157239934,-3.565311447,1.7720885008\H,-1.4869728544,-1.5185
456252,-1.0550431574\H,-1.1791059118,0.5108442867,-1.7971734689\H,-3.5
198239493,2.0553087453,-2.6405047193\H,-1.7625448268,3.6322010348,-3.5
268061523\H,-0.4973227155,2.5675329977,-2.8815136911\H,-1.5832671797,1
.9781402764,-4.1556904825\H,-0.5489586537,2.6229611154,-0.3455609228\H
,-4.6227725555,3.5799676428,-1.3229331382\H,-0.6646877994,4.0980563944
,1.6152438302\H,-4.7446459937,5.0749514045,0.6489890732\H,-2.769612007
9,5.3361340025,2.1383851594\H,-4.3481708843,-1.0942273871,1.6073006377
\H,-4.1872096963,1.0735272796,1.1246286539\H,-2.9037463139,1.935916057
4,1.7956841332\H,-0.8881965681,0.8851640805,1.7042341003\O,1.066489471
2,1.6609782201,1.4229196472\C,2.0710134576,0.9758004999,1.3706938654\C
,2.8473561256,0.6083764975,0.1101831866\O,0.9874351497,0.8274363804,-1
.3764313844\C,2.1329181037,1.1320019491,-1.1220374113\O,2.780584381,1.
9177982272,-2.0053194016\C,4.1059643739,2.435307077,-1.8060425103\C,4.
0919801722,3.6932510591,-0.9581084022\O,2.7538886928,0.58280936,2.4507
124803\C,2.1113328193,0.8110099948,3.7184215398\C,3.0136716911,0.25148
00922,4.7948364747\H,2.555557811,0.4056356553,5.7776658968\H,3.1671320
479,-0.822296422,4.6511342284\H,3.9896028683,0.7476558508,4.7893384395
\H,1.9419548048,1.8864167524,3.8394113884\H,1.1382074006,0.3114042449,
3.7028757824\H,3.8228717595,1.0828919828,0.2481450536\H,4.4529039421,2
.6572028702,-2.8188523672\H,4.7660649465,1.6615263785,-1.4023730335\H,
5.1017335504,4.1142222202,-0.8968671259\H,3.4301386846,4.443906778,-1.
4008026276\H,3.7441224646,3.4925940358,0.060964008\H,-0.6813329275,-0.
8019302247,1.7046994406\C,6.8298330454,-1.6296748638,-0.7824975923\C,5
.6357082085,-1.4257256627,-0.0934921196\C,4.4384025536,-1.1842691334,-
0.7772199235\C,6.8473520593,-1.593726764,-2.1754498759\C,5.6631741387,
-1.351215213,-2.8700033153\C,4.4703387772,-1.1474644775,-2.1777200126\
H,5.627388789,-1.4639910005,0.9939925149\H,7.7452235605,-1.8247370891,
-0.2290920868\H,7.7757612933,-1.7586963287,-2.7162367766\H,5.664672999
7,-1.3260418933,-3.9569766148\H,3.5557810319,-0.9631894784,-2.73645293
69\C,3.1598539536,-0.9578135005,0.0085363025\C,2.0349494962,-1.7696103
48,-0.5207159098\H,3.3440367225,-1.2386924574,1.0515396825\N,0.9322835
456,-1.9092888513,0.1807277206\O,-0.0902001964,-2.514064927,-0.3021439
025\O,0.8657033592,-1.4286928894,1.3830177601\H,2.0204060169,-2.203153
8522,-1.5093941923\\Version=IA64L-G03RevD.01\State=1-A\HF=-2385.024037
8\RMSD=4.247e-09\RMSF=2.495e-05\ZeroPoint=0.745951\Thermal=0.7906297\D
ipole=1.8208898,1.3072189,1.1446356\DipoleDeriv=-0.9297505,0.0540787,-

Init8

1\1\GINC-A06\FOpt\RB3PW91\6-31G(d)\C31H44N6O6S1\TS\20-Dec-2007\0\\#P B
3PW91/6-31G(d) Opt Freq=NoRaman Name=TS\\Init TS 8\\0,1\S,-0.047294816
3,4.9781307905,0.314435248\N,1.1111573629,2.5562404256,0.6352071684\N,
-0.9111240941,2.8024095585,1.6382886314\N,-0.0975174302,0.7339611166,-
2.1866124615\N,-1.3046844327,2.6949005025,-1.9897113429\N,0.9948231701
,2.8050091063,-2.2041564794\C,2.2929583543,2.9362671081,-0.1099289565\
C,2.2500073441,2.4043267882,-1.5611376213\C,3.4640047694,2.8928321811,
-2.351139509\C,4.7615355711,2.4592655357,-1.6625939411\C,4.8120141759,
2.9296528163,-0.2064781065\C,3.5798215193,2.4718420663,0.5801176848\C,
0.057207517,3.3727437651,0.8958120053\C,-2.1754397614,3.4246625247,2.0
041705225\C,-2.5932029422,2.907424215,3.3846473028\C,-3.2745912623,3.1
905294069,0.9768429124\C,-3.4583569294,1.9347684825,0.3858844908\C,-4.
1501026454,4.2273782324,0.6422233143\C,-4.4966432524,1.7257366317,-0.5
221658961\C,-5.1933949968,4.0181092379,-0.2595011357\C,-5.3700236985,2
.7655907118,-0.8448238599\C,-0.1271502036,2.0540678011,-2.0956597924\H
,3.413375723,2.5011691753,-3.3736664267\H,2.2775870063,1.3111128402,-1
.5263988203\H,2.283952746,4.0320827294,-0.1490832154\H,4.8336404131,1.
362799767,-1.6940226833\H,5.6246672609,2.8457293789,-2.2181758965\H,5.
723221186,2.5597705051,0.2795132206\H,4.8682636261,4.027566923,-0.1804
136614\H,3.5765970292,1.3754566838,0.6590739892\H,3.6006681082,2.87104
73108,1.600838155\H,3.4307979303,3.9901344648,-2.4263674318\H,0.983654
4756,1.5675402306,0.8704841204\H,-0.8242812063,1.7933144771,1.78770002
76\H,-1.9845950881,4.5000818727,2.0639601441\H,-3.5311183719,3.3782747
823,3.6950364586\H,-2.7563968513,1.8233453582,3.3650260342\H,-1.822348
8135,3.1328332939,4.1294894479\H,-2.7736355106,1.1166505111,0.59713775
03\H,-4.0060769083,5.2116432586,1.0834534962\H,-4.6071867298,0.7445312

584,-0.9761303197\H,-5.860855052,4.8385078668,-0.5122177902\H,-6.17933
36322,2.6018196121,-1.5522642662\H,0.807231664,3.801302151,-2.11596777
46\H,-1.3046076379,3.5741518112,-1.4720460747\H,-2.1381235769,2.131269
8042,-1.8692609184\H,-0.8504365794,0.1688098498,-1.7414303637\O,-2.099
9419893,-0.7573033199,-1.0572414513\C,-1.9170571413,-1.8220668156,-0.4
299373372\C,-1.0670981516,-2.0964125073,0.6648192173\O,-0.2711130695,0
.100174506,1.2130059929\C,-0.3218636044,-1.1380169584,1.3686884104\O,0
.4412059803,-1.7234430689,2.36616426\C,1.1631040829,-0.8349679025,3.21
4318767\C,1.8253571396,-1.6658257658,4.2950726606\O,-2.6087594442,-2.9
40964249,-0.812660687\C,-3.4737343108,-2.7975084846,-1.9357958884\C,-4
.0384743727,-4.1682288302,-2.250581041\H,-4.7183195702,-4.1098873178,-
3.1080058529\H,-3.2363605486,-4.8731897313,-2.4928506584\H,-4.59474315
33,-4.5658847949,-1.395457533\H,-4.2734680777,-2.0842457928,-1.7000921
158\H,-2.9171286941,-2.386290498,-2.7862838642\H,-1.0483711825,-3.1161
575893,1.0274920304\H,0.4775480681,-0.0981405318,3.6498438927\H,1.9091
290772,-0.2825656892,2.6301106411\H,2.3809969914,-1.0180230623,4.98213
99022\H,1.0776516144,-2.2192777024,4.8726049297\H,2.5286124171,-2.3860
858998,3.8631610316\H,0.773761294,0.2580974849,-2.3984429926\C,1.90220
90244,-7.4414259125,-0.3395184968\C,2.0888435616,-6.1145976726,-0.7010
366383\C,1.7823584865,-5.0808442472,0.2026331435\C,1.4053954811,-7.761
2461396,0.9270654792\C,1.1002506437,-6.7471388973,1.8334496128\C,1.291
6963243,-5.4168146303,1.4767780048\H,2.48672991,-5.8783520612,-1.68419
46933\H,2.145841605,-8.2324396201,-1.0435786335\H,1.25974729,-8.801831
9744,1.204974272\H,0.7126127082,-6.9926560287,2.8182644709\H,1.0462267
245,-4.6183494457,2.1725105537\C,1.9480727352,-3.6712772337,-0.1086135
248\C,2.1938200414,-3.1404656154,-1.3197162497\H,1.826268546,-2.973391
3283,0.7171024301\N,2.2707510759,-1.7199508514,-1.4626572273\O,2.16635
16254,-1.2757366703,-2.6157751417\O,2.4172663238,-1.008719363,-0.47163
19176\H,2.2415600088,-3.6526127085,-2.2705151375\\Version=IA64L-G03Rev
D.01\State=1-A\HF=-2385.0283309\RMSD=6.444e-09\RMSF=3.199e-06\Thermal=
0.\Dipole=1.2677925,-1.9802523,-0.2480483\PG=C01 [X(C31H44N6O6S1)]\\@

TS8

1\1\GINC-A02\FTS\RB3PW91\6-31G(d)\C31H44N6O6S1\TS\29-Nov-2007\0\\#P B3
PW91/6-31G(d) Opt(TS,CalcFc,Noeigen) Freq=NoRaman Name=TS\\TS 8\\0,1\S
,4.8878036468,0.7102808715,-0.1600233022\N,2.7941974367,-0.7074228198,
0.7933336414\N,2.6596682209,1.5416887926,1.0900080037\N,0.6358215362,-
0.7889904169,-2.0401952586\N,2.3334817585,0.7197232961,-2.5233744167\N
,2.8788431414,-1.4464746114,-1.9560361647\C,3.3641345273,-1.9844731934
,0.4054872267\C,2.7721054899,-2.4877128057,-0.9304735146\C,3.457421561
5,-3.7819955922,-1.3663176988\C,3.2811271424,-4.8541166894,-0.28693172
18\C,3.798556316,-4.3750021897,1.0724385811\C,3.154643079,-3.048170136
1,1.4882952902\C,3.3787082545,0.5028831393,0.61250411\C,2.9980662485,2
.9487014802,0.9284610328\C,2.4638152131,3.7245202376,2.1360078489\C,2.
4796226879,3.5367653657,-0.377760449\C,1.1693231439,3.29682466,-0.8099
541277\C,3.3021967463,4.3593060903,-1.1523973015\C,0.6967533761,3.8674
545947,-1.9918244901\C,2.8302568852,4.9357022777,-2.3318379214\C,1.525
4512955,4.6914841081,-2.7558839326\C,1.9281908425,-0.5064117825,-2.137
1668873\H,3.0280579332,-4.1148080665,-2.3181957405\H,1.7108789487,-2.7
093969565,-0.7783791026\H,4.4371865482,-1.8074562882,0.2687355056\H,2.
2132427612,-5.0979440961,-0.2059206639\H,3.7967114383,-5.7744967052,-0
.5872037923\H,3.6058046663,-5.1353031887,1.8391113977\H,4.8901967026,-
4.2482625012,1.0249087146\H,2.0767458183,-3.1929201119,1.6454893226\H,
3.5833431675,-2.6892116279,2.4316737937\H,4.5279103787,-3.5943861741,-
1.5418357722\H,1.8243462716,-0.7057245689,1.1102136251\H,1.7084795256,
1.3269708427,1.3907636985\H,4.0903773779,3.0034823873,0.9187650316\H,2
.7280198571,4.7829472971,2.0502332613\H,1.3706546618,3.660092751,2.195
0879606\H,2.8891662413,3.3296374574,3.0647971279\H,0.5187905916,2.6324
725422,-0.2459905024\H,4.3274067153,4.5388557853,-0.8357038878\H,-0.32
06211178,3.6580351489,-2.3125183378\H,3.4876422557,5.567363735,-2.9241
808025\H,1.1571174615,5.1359551228,-3.6771141675\H,3.8223041794,-1.086
3690624,-2.0742678133\H,3.2156160853,1.0486970726,-2.1308027732\H,1.62
93554655,1.4379276763,-2.6380154908\H,-0.0425369769,-0.0597647891,-1.7
935433262\O,-1.5162606475,0.9740921237,-1.2957597333\C,-2.3693390013,1
.0674440337,-0.411023606\C,-2.3437662252,0.5061599055,0.9206853105\O,0
.045186814,0.3991324598,1.1609247295\C,-1.097877247,0.3141474634,1.623
0651635\O,-1.3228995066,0.0004221761,2.920464259\C,-0.1792581861,-0.36

12184899,3.7038823482\C,-0.6717411852,-0.7127988893,5.0913802893\O,-3.
5452999901,1.7005450201,-0.6426781029\C,-3.7446423937,2.2175259107,-1.
9630348069\C,-5.1422154884,2.7969692135,-2.0158710546\H,-5.3430010952,
3.2022969784,-3.0136563286\H,-5.8877494019,2.0270934872,-1.7951685501\
H,-5.257142744,3.6047023254,-1.2860490022\H,-2.9843043089,2.9795188553
,-2.1709353874\H,-3.6070614213,1.4124321272,-2.69323235\H,-3.185910699
9,0.7688863749,1.5497413622\H,0.5250029694,0.478653517,3.7303092363\H,
0.3185658827,-1.2113950245,3.2260084801\H,0.1746521803,-0.9914636095,5
.7282264912\H,-1.1852240153,0.1370460232,5.552489682\H,-1.3657999638,-
1.5581710938,5.0533739214\H,0.3347579944,-1.7469564731,-1.8319813853\C
,-6.4485559037,-1.1426210557,-0.9326377566\C,-5.0713426061,-1.30317292
59,-0.8164239973\C,-4.4595442769,-1.3592084177,0.4448208108\C,-7.24348
99077,-1.0288773862,0.2080335148\C,-6.6486095986,-1.0762579719,1.46810
09101\C,-5.2717895522,-1.2377811444,1.5827039207\H,-4.469281574,-1.389
6523311,-1.7163491436\H,-6.9044565472,-1.11086009,-1.9191273631\H,-8.3
194469233,-0.9064064453,0.1147485505\H,-7.2585537244,-0.9915006564,2.3
638237777\H,-4.8110411508,-1.2769087661,2.5674357945\C,-3.0113170568,-
1.5812913796,0.626801408\C,-2.2514476434,-2.2201783029,-0.3504654991\H
,-2.6973683212,-1.7677961275,1.6497205773\N,-1.0205915423,-2.749763723
,-0.0535682437\O,-0.3607359624,-3.2602927739,-1.0121630202\O,-0.567216
2044,-2.7377755208,1.1090120505\H,-2.5182863326,-2.3113177552,-1.39235
90714\\Version=IA64L-G03RevD.01\State=1-A\HF=-2385.0190008\RMSD=8.012e
-09\RMSF=2.464e-06\Thermal=0.\Dipole=-1.0091578,0.9480822,-0.1225105\P
G=C01 [X(C31H44N6O6S1)]\\@

Fin8

1\1\GINC-A06\FOpt\RB3PW91\6-31G(d)\C31H44N6O6S1\TS\20-Dec-2007\0\\#P B
3PW91/6-31G(d) Opt Freq=NoRaman Name=TS\\Fin TS8\\0,1\S,-0.0944834667,
4.758309844,0.6559886373\N,1.2312774342,2.3989775952,0.6099469696\N,-0
.8966639957,2.3078428002,1.41098624\N,0.0762624246,1.3884504688,-2.509
1803357\N,-0.9851026788,3.4614440136,-2.3887331424\N,1.2889117394,3.35
01663307,-2.0903568309\C,2.4363422578,2.9929739773,0.0624089887\C,2.48
69861065,2.7850215749,-1.4689804006\C,3.7486675403,3.4098978456,-2.065
2050837\C,4.9989138926,2.8170802657,-1.4102741725\C,4.9624518749,2.982
3909184,0.1109227676\C,3.6859862123,2.3881891364,0.7120229269\C,0.0945
363227,3.0706714441,0.895913886\C,-2.2913877344,2.7166386664,1.5198002
912\C,-2.9367737809,2.028542019,2.724441059\C,-3.0502471674,2.43461734
59,0.2281821908\C,-2.9546167206,1.1856084752,-0.400432185\C,-3.8449353
948,3.4226743494,-0.3595571932\C,-3.6271989594,0.9388997685,-1.5973400
726\C,-4.5237557686,3.1780516389,-1.5557222867\C,-4.4123725388,1.93708
52115,-2.1824924201\C,0.1346323341,2.6964465093,-2.2984614297\H,3.7582
612209,3.2420494982,-3.1485160934\H,2.4976529787,1.7055491405,-1.65907
2294\H,2.3893296737,4.0670664495,0.2768960579\H,5.0600335902,1.7489900
134,-1.6601400351\H,5.8968264821,3.2914162385,-1.8251656149\H,5.841993
1999,2.5093339688,0.5648141218\H,5.0189616295,4.0522349305,0.361323877
4\H,3.6630620865,1.2999679192,0.5675621277\H,3.6461668896,2.5729978424
,1.7924591885\H,3.729653501,4.5000057766,-1.9089706448\H,1.216423792,1
.3663692591,0.5493075414\H,-0.7149411669,1.3134801105,1.5535136244\H,-
2.2819843755,3.7980048475,1.6821757549\H,-3.9824422495,2.3390850447,2.
8167313613\H,-2.9112707361,0.9390579823,2.6201905168\H,-2.4083042643,2
.3025591515,3.6436696216\H,-2.3399104694,0.4018267789,0.0354380761\H,-
3.9194381821,4.3993429301,0.1140010109\H,-3.5375265151,-0.0394865442,-
2.0639596253\H,-5.1316968298,3.9616686248,-2.0014304175\H,-4.937004053
2,1.7459473353,-3.1154123739\H,1.1569524183,4.339761613,-1.9063741342\
H,-1.029759365,4.2064132494,-1.6912540264\H,-1.8634308305,2.9653347587
,-2.4855440946\H,-0.8379507213,0.9592103679,-2.455351639\O,-1.57084943
4,-2.0343047329,-0.9279678892\C,-1.476167203,-2.6661936874,0.102315049
6\C,-0.2369007392,-2.7796423546,0.971493194\O,-0.8887987869,-0.5980815
149,1.8295666022\C,-0.1181140035,-1.5438714117,1.848799704\O,0.8342180
729,-1.7116699252,2.7650186938\C,1.0948015992,-0.5934153349,3.63344823
77\C,2.1770426998,-1.0131367711,4.602742124\O,-2.4847829883,-3.3728756
587,0.6381705008\C,-3.7366864504,-3.3296503187,-0.0712709299\C,-4.7072
273287,-4.2280212631,0.6631243291\H,-5.6768621905,-4.222423127,0.15383
1786\H,-4.3394339103,-5.2584279172,0.6932330194\H,-4.8570797574,-3.884
4485426,1.691436819\H,-4.0842865821,-2.2916357777,-0.1063860522\H,-3.5
68918975,-3.6600408332,-1.1017242309\H,-0.4002778502,-3.6083066438,1.6
700664248\H,0.1664439384,-0.3192903674,4.1450475686\H,1.410551722,0.25

15656781,3.0143671065\H,2.4074395663,-0.1845374481,5.2808105145\H,1.85
6464312,-1.8702328423,5.2036818673\H,3.0922349426,-1.2855525122,4.0683
217874\H,0.887967037,0.7345916449,-2.3237163377\C,0.2837617681,-6.4019
984187,-1.6288338816\C,0.3146862742,-5.048668527,-1.298080975\C,1.0623
549479,-4.5959311994,-0.2034898025\C,0.9991596683,-7.327479736,-0.8705
790495\C,1.74638562,-6.8888170711,0.2206752201\C,1.7753740222,-5.53458
45215,0.5489816298\H,-0.2431715935,-4.3347534389,-1.8994410857\H,-0.29
90893006,-6.7334786167,-2.4848986435\H,0.9774260478,-8.3826731141,-1.1
31003118\H,2.3131783478,-7.6004326254,0.8161103113\H,2.364906238,-5.19
85730007,1.3997888023\C,1.0849608448,-3.1283835526,0.1911621809\C,1.40
42384351,-2.2700085459,-0.9870891135\H,1.8691754644,-2.9891018382,0.94
76742912\N,1.6911870385,-0.9989013771,-0.858511905\O,2.0449035638,-0.3
08533002,-1.9030649429\O,1.640917289,-0.4230270381,0.29860217\H,1.4791
899965,-2.6629902009,-1.9899022683\\Version=IA64L-G03RevD.01\State=1-A
\HF=-2385.0417865\RMSD=4.712e-09\RMSF=2.925e-06\Thermal=0.\Dipole=-1.1
95339,-0.8127999,0.1314838\PG=C01 [X(C31H44N6O6S1)]\\@

Init9

1\1\FAU-CCC-CCDH153\SP\RB3PW91\6-31G(d)\C31H44N6O6S1\SHUBINA\28-Jul-20
08\0\\#P B3PW91/6-31G(d) SCF=Tight Name=SHUBINA\\Init TS 9 R\\0,1\S,0,
-4.870142,-0.373631,0.660285\N,0,-2.530248,0.869655,1.223238\N,0,-2.54
0862,-1.384237,1.534337\N,0,-2.954295,-0.444412,-2.596788\N,0,-1.09151
7,0.786433,-1.97522\N,0,-3.248193,1.519079,-1.419021\C,0,-3.08969,2.17
3889,0.940103\C,0,-2.839887,2.60169,-0.525273\C,0,-3.590505,3.900761,-
0.822994\C,0,-3.12028,5.004767,0.131097\C,0,-3.260185,4.590169,1.59950
6\C,0,-2.566238,3.254509,1.888354\C,0,-3.231793,-0.285351,1.162146\C,0
,-3.013738,-2.748946,1.365018\C,0,-2.330712,-3.647949,2.398343\C,0,-2.
799125,-3.269178,-0.050834\C,0,-3.804742,-3.993425,-0.696607\C,0,-1.58
7984,-3.057405,-0.718889\C,0,-3.608305,-4.501471,-1.980642\C,0,-1.3868
25,-3.563664,-2.003794\C,0,-2.397917,-4.288516,-2.639332\C,0,-2.408547
,0.62198,-1.957312\H,0,-3.42425,4.19102,-1.867227\H,0,-1.771164,2.7813
41,-0.658529\H,0,-4.173655,2.075175,1.077315\H,0,-2.066231,5.233502,-0
.081867\H,0,-3.68434,5.926242,-0.059201\H,0,-2.851154,5.372117,2.25111
4\H,0,-4.327506,4.502446,1.849976\H,0,-1.480424,3.349651,1.759421\H,0,
-2.747869,2.943141,2.923671\H,0,-4.671459,3.735451,-0.703587\H,0,-1.51
4855,0.801359,1.376184\H,0,-1.559023,-1.241915,1.783524\H,0,-4.090048,
-2.735927,1.560226\H,0,-2.707146,-4.671952,2.312908\H,0,-1.246146,-3.6
78984,2.242409\H,0,-2.527945,-3.284183,3.412381\H,0,-4.757299,-4.14643
9,-0.19383\H,0,-0.800312,-2.476046,-0.246564\H,0,-4.405015,-5.057045,-
2.469499\H,0,-0.436132,-3.387654,-2.50063\H,0,-2.242002,-4.683479,-3.6
40276\H,0,-4.148274,1.112308,-1.146497\H,0,-0.608402,1.33651,-1.244187
\H,0,-0.511425,0.109301,-2.462633\H,0,-2.392549,-1.289634,-2.608834\H,
0,-3.925438,-0.634819,-2.380062\O,0,0.181959,2.15385,0.078635\C,0,1.38
1842,2.037836,0.419612\C,0,1.981381,1.036616,1.202253\O,0,0.05431,-0.2
57616,1.820129\C,0,1.277853,-0.030883,1.793912\O,0,2.139384,-0.909419,
2.422649\C,0,1.537416,-1.976349,3.150211\C,0,2.625127,-2.66217,3.9523\
O,0,2.289584,2.97585,0.002221\C,0,1.785142,4.031649,-0.809566\C,0,2.94
8645,4.945403,-1.138479\H,0,2.610992,5.778498,-1.765053\H,0,3.389955,5
.356406,-0.224729\H,0,3.729885,4.401201,-1.679176\H,0,1.332843,3.62056
9,-1.721\H,0,0.995325,4.570525,-0.271912\H,0,3.044395,1.113768,1.38991
3\H,0,1.062829,-2.678569,2.452495\H,0,0.750502,-1.580919,3.802103\H,0,
2.201833,-3.491113,4.530366\H,0,3.40314,-3.067247,3.296223\H,0,3.09385
5,-1.959935,4.649383\C,0,7.616648,-0.285504,-1.049239\C,0,6.303404,-0.
469018,-1.459007\C,0,5.291383,-0.720994,-0.514988\C,0,7.943911,-0.3474
68,0.307967\C,0,6.95123,-0.598149,1.254005\C,0,5.63527,-0.789201,0.846
767\H,0,6.063134,-0.432542,-2.518088\H,0,8.39138,-0.096608,-1.787428\H
,0,8.973561,-0.202535,0.624418\H,0,7.202247,-0.645102,2.310099\H,0,4.8
52384,-0.973305,1.5786\C,0,3.897535,-0.922518,-0.874373\C,0,3.335243,-
0.684153,-2.072831\H,0,3.23641,-1.276225,-0.087245\N,0,1.932014,-0.894
362,-2.246286\O,0,1.421294,-0.421761,-3.271716\O,0,1.293134,-1.503058,
-1.39043\H,0,3.79869,-0.249851,-2.947549\\Version=AM64L-G03RevD.02\Sta
te=1-A\HF=-2385.0275836\RMSD=6.107e-09\Thermal=0.\Dipole=1.7512389,-0.
318148,-0.9042765\PG=C01 [X(C31H44N6O6S1)]\\@

TS9

1\1\FAU-CCC-CCDH153\SP\RB3PW91\6-31G(d)\C31H44N6O6S1\SHUBINA\28-Jul-20

08\0\\#P B3PW91/6-31G(d) SCF=Tight Name=SHUBINA\\TS9 R\\0,1\S,0,-4.704
873,1.09777,0.512593\N,0,-2.07725,1.50937,0.944305\N,0,-2.786495,-0.63
9279,1.224911\N,0,-2.981853,-0.108941,-2.264498\N,0,-0.702529,0.243804
,-2.019157\N,0,-2.214953,2.026045,-1.864858\C,0,-2.109074,2.867495,0.4
38332\C,0,-1.478399,2.92581,-0.973251\C,0,-1.488292,4.350851,-1.52772\
C,0,-0.78986,5.317848,-0.568916\C,0,-1.413822,5.263978,0.827655\C,0,-1
.405359,3.840482,1.389656\C,0,-3.110646,0.637694,0.918816\C,0,-3.75357
5,-1.707063,1.479377\C,0,-3.156406,-2.663737,2.514904\C,0,-4.194747,-2
.446104,0.222744\C,0,-5.550528,-2.519586,-0.106624\C,0,-3.264526,-3.09
6752,-0.597122\C,0,-5.975077,-3.229531,-1.229823\C,0,-3.687219,-3.8003
21,-1.72539\C,0,-5.04444,-3.871906,-2.04388\C,0,-1.937889,0.714168,-2.
013503\H,0,-1.010305,4.358587,-2.514195\H,0,-0.444409,2.572017,-0.9036
13\H,0,-3.167381,3.143817,0.357165\H,0,0.275737,5.056237,-0.503586\H,0
,-0.838216,6.336894,-0.971708\H,0,-0.876581,5.935144,1.508873\H,0,-2.4
48765,5.632974,0.778086\H,0,-0.372196,3.50401,1.542391\H,0,-1.907788,3
.804056,2.36349\H,0,-2.530883,4.671782,-1.675786\H,0,-1.140378,1.17325
6,1.183213\H,0,-1.802213,-0.908401,1.228532\H,0,-4.635849,-1.219945,1.
905031\H,0,-3.865112,-3.468587,2.732301\H,0,-2.230252,-3.118373,2.1462
41\H,0,-2.934072,-2.131022,3.446026\H,0,-6.278083,-2.001859,0.513869\H
,0,-2.201265,-3.034942,-0.377766\H,0,-7.034249,-3.272549,-1.471975\H,0
,-2.951167,-4.302012,-2.349585\H,0,-5.372908,-4.424202,-2.920966\H,0,-
3.210139,2.223403,-1.90047\H,0,0.12929,0.813747,-1.832204\H,0,-0.56313
3,-0.764052,-1.893915\H,0,-2.786007,-1.101723,-2.320085\H,0,-3.836615,
0.098728,-1.744896\O,0,1.913765,1.21303,-1.590951\C,0,2.903841,1.07518
,-0.86828\C,0,2.956485,0.711361,0.520973\O,0,0.698526,1.278815,1.07757
1\C,0,1.834076,0.919013,1.390833\O,0,2.16186,0.642698,2.684194\C,0,1.1
05283,0.750676,3.643353\C,0,1.696255,0.466634,5.008242\O,0,4.155421,1.
209895,-1.383854\C,0,4.230177,1.525918,-2.777424\C,0,5.696377,1.639924
,-3.138057\H,0,5.804783,1.874466,-4.202707\H,0,6.179534,2.433373,-2.55
8952\H,0,6.221065,0.701311,-2.933671\H,0,3.728019,0.743121,-3.357815\H
,0,3.691858,2.461773,-2.966592\H,0,3.934712,0.70723,0.983622\H,0,0.316
124,0.03316,3.390514\H,0,0.669842,1.755012,3.59524\H,0,0.918168,0.5401
13,5.77576\H,0,2.122926,-0.540825,5.048286\H,0,2.486509,1.185143,5.248
473\C,0,6.306362,-2.378071,-1.015789\C,0,4.941369,-2.122996,-0.942068\
C,0,4.323986,-1.879811,0.295129\C,0,7.084475,-2.392048,0.142065\C,0,6.
486348,-2.145363,1.377496\C,0,5.121675,-1.890331,1.451401\H,0,4.353602
,-2.110959,-1.855047\H,0,6.766077,-2.568803,-1.982206\H,0,8.150811,-2.
59328,0.081042\H,0,7.083957,-2.15347,2.285336\H,0,4.656723,-1.691371,2
.41425\C,0,2.880969,-1.652416,0.440645\C,0,1.961378,-2.050468,-0.51330
9\H,0,2.512741,-1.580095,1.458661\N,0,0.614874,-2.104114,-0.237593\O,0
,-0.155611,-2.400139,-1.19813\O,0,0.169389,-1.881285,0.909392\H,0,2.18
8912,-2.292685,-1.540469\\Version=AM64L-G03RevD.02\State=1-A\HF=-2385.
0171108\RMSD=4.259e-09\Thermal=0.\Dipole=1.2645905,0.452724,-0.3944132
\PG=C01 [X(C31H44N6O6S1)]\\@

Fin9

1\1\FAU-CCC-CCDH154\SP\RB3PW91\6-31G(d)\C31H44N6O6S1\SHUBINA\28-Jul-20
08\0\\#P B3PW91/6-31G(d) SCF=Tight Name=SHUBINA\\Fin TS9 R\\0,1\S,0,-4
.4365,1.26469,0.34854\N,0,-1.76017,1.5415,0.51121\N,0,-2.5722,-0.46985
,1.21331\N,0,-2.93345,-0.42881,-2.27356\N,0,-0.64957,-0.0949,-2.3257\N
,0,-2.12831,1.73552,-2.313\C,0,-1.78821,2.83627,-0.1358\C,0,-1.31333,2
.72583,-1.60373\C,0,-1.3835,4.08146,-2.30831\C,0,-0.58331,5.13935,-1.5
4445\C,0,-1.04123,5.24352,-0.08796\C,0,-0.97288,3.88853,0.62166\C,0,-2
.84687,0.75078,0.72061\C,0,-3.58363,-1.41382,1.68661\C,0,-2.96782,-2.2
4495,2.81659\C,0,-4.16554,-2.30651,0.59737\C,0,-5.54862,-2.35218,0.402
15\C,0,-3.345,-3.13268,-0.18117\C,0,-6.10899,-3.20681,-0.54721\C,0,-3.
90545,-3.9816,-1.13628\C,0,-5.28824,-4.0252,-1.32038\C,0,-1.87461,0.40
644,-2.26664\H,0,-1.02091,3.97695,-3.33775\H,0,-0.27361,2.37937,-1.608
13\H,0,-2.84099,3.14429,-0.14738\H,0,0.48416,4.87513,-1.57218\H,0,-0.6
7712,6.10926,-2.04793\H,0,-0.4277,5.97673,0.44988\H,0,-2.07399,5.62024
,-0.05898\H,0,0.06905,3.55647,0.70544\H,0,-1.36325,3.96588,1.64295\H,0
,-2.43615,4.39559,-2.37337\H,0,-0.8495,1.21499,0.83388\H,0,-1.59717,-0
.81516,1.16273\H,0,-4.40304,-0.81004,2.08844\H,0,-3.69474,-2.97497,3.1
8578\H,0,-2.08296,-2.78768,2.46713\H,0,-2.6681,-1.59719,3.64808\H,0,-6
.19083,-1.70042,0.99008\H,0,-2.26264,-3.08961,-0.07182\H,0,-7.18736,-3
.22579,-0.68649\H,0,-3.25521,-4.61803,-1.73261\H,0,-5.72178,-4.69121,-
2.06268\H,0,-3.12217,1.9423,-2.26642\H,0,0.15918,0.51347,-2.28076\H,0,

-0.51267,-1.04624,-1.89633\H,0,-2.73414,-1.41701,-2.16462\H,0,-3.74709
,-0.12085,-1.7379\O,0,2.07697,0.89348,-1.37883\C,0,2.91161,0.92577,-0.
49585\C,0,2.92042,0.08513,0.76519\O,0,1.03703,1.45213,1.48529\C,0,1.84
09,0.56912,1.72133\O,0,1.99517,-0.0013,2.91578\C,0,0.99384,0.31439,3.9
0269\C,0,1.33611,-0.455,5.15858\O,0,3.97934,1.73478,-0.52978\C,0,4.096
75,2.58933,-1.68345\C,0,5.38958,3.36228,-1.54783\H,0,5.51638,4.02648,-
2.40931\H,0,5.38787,3.97286,-0.63955\H,0,6.2469,2.68342,-1.5064\H,0,4.
08016,1.96761,-2.58455\H,0,3.22364,3.24972,-1.71991\H,0,3.8691,0.27327
,1.27959\H,0,0.01835,0.02755,3.49986\H,0,0.9922,1.39681,4.06778\H,0,0.
59468,-0.24184,5.93594\H,0,1.33314,-1.53219,4.96616\H,0,2.32303,-0.171
36,5.53826\C,0,5.90753,-2.16352,-1.71943\C,0,4.64745,-1.74457,-1.29742
\C,0,4.22922,-1.94817,0.0238\C,0,6.77353,-2.79299,-0.82661\C,0,6.36878
,-3.00101,0.49039\C,0,5.10761,-2.58086,0.90897\H,0,3.97787,-1.26147,-2
.00561\H,0,6.21172,-2.00102,-2.75066\H,0,7.75529,-3.12276,-1.15674\H,0
,7.03329,-3.49626,1.19409\H,0,4.7968,-2.75123,1.93772\C,0,2.87098,-1.4
7312,0.51083\C,0,1.78531,-1.94637,-0.39393\H,0,2.69874,-1.88566,1.5134
1\N,0,0.52054,-1.88505,-0.04852\O,0,-0.39372,-2.31145,-0.8683\O,0,0.16
111,-1.38915,1.08685\H,0,1.98334,-2.40745,-1.35017\\Version=AM64L-G03R
evD.02\State=1-A\HF=-2385.0420358\RMSD=8.889e-09\Thermal=0.\Dipole=2.1
224925,1.5761175,-0.4381603\PG=C01 [X(C31H44N6O6S1)]\\@

Init10

1\1\FAU-CCC-CCDH155\SP\RB3PW91\6-31G(d)\C31H44N6O6S1\SHUBINA\28-Jul-20
08\0\\#P B3PW91/6-31G(d) SCF=Tight Name=SHUBINA\\Init TS10 S\\0,1\S,0,
-4.68922,1.67535,0.10279\N,0,-2.05948,2.11013,0.58356\N,0,-2.96102,0.3
2418,1.65864\N,0,-2.96347,-0.38985,-2.27095\N,0,-0.74269,0.1132,-1.798
37\N,0,-2.29846,1.81395,-2.22612\C,0,-2.02466,3.27978,-0.26613\C,0,-1.
52856,2.93323,-1.68965\C,0,-1.63098,4.15978,-2.59789\C,0,-0.80093,5.31
105,-2.02053\C,0,-1.20368,5.6361,-0.57936\C,0,-1.15828,4.39687,0.32144
\C,0,-3.16241,1.35798,0.81234\C,0,-3.93009,-0.72612,1.92966\C,0,-3.698
15,-1.27386,3.33998\C,0,-3.89281,-1.83718,0.88771\C,0,-5.08385,-2.3374
,0.35463\C,0,-2.67981,-2.38863,0.45569\C,0,-5.07012,-3.36689,-0.58691\
C,0,-2.65946,-3.41601,-0.48993\C,0,-3.85826,-3.90785,-1.01305\C,0,-1.9
8048,0.5194,-2.05379\H,0,-1.28845,3.89915,-3.60631\H,0,-0.47903,2.6362
2,-1.63278\H,0,-3.06073,3.63058,-0.34552\H,0,0.26152,5.03061,-2.04554\
H,0,-0.9058,6.19987,-2.65475\H,0,-0.5493,6.41701,-0.17288\H,0,-2.22309
,6.04871,-0.57222\H,0,-0.12561,4.03622,0.41954\H,0,-1.51776,4.63852,1.
32841\H,0,-2.68445,4.46607,-2.68539\H,0,-1.16221,1.72852,0.91047\H,0,-
1.99742,0.17489,1.96864\H,0,-4.9167,-0.25543,1.88706\H,0,-4.44069,-2.0
4389,3.57109\H,0,-2.70639,-1.73194,3.4298\H,0,-3.78085,-0.47148,4.0807
3\H,0,-6.03091,-1.90396,0.66883\H,0,-1.73871,-1.99744,0.83696\H,0,-6.0
0736,-3.73764,-0.99497\H,0,-1.70727,-3.81964,-0.8245\H,0,-3.84335,-4.7
0652,-1.75047\H,0,-3.30205,1.98169,-2.21301\H,0,-0.03327,0.74296,-1.38
468\H,0,-0.58426,-0.86856,-1.59599\H,0,-2.78542,-1.33187,-1.93812\H,0,
-3.8969,-0.07274,-2.02269\O,0,1.14565,1.75442,-0.58069\C,0,2.14505,1.4
001,0.08182\C,0,2.21256,0.65484,1.26949\O,0,-0.12422,0.34657,1.73037\C
,0,1.08729,0.19846,1.98953\O,0,1.46254,-0.49003,3.11176\C,0,0.42109,-0
.97533,3.95047\C,0,1.07145,-1.59163,5.17293\O,0,3.39832,1.76036,-0.367
66\C,0,3.44155,2.53267,-1.56516\C,0,4.89813,2.8049,-1.88277\H,0,4.9789
4,3.41166,-2.79152\H,0,5.38028,3.34696,-1.06264\H,0,5.44548,1.8705,-2.
04573\H,0,2.94957,1.98801,-2.38018\H,0,2.88678,3.46759,-1.42096\H,0,3.
18568,0.48417,1.71222\H,0,-0.17676,-1.71885,3.40682\H,0,-0.24983,-0.15
329,4.22794\H,0,0.30642,-1.98813,5.84965\H,0,1.73826,-2.41139,4.88671\
H,0,1.66067,-0.84553,5.71565\C,0,7.02348,-3.44215,-0.51295\C,0,5.64695
,-3.53642,-0.65925\C,0,4.83226,-2.40021,-0.49552\C,0,7.61363,-2.21382,
-0.20228\C,0,6.81864,-1.08134,-0.03546\C,0,5.43721,-1.17144,-0.17554\H
,0,5.19944,-4.50081,-0.88349\H,0,7.64151,-4.32758,-0.63498\H,0,8.69214
,-2.14474,-0.08598\H,0,7.27317,-0.12521,0.2089\H,0,4.81696,-0.28618,-0
.05666\C,0,3.3856,-2.43149,-0.6434\C,0,2.66675,-3.44729,-1.15277\H,0,2
.83935,-1.54734,-0.31167\N,0,1.24014,-3.35803,-1.25656\O,0,0.66235,-4.
33064,-1.73955\O,0,0.66637,-2.3356,-0.86711\H,0,3.03725,-4.38705,-1.53
84\\Version=AM64L-G03RevD.02\State=1-A\HF=-2385.0300439\RMSD=1.742e-09
\Thermal=0.\Dipole=1.6317228,-0.6308108,-0.8722403\PG=C01 [X(C31H44N6O
6S1)]\\@

TS10

1\1\FAU-CCC-CCDH155\SP\RB3PW91\6-31G(d)\C31H44N6O6S1\SHUBINA\28-Jul-20
08\0\\#P B3PW91/6-31G(d) SCF=Tight Name=SHUBINA\\TS10 S\\0,1\S,0,-4.76
9333,1.034621,0.670417\N,0,-2.126551,1.322384,1.106951\N,0,-2.920405,-
0.811715,1.293602\N,0,-3.076326,0.017031,-2.131892\N,0,-0.794072,0.341
305,-1.865277\N,0,-2.283688,2.128612,-1.646771\C,0,-2.143336,2.727109,
0.744184\C,0,-1.550532,2.93855,-0.669891\C,0,-1.589312,4.415266,-1.064
465\C,0,-0.857877,5.276341,-0.031769\C,0,-1.423668,5.063907,1.374101\C
,0,-1.39738,3.585462,1.770803\C,0,-3.193285,0.48617,1.049485\C,0,-3.91
4884,-1.884662,1.311408\C,0,-3.51626,-2.917684,2.368039\C,0,-4.110624,
-2.518016,-0.059733\C,0,-5.380844,-2.5499,-0.641588\C,0,-3.033976,-3.0
85756,-0.755147\C,0,-5.579681,-3.135914,-1.892568\C,0,-3.231076,-3.666
3,-2.008434\C,0,-4.504954,-3.6947,-2.580919\C,0,-2.027433,0.817349,-1.
848723\H,0,-1.146422,4.534556,-2.060009\H,0,-0.509726,2.600154,-0.6644
94\H,0,-3.198379,3.024304,0.726116\H,0,0.210379,5.017081,-0.038905\H,0
,-0.9256,6.333444,-0.316195\H,0,-0.858293,5.655728,2.104402\H,0,-2.459
647,5.431171,1.409454\H,0,-0.35863,3.236434,1.840741\H,0,-1.857845,3.4
38431,2.754896\H,0,-2.637681,4.743341,-1.138573\H,0,-1.194664,0.933148
,1.254811\H,0,-1.945151,-1.120503,1.343875\H,0,-4.858503,-1.415502,1.6
02481\H,0,-4.260879,-3.719247,2.403414\H,0,-2.543357,-3.364882,2.14029
8\H,0,-3.460236,-2.448433,3.356246\H,0,-6.218634,-2.098115,-0.115669\H
,0,-2.033532,-3.050863,-0.33013\H,0,-6.574589,-3.147564,-2.331064\H,0,
-2.38535,-4.103104,-2.534547\H,0,-4.656374,-4.149458,-3.556806\H,0,-3.
271085,2.355061,-1.708304\H,0,0.028278,0.942521,-1.74628\H,0,-0.620161
,-0.635983,-1.609328\H,0,-2.884696,-0.966071,-2.288334\H,0,-3.92914,0.
191908,-1.596852\O,0,1.756153,1.556951,-1.816824\C,0,2.795463,1.220002
,-1.250611\C,0,2.973568,0.688778,0.086138\O,0,0.740178,1.022282,0.8802
99\C,0,1.940971,0.851119,1.081063\O,0,2.434311,0.67756,2.330872\C,0,1.
471842,0.660906,3.395819\C,0,2.223438,0.421555,4.687794\O,0,3.987971,1
.294465,-1.894475\C,0,3.943847,1.783593,-3.24181\C,0,5.365806,1.818643
,-3.759816\H,0,5.378816,2.188075,-4.790952\H,0,5.987689,2.480116,-3.14
834\H,0,5.813454,0.819779,-3.746019\H,0,3.305074,1.127667,-3.844268\H,
0,3.484302,2.778046,-3.248348\H,0,3.989679,0.722178,0.458336\H,0,0.735
224,-0.126588,3.200308\H,0,0.939886,1.618549,3.416353\H,0,1.523349,0.4
16986,5.5301\H,0,2.741634,-0.542691,4.670034\H,0,2.965834,1.207331,4.8
60007\C,0,6.181104,-2.279561,1.616594\C,0,4.835135,-1.977125,1.443333\
C,0,4.298359,-1.813644,0.156478\C,0,7.018706,-2.427956,0.51018\C,0,6.4
99572,-2.262906,-0.772474\C,0,5.15394,-1.952859,-0.946075\H,0,4.199955
,-1.846448,2.314215\H,0,6.57991,-2.400387,2.620649\H,0,8.069647,-2.668
192,0.648772\H,0,7.14361,-2.3753,-1.640935\H,0,4.753251,-1.819513,-1.9
47559\C,0,2.87144,-1.524231,-0.080632\C,0,1.879123,-1.986574,0.791253\
H,0,2.569099,-1.489184,-1.122805\N,0,0.574333,-2.067677,0.393156\O,0,-
0.310083,-2.252422,1.275542\O,0,0.274262,-2.006766,-0.830891\H,0,2.020
289,-2.169809,1.845848\\Version=AM64L-G03RevD.02\State=1-A\HF=-2385.01
78577\RMSD=5.485e-09\Thermal=0.\Dipole=1.2474084,0.8086933,-0.4041417\
PG=C01 [X(C31H44N6O6S1)]\\@

Fin10

1\1\FAU-CCC-CCDH156\SP\RB3PW91\6-31G(d)\C31H44N6O6S1\SHUBINA\28-Jul-20
08\0\\#P B3PW91/6-31G(d) SCF=Tight Name=SHUBINA\\Fin10 S\\0,1\S,0,4.56
495,-2.04026,0.49841\N,0,1.98822,-1.53078,1.14926\N,0,3.39646,0.22219,
1.38971\N,0,3.09654,-0.43772,-2.04413\N,0,0.80218,-0.19385,-1.89753\N,
0,1.79713,-2.29991,-1.61635\C,0,1.5348,-2.85358,0.78372\C,0,0.87797,-2
.86505,-0.62027\C,0,0.4472,-4.27943,-1.00857\C,0,-0.51132,-4.85812,0.0
3607\C,0,0.10387,-4.82867,1.43736\C,0,0.55799,-3.41762,1.82072\C,0,3.2
6182,-1.07146,1.04889\C,0,4.6297,0.99117,1.34081\C,0,4.65928,1.96029,2
.52654\C,0,4.79791,1.72645,0.01729\C,0,6.01508,1.66719,-0.66666\C,0,3.
75325,2.48769,-0.52614\C,0,6.19538,2.35288,-1.86858\C,0,3.9321,3.16996
,-1.73021\C,0,5.15365,3.10674,-2.40465\C,0,1.88419,-0.96568,-1.82201\H
,0,-0.02019,-4.2552,-2.00004\H,0,-0.01271,-2.2282,-0.59101\H,0,2.42919
,-3.48787,0.74576\H,0,-1.43925,-4.27052,0.03689\H,0,-0.78381,-5.88426,
-0.2399\H,0,-0.61838,-5.19983,2.17469\H,0,0.96688,-5.50938,1.47434\H,0
,-0.31817,-2.75705,1.89234\H,0,1.04552,-3.4172,2.80244\H,0,1.33875,-4.
91936,-1.09152\H,0,1.29683,-0.83495,1.4664\H,0,2.5131,0.73216,1.55756\
H,0,5.44885,0.27269,1.4387\H,0,5.58497,2.54448,2.51402\H,0,3.81792,2.6
6142,2.48392\H,0,4.60195,1.4087,3.47074\H,0,6.8264,1.06745,-0.25963\H,
0,2.78095,2.5229,-0.03689\H,0,7.14806,2.28949,-2.38887\H,0,3.11256,3.7
5684,-2.13863\H,0,5.2897,3.64045,-3.34207\H,0,2.70418,-2.75985,-1.6281

3\H,0,-0.11957,-0.6205,-1.93111\H,0,0.80397,0.72654,-1.4077\H,0,3.1530
8,0.55899,-2.21743\H,0,3.88464,-0.84388,-1.53181\O,0,-2.06871,-0.66681
,-2.1653\C,0,-3.14892,-0.48207,-1.64294\C,0,-3.40356,0.25944,-0.33944\
O,0,-2.94505,-1.90791,0.66255\C,0,-3.4432,-0.80615,0.7518\O,0,-4.10653
,-0.37316,1.83134\C,0,-4.17326,-1.29126,2.94562\C,0,-4.96623,-0.61914,
4.0434\O,0,-4.29672,-0.93223,-2.15544\C,0,-4.19235,-1.71315,-3.36569\C
,0,-5.59384,-2.10593,-3.77516\H,0,-5.55674,-2.7001,-4.69429\H,0,-6.076
16,-2.70565,-2.99708\H,0,-6.2099,-1.221,-3.96269\H,0,-3.69241,-1.10749
,-4.12819\H,0,-3.56073,-2.5838,-3.16232\H,0,-4.40334,0.69849,-0.40355\
H,0,-3.15238,-1.52929,3.26085\H,0,-4.64207,-2.21883,2.60228\H,0,-5.035
2,-1.28671,4.90876\H,0,-4.48363,0.30934,4.36346\H,0,-5.98172,-0.38585,
3.70814\C,0,-4.66248,4.16594,1.26101\C,0,-4.13662,2.90045,1.00357\C,0,
-2.99623,2.74962,0.207\C,0,-4.05316,5.30212,0.7329\C,0,-2.90775,5.1629
2,-0.04959\C,0,-2.38413,3.89885,-0.30916\H,0,-4.61531,2.0266,1.43778\H
,0,-5.55178,4.26202,1.87972\H,0,-4.46528,6.28818,0.93223\H,0,-2.41938,
6.04219,-0.46295\H,0,-1.47802,3.79291,-0.89965\C,0,-2.35288,1.39387,-0
.0798\C,0,-1.35578,1.02889,0.97621\H,0,-1.7776,1.5024,-1.00384\N,0,-0.
07135,1.28535,0.84687\O,0,0.73809,0.92435,1.79596\O,0,0.40968,1.88642,
-0.19337\H,0,-1.64336,0.6344,1.9409\\Version=AM64L-G03RevD.02\State=1-
A\HF=-2385.0401121\RMSD=2.961e-09\Thermal=0.\Dipole=-2.2924842,-1.0459
993,-0.9220885\PG=C01 [X(C31H44N6O6S1)]\\@

INIT11

1\1\GINC-A08\FOpt\RB3PW91\6-31G(d)\C31H44N6O6S1\SHUBINA\05-Aug-2008\0\
\#P B3PW91/6-31G(d) Opt Freq=NoRaman Name=SHUBINA\\Init TS9R new\\0,1\
S,4.9518865433,-0.3176029542,-0.8939225045\N,2.5197905559,0.80132918,-
1.2775414734\N,2.616651676,-1.4377720469,-1.6241306744\N,1.0347486657,
0.4146799363,1.891754985\N,3.0128385435,-0.7867595145,1.955051935\N,3.
0781486025,1.4630429375,1.4412136642\C,2.9812838242,2.1290511372,-0.93
23811871\C,2.6090128766,2.5013577101,0.5214725222\C,3.1840715142,3.869
1173266,0.8921740566\C,2.6836001758,4.9428385452,-0.0781761831\C,2.993
9315234,4.5771351517,-1.5321025118\C,2.4479976711,3.1928043977,-1.8974
842819\C,3.2885515447,-0.3189012035,-1.2878713855\C,3.169411593,-2.784
2441107,-1.6519695902\C,2.4864804277,-3.5727687349,-2.7739528685\C,3.0
387703176,-3.5055679875,-0.317017735\C,1.8479628556,-3.4564643843,0.41
84753891\C,4.104488875,-4.2627443527,0.1772953962\C,1.7306648804,-4.14
9872757,1.6234254511\C,3.9871282809,-4.9619677891,1.3787433242\C,2.799
406764,-4.9076834811,2.1059405641\C,2.3455836683,0.3619651567,1.730029
9862\H,2.9049197361,4.1119893976,1.9240135649\H,1.5183234883,2.5607054
434,0.5994639085\H,4.0750031801,2.0966659596,-1.0046730403\H,1.5985007
059,5.056361656,0.0447239481\H,3.134972326,5.909943668,0.1749164611\H,
2.5749419379,5.3317560071,-2.2088046694\H,4.0824707215,4.5869558187,-1
.688512876\H,1.3492642775,3.2085802341,-1.8560386991\H,2.7313056726,2.
9200264376,-2.9206444278\H,4.2829315482,3.8186839354,0.8610081571\H,1.
5102179109,0.6473513456,-1.3562326599\H,1.5979767432,-1.3478780751,-1.
6732632478\H,4.2333264826,-2.6759779529,-1.8816613357\H,2.9026087016,-
4.5833916944,-2.8309829505\H,1.4098126451,-3.6673079029,-2.5891024863\
H,2.6345074937,-3.0757022105,-3.7386769984\H,1.0115791783,-2.847445920
1,0.0832949301\H,5.0401155466,-4.2946605229,-0.3773849546\H,0.79892365
37,-4.0815655802,2.1786074558\H,4.8294866987,-5.540253695,1.7507564657
\H,2.7081560977,-5.4479423773,3.0451049372\H,4.0683674094,1.2580921909
,1.3345316314\H,3.8439120412,-0.9432562784,1.3841638977\H,2.4611745406
,-1.620841442,2.1210005763\H,0.444595569,-0.4313011441,1.7616369681\O,
-0.7078257881,-1.6910245308,1.6279222418\C,-1.8254000904,-1.5093437951
,1.1055574187\C,-2.1688720329,-0.9988503843,-0.1632910295\O,0.00063706
72,-0.5352574901,-1.0751494764\C,-1.2479340031,-0.5835284782,-1.142959
0647\O,-1.8812079806,-0.1870577752,-2.2933786619\C,-1.054026923,0.2265
099798,-3.3743571323\C,-1.9612488457,0.5327586501,-4.5491925665\O,-2.9
522383461,-1.8384361564,1.8326800322\C,-2.7199959527,-2.4137896025,3.1
177698098\C,-4.0693114308,-2.6657821751,3.7598165923\H,-3.9374553815,-
3.1244684091,4.7460917759\H,-4.6234775925,-1.7300760905,3.8911096452\H
,-4.6741386136,-3.3406191262,3.1451224012\H,-2.1553088792,-3.347084560
4,3.0054105107\H,-2.106380642,-1.7374545444,3.7241147107\H,-3.21631527
26,-0.989037916,-0.4358598403\H,-0.3367467168,-0.5650554456,-3.6241896
231\H,-0.4725905354,1.1126199715,-3.0868283352\H,-1.3685035051,0.86093
87413,-5.4102008147\H,-2.5318936799,-0.3551844266,-4.8395328721\H,-2.6
712826666,1.3265022304,-4.2953051613\H,0.5414350568,1.294709508,1.7879
31466\C,-7.0254434471,0.1774960668,0.7417123214\C,-5.6786901815,0.4596

675161,0.9449835897\C,-5.1530503712,1.7165143209,0.5959920347\C,-7.868
2564751,1.146931485,0.2004945539\C,-7.3599812035,2.4032338533,-0.14041
22652\C,-6.0153868295,2.6869942105,0.0534757924\H,-5.0144499422,-0.297
7734088,1.3549723977\H,-7.4172081777,-0.800597047,1.0069263708\H,-8.92
17680187,0.9284232786,0.0463435811\H,-8.0179450884,3.1624477575,-0.554
5740574\H,-5.6347621266,3.6725918483,-0.2004147859\C,-3.736556145,1.95
2411446,0.8231686232\C,-3.0139042344,2.9797387868,0.3414857398\H,-3.20
45526592,1.2122248727,1.4148881611\N,-1.6156190402,3.0725779185,0.6258
720922\O,-0.9684610961,3.8876474319,-0.032751267\O,-1.1284132206,2.348
2027839,1.5000441905\H,-3.3434486738,3.7547942436,-0.3362555346\\Versi
on=IA64L-G03RevD.01\State=1-A\HF=-2385.0289083\RMSD=5.773e-09\RMSF=4.1
33e-06\Thermal=0.\Dipole=-1.7277952,1.0054263,0.3095872\PG=C01 [X(C31H
44N6O6S1)]\\@

TS11

1\1\FAU-CCC-CCDH152\Freq\RB3PW91\6-31G(d)\C31H44N6O6S1\SCHENKER\20-Jul
-2008\0\\#P Geom=AllCheck Guess=Read SCRF=Check GenChk RB3PW91/6-31G(d
) Freq\\tsr4n\\0,1\S,4.7036946893,-0.1329219915,-1.2187720184\N,2.4743
665413,1.3782221413,-1.1023558845\N,2.1575504094,-0.789326669,-1.75522
74368\N,1.2756411661,0.3123870681,1.9870100678\N,3.2208591294,-0.94539
95608,1.8160604975\N,3.3446126066,1.3453258154,1.6292940764\C,3.183083
6705,2.5092352199,-0.5308892155\C,2.9181386521,2.5926244649,0.99099452
79\C,3.6523512601,3.7808084804,1.6150481002\C,3.2839978351,5.086935348
3,0.907876126\C,3.5601997916,5.0022853764,-0.5950389275\C,2.8208497276
,3.8225413793,-1.2316138102\C,3.0256669564,0.1682755397,-1.3664897003\
C,2.5371299608,-2.1586973112,-2.0962798703\C,1.5803149544,-2.689979533
2,-3.1655277597\C,2.5816552212,-3.0763867407,-0.8820521444\C,1.4891010
968,-3.1715739869,-0.0111395674\C,3.7140501594,-3.8555361874,-0.630182
6838\C,1.533381364,-4.0280323022,1.0895539775\C,3.7580356121,-4.717669
9573,0.4663417561\C,2.668222384,-4.8060388263,1.3306923837\C,2.5785980
423,0.2463335429,1.7788832847\H,3.4165404911,3.8304612188,2.6845540732
\H,1.8411562681,2.7167060752,1.141283961\H,4.249719145,2.3060936408,-0
.6823724338\H,2.2180152974,5.2985958967,1.0711028735\H,3.8422265018,5.
9186598022,1.3547602859\H,3.2623522656,5.9355048535,-1.0883136373\H,4.
6420711561,4.8923906587,-0.7607041809\H,1.7385686653,3.9843284895,-1.1
664169059\H,3.075195961,3.7326457764,-2.2943469457\H,4.7379865907,3.61
54554423,1.5359141826\H,1.45899236,1.4831831326,-1.1223715493\H,1.1557
368999,-0.6091567107,-1.6812755815\H,3.5460522078,-2.1000908931,-2.513
732706\H,1.8674472772,-3.7065048139,-3.4516754382\H,0.5527927992,-2.72
18095826,-2.7885827089\H,1.6084692857,-2.0528471756,-4.0562249536\H,0.
6061347323,-2.5566676053,-0.1676647594\H,4.5749602088,-3.7736977467,-1
.2898434513\H,0.6758238857,-4.0775642408,1.7555773073\H,4.649644467,-5
.312508934,0.6493574161\H,2.7026518384,-5.4742765605,2.1877226028\H,4.
3225444885,1.1257665261,1.4701989877\H,3.9820028359,-1.0417020212,1.14
25867617\H,2.6367796917,-1.7732833237,1.8649854615\H,0.6799148564,-0.5
130629163,1.8781839946\O,-0.839199767,-1.5440766728,1.7223920308\C,-2.
0328579384,-1.3233223394,1.5187650475\C,-2.7003532787,-0.996893346,0.2
777786501\O,-0.7919826079,-0.7710232296,-1.1554731157\C,-1.9901021519,
-0.9860782857,-0.9772268792\O,-2.8495955229,-1.1342655521,-2.018676299
2\C,-2.2904034017,-1.026622393,-3.331620013\C,-3.4278764463,-1.1547458
022,-4.3227702783\O,-2.9318267243,-1.3718246346,2.5394893142\C,-2.3978
295539,-1.6793293547,3.8335814171\C,-3.5485239937,-1.6664758191,4.8172
402844\H,-3.1843417369,-1.9084051219,5.8216067707\H,-4.0217375774,-0.6
798244379,4.8536235396\H,-4.3085969433,-2.4037993431,4.5396568167\H,-1
.9079343459,-2.6589771357,3.797298732\H,-1.6329380496,-0.93945904,4.09
39541599\H,-3.741950707,-1.2872553318,0.2284269366\H,-1.546772726,-1.8
184510211,-3.4756912958\H,-1.7683358197,-0.0678901388,-3.429125884\H,-
3.0444730888,-1.0771303315,-5.3459758628\H,-3.9303347318,-2.121146525,
-4.214676116\H,-4.1709052139,-0.3658872565,-4.1695396152\H,0.741804474
,1.1870104723,1.9310181379\C,-6.9080037642,0.7255193479,0.5396099394\C
,-5.5642086447,0.7436228906,0.8954801818\C,-4.579315952,1.1579897755,-
0.0160278241\C,-7.2978324895,1.1206266577,-0.7401109216\C,-6.331101095
3,1.531858252,-1.6568551245\C,-4.9862275715,1.5503235256,-1.2999616195
\H,-5.2592422047,0.4209266582,1.8879521907\H,-7.6533960943,0.404310312
1,1.2627173762\H,-8.3478994482,1.1095892594,-1.0205908826\H,-6.6262385
549,1.844943881,-2.6552098467\H,-4.251109021,1.8825526014,-2.026181031
7\C,-3.1740007827,1.1945306782,0.4290459439\C,-2.1966180894,1.91147034

63,-0.259721111\H,-3.0280236615,1.1296170622,1.5020769212\N,-0.9992156
466,2.205054062,0.330370951\O,-0.0762997526,2.6806648033,-0.38175865\O
,-0.8332436919,2.0216898424,1.567877146\H,-2.2228358576,2.1562465026,-
1.3108800335\\Version=AM64L-G03RevD.02\State=1-A\HF=-2385.0156821\RMSD
=7.393e-09\RMSF=1.531e-06\ZeroPoint=0.7432892\Thermal=0.7885256\ZPE=46
6.4210421\Dipole=-0.7217463,-0.2247729,0.3817263\DipoleDeriv=-0.924750
475684,-0.0074971\Polar=520.725381,-1.8780735,404.5269328,3.2122571,-3
3.8744691,375.561958\PG=C01 [X(C31H44N6O6S1)]\NImag=1\\0.27795675,-0.0
1453479,-0.07927342,0.36263449\\0.00000061,0.00000050,0.,-0.00000029,-
0.00000085,-0.00000176,-0.00000109\\\@

FIN 11

1\1\GINC-A06\FOpt\RB3PW91\6-31G(d)\C31H44N6O6S1\SHUBINA\05-Aug-2008\0\
\#P B3PW91/6-31G(d) Opt Freq=NoRaman Name=SHUBINA\\TS4R fin\\0,1\S,4.8
416070236,0.0898582359,-1.0473152586\N,2.57686416,1.545295861,-0.85585
12929\N,2.3267845905,-0.5251805752,-1.7668175428\N,1.3439509588,-0.126
3882934,1.8406403071\N,3.4560774288,-1.1093177225,1.8911272364\N,3.284
9500855,1.1809176709,1.9172141378\C,3.200709644,2.6046628169,-0.086966
1678\C,2.7962132784,2.4605833285,1.4004255927\C,3.3578834862,3.6013733
249,2.2520480103\C,2.9564475536,4.9654488111,1.6879737082\C,3.38995716
19,5.1065913694,0.2275944702\C,2.8095604883,3.982544311,-0.6328723571\
C,3.167163935,0.3928118834,-1.2264705973\C,2.7125126292,-1.8634310902,
-2.2056310067\C,1.7802306003,-2.3012658379,-3.3365984409\C,2.728693143
1,-2.8625809228,-1.0550910324\C,1.572628125,-3.1107303438,-0.305269450
3\C,3.9056194771,-3.5385160081,-0.7202961634\C,1.5990523676,-4.0064297
689,0.7648475103\C,3.933806255,-4.4402406533,0.3449457788\C,2.78141202
6,-4.6741518812,1.0948121723\C,2.6596512747,-0.0061189399,1.8478987062
\H,3.0127978487,3.4837775946,3.2861396034\H,1.7018624031,2.4678947551,
1.4526306005\H,4.28527421,2.4681231452,-0.1734169675\H,1.8658849378,5.
0801267463,1.7574267122\H,3.397793267,5.7615093271,2.3002503717\H,3.07
23162368,6.0783240273,-0.1698402104\H,4.4886232142,5.0894438617,0.1702
974923\H,1.7155916128,4.0567530361,-0.6554864526\H,3.1644926303,4.0601
622364,-1.6673268965\H,4.4568290485,3.5273703642,2.2727280975\H,1.5464
516014,1.6358434714,-0.8761749651\H,1.3306928903,-0.3241031788,-1.7598
360849\H,3.7327512951,-1.7754748458,-2.5894832519\H,2.0459294564,-3.30
87823234,-3.6714376119\H,0.7347586609,-2.3156440652,-3.0085980175\H,1.
8655659267,-1.6151846776,-4.1859383645\H,0.6446671266,-2.5924456538,-0
.532819305\H,4.8125615694,-3.3382481731,-1.2853531036\H,0.6903422486,-
4.1772240071,1.3363167497\H,4.8601072942,-4.9519089054,0.5949595315\H,
2.8026543654,-5.3721443358,1.9283609767\H,4.2951648395,1.0991328321,1.
923910169\H,4.2658084722,-1.0465764868,1.272156292\H,2.9775782568,-1.9
985486585,1.7918190639\H,0.9371314834,-1.028420147,1.6135557709\O,-0.9
621167703,-1.8071967978,1.0924795988\C,-2.0077083151,-1.1972145535,1.2
138529938\C,-2.9169766496,-0.7268379497,0.0920090396\O,-1.294837039,-0
.9921045622,-1.7057013574\C,-2.4170451621,-1.1497765208,-1.272172032\O
,-3.4213155173,-1.6824575941,-1.989320307\C,-3.1238250849,-2.025333226
2,-3.3559805893\C,-4.4271925583,-2.4151297658,-4.0185142182\O,-2.58532
42714,-0.9553054364,2.3957498154\C,-1.8089688674,-1.3001112939,3.55755
04992\C,-2.6434849762,-0.9847402734,4.7778121856\H,-2.0799967153,-1.23
40929537,5.6833688258\H,-2.8953868522,0.0794935984,4.8115250283\H,-3.5
722620347,-1.5639882781,4.7798986364\H,-1.5447301054,-2.3615873128,3.5
024544362\H,-0.8839149249,-0.7161109333,3.5366324951\H,-3.8866945811,-
1.2038619576,0.2674773111\H,-2.3969732694,-2.8449254289,-3.3629599491\
H,-2.6550811156,-1.1634839516,-3.8420706129\H,-4.2494210864,-2.6722552
637,-5.0682331576\H,-4.8762167217,-3.2815291069,-3.5229320997\H,-5.143
082884,-1.5883703062,-3.9799571293\H,0.6557032121,0.6562653147,1.70550
19978\C,-6.9334953577,1.4923705254,0.0271940283\C,-5.6459742149,1.2229
647363,0.4845838782\C,-4.5587236608,1.1701062252,-0.3962310262\C,-7.15
83859178,1.71728876,-1.3296420055\C,-6.0861000933,1.6682185619,-2.2183
735591\C,-4.7994232833,1.3947577709,-1.7561433742\H,-5.4756014685,1.05
2660886,1.545867443\H,-7.7596369426,1.5331960747,0.7329511994\H,-8.160
733788,1.9328394155,-1.6909249702\H,-6.2499733304,1.8480446074,-3.2782
344127\H,-3.9784726187,1.3625744876,-2.4676382495\C,-3.1838971838,0.84
92397037,0.1617440856\C,-2.0921110772,1.6570263487,-0.4436219877\H,-3.
1998623751,1.056743748,1.2364969415\N,-0.9537864855,1.8152367675,0.195
7118563\O,0.0291247701,2.438942302,-0.3461448643\O,-0.8167205347,1.348
1609366,1.4006359751\H,-2.1308067364,2.0903441809,-1.4318138882\\Versi

on=IA64L-G03RevD.01\State=1-A\HF=-2385.0345459\RMSD=4.508e-09\RMSF=7.359e-05\Thermal=0.\Dipole=-0.934896,-1.264467,0.7050538\PG=C01 [X(C31H44N6O6S1)]\\@

Init 12

1\1\GINC-A04\FOpt\RB3PW91\6-31G(d)\C31H44N6O6S1\SHUBINA\10-Aug-2008\0\\#P B3PW91/6-31G(d) Opt Freq=NoRaman Name=SHUBINA\\Init TS 11S\\0,1\S,0.6351225666,4.5770918315,-1.8026947508\N,-0.6846796931,2.2124106132,-1.8360020616\N,1.5769325014,2.0703611888,-2.0243345479\N,-0.6804852505,1.5452427655,1.5542263146\N,0.5531838947,3.4942804525,1.7858720348\N,-1.3933929653,3.5400174128,0.5481136644\C,-1.9748629658,2.8600990493,-1.7387749865\C,-2.4552777329,2.9609989393,-0.2720398093\C,-3.7398875885,3.7884210227,-0.1991908838\C,-4.8272901692,3.1388074313,-1.0620920774\C,-4.3647706406,2.9355028125,-2.5083044758\C,-3.0425194373,2.1640794447,-2.5856167335\C,0.4965543597,2.8708725802,-1.8898638163\C,2.9547716653,2.5196768199,-1.8988873527\C,3.8631151886,1.5794305763,-2.6946586892\C,3.4005639867,2.6246626811,-0.4456469689\C,3.1176208281,1.6015804531,0.4664242017\C,4.119770417,3.7383818583,-0.0037810721\C,3.5478323931,1.6899671779,1.7910942224\C,4.5529579016,3.8308751762,1.319239146\C,4.2680884327,2.8070438458,2.2219223239\C,-0.5068655651,2.8278089164,1.261431411\H,-4.0665541181,3.8694191035,0.8443393174\H,-2.6722475147,1.9561527946,0.0960974752\H,-1.8314601665,3.8809264606,-2.1140745885\H,-5.0920877535,2.1651135317,-0.6261832758\H,-5.737434489,3.7507503238,-1.0369826892\H,-5.1385737109,2.4078089353,-3.0793661415\H,-4.2377071432,3.9166001956,-2.9888211709\H,-3.1770523808,1.1373251174,-2.2213244295\H,-2.6938571636,2.101108477,-3.6230680848\H,-3.5396466177,4.810555001,-0.5536961806\H,-0.6481560615,1.1900205386,-1.7261196673\H,1.3992978669,1.0632367165,-2.0075139036\H,3.0004266509,3.5189931132,-2.3414404345\H,4.9004280494,1.9241399996,-2.6413368788\H,3.8306792943,0.5608521505,-2.2908827844\H,3.555915447,1.5490252764,-3.745451554\H,2.5358209458,0.7374661523,0.1561019185\H,4.327704814,4.548480615,-0.6994913958\H,3.3110335339,0.8825886249,2.4790787078\H,5.1059329334,4.7081999341,1.6462392842\H,4.6033193436,2.8775387772,3.2538272062\H,-0.9851523725,4.3742053859,0.1216108769\H,0.7794099913,4.3744722239,1.3375675601\H,1.3819158973,2.9365771622,1.9671691953\H,-0.0072617678,1.0767860599,2.1544888879\O,0.3327495355,-0.4599135418,-1.5973189822\C,0.0199456265,-1.6428643097,-1.361996181\C,-1.1360688631,-2.1596947332,-0.7462087194\O,-2.2097115101,-0.1296472863,-0.0458517399\C,-2.1533029699,-1.373552211,-0.1779057205\O,-3.1928997265,-2.1385321614,0.2820491515\C,-4.2696202999,-1.4421081387,0.9012463573\C,-5.2614791204,-2.4807027933,1.3855618261\O,0.880715864,-2.6562068502,-1.7339475544\C,2.0497719972,-2.2685181691,-2.449625006\C,2.7382415514,-3.5320033526,-2.9257996293\H,3.6449948549,-3.2800482012,-3.4868633281\H,3.0259072481,-4.1656352295,-2.0799393068\H,2.0775975541,-4.1103823493,-3.5798467707\H,1.7714042233,-1.6257759243,-3.2926360516\H,2.7086262837,-1.6853458432,-1.7936406851\H,-1.2733420979,-3.2331236855,-0.7426887995\H,-4.7333410922,-0.7569144649,0.1804350208\H,-3.8934070907,-0.8309197584,1.7308026998\H,-6.1156053493,-1.9932110911,1.8689000683\H,-5.6340980125,-3.0818196181,0.5498029517\H,-4.7943028796,-3.1566489942,2.1092758343\H,-1.2509139821,0.9205709176,0.9573494872\C,-0.2292781231,-7.1026127969,0.5947564618\C,0.1909304262,-5.7774597033,0.6286768398\C,0.1809894028,-5.0537849243,1.8340752878\C,-0.6560203074,-7.7276701526,1.7654085072\C,-0.6628866019,-7.0223447171,2.9718080076\C,-0.2494630839,-5.6979746946,3.0081584689\H,0.5154179979,-5.278937121,-0.2815047935\H,-0.2238066531,-7.6475702151,-0.3451516797\H,-0.9814540529,-8.7643998961,1.7418422624\H,-0.9896773658,-7.5112065309,3.8856321773\H,-0.2471741855,-5.1637018789,3.9543195719\C,0.6260209597,-3.6707904921,1.7990047341\C,0.5044661586,-2.7616705986,2.782587785\H,1.0791628563,-3.3276888757,0.8718736968\N,0.9590788689,-1.4238764512,2.5611200794\O,0.5670166994,-0.5660989746,3.3664746141\O,1.687388831,-1.1766584729,1.6041935374\H,0.002384793,-2.8752084348,3.7331431759\\Version=IA64L-G03RevD.01\State=1-A\HF=-2385.0275438\RMSD=8.356e-09\RMSF=7.026e-05\Thermal=0.\Dipole=0.0006838,-1.5009302,1.3659171\PG=C01 [X(C31H44N6O6S1)]\\@

TS12

1\1\GINC-A05\FTS\RB3PW91\6-31G(d)\C31H44N6O6S1\SHUBINA\10-Aug-2008\0\\

#P B3PW91/6-31G* OPT=(TS,ReadFc,noeigen) Freq=NoRaman Name=SHUBINA\\TS
5S\\0,1\S,-4.3591615233,1.5110301113,1.143316106\N,-1.6630413566,1.709
4848394,1.1415847163\N,-2.4956770848,-0.2441893069,1.9577440511\N,-0.9
403643337,0.1597999481,-1.8939392295\N,-3.2224120544,-0.2198381305,-1.
8686296529\N,-2.4470110774,1.9316328868,-1.5634790755\C,-1.6919804068,
3.0108040137,0.5126132543\C,-1.5019070145,2.9021900534,-1.0186395295\C
,-1.6661160043,4.2759345818,-1.6713457656\C,-0.6547408019,5.2646521575
,-1.0820586531\C,-0.7585365902,5.3449015813,0.4439732181\C,-0.64829132
88,3.963881712,1.0991026296\C,-2.7629829583,0.9668429702,1.4274003037\
C,-3.5011272094,-1.2387524378,2.3193253344\C,-2.9717561557,-2.08532049
55,3.4782289171\C,-3.9270804057,-2.1042863375,1.1410560219\C,-2.986942
972,-2.8103531181,0.3809210925\C,-5.2815188743,-2.2188710154,0.8141629
657\C,-3.3967020953,-3.6131774298,-0.6841818301\C,-5.6946052508,-3.027
357832,-0.245064934\C,-4.7525185817,-3.7264906889,-0.9981790076\C,-2.1
725511687,0.6242824396,-1.7376416871\H,-1.5371716427,4.1839769472,-2.7
560779836\H,-0.4868309879,2.5493886478,-1.2214286055\H,-2.692873759,3.
4178574426,0.7006129472\H,0.3596631007,4.9446145652,-1.3607175157\H,-0
.8017229051,6.2566431817,-1.5261320534\H,0.0187427102,6.0094962185,0.8
405186561\H,-1.7231724761,5.7955868787,0.7183617713\H,0.3564965362,3.5
488636724,0.936237149\H,-0.7964406345,4.0369712877,2.1826571692\H,-2.6
896335151,4.6410373933,-1.5002447204\H,-0.7547793338,1.2430737915,1.21
78047405\H,-1.5133219925,-0.5119222767,2.0225232308\H,-4.3762792227,-0
.6772259304,2.6593968082\H,-3.736643274,-2.8010501234,3.7942400563\H,-
2.0868994607,-2.6597003593,3.1808795897\H,-2.7106356642,-1.4512662085,
4.3326347296\H,-1.9217922695,-2.7373663403,0.5898974456\H,-6.016271144
1,-1.6571898888,1.3866343114\H,-2.6418939555,-4.1359040354,-1.26451148
88\H,-6.7523376242,-3.1025443745,-0.4861218194\H,-5.0713798456,-4.3539
270608,-1.8270457854\H,-3.4029671109,2.0997152541,-1.2531241093\H,-4.0
712366519,0.0520663724,-1.3820668489\H,-3.0206933973,-1.2098349351,-1.
77187504\H,-0.8061560802,-0.8620230985,-1.9316319961\O,0.4172151151,-0
.2184719326,1.5450788761\C,1.6160499776,-0.5036041559,1.6096621335\C,2
.6787717876,-0.0591483019,0.7365596172\O,1.4458240229,1.3479228916,-0.
7758663399\C,2.4822700512,1.0356184248,-0.1819811573\O,3.6402153081,1.
6992274103,-0.407314774\C,3.6034845202,2.7373063091,-1.3932132127\C,5.
0055011899,3.2944892056,-1.5164119472\O,2.0987349263,-1.339669602,2.55
45305122\C,1.1349170129,-1.9826559751,3.3980557227\C,1.8954433314,-2.8
521017527,4.3761816524\H,1.1936417736,-3.365010296,5.0425615799\H,2.48
20099535,-3.6096451567,3.8471752871\H,2.5757979702,-2.2514067301,4.988
4834282\H,0.540258523,-1.2200239611,3.9136100631\H,0.4651524452,-2.574
7184146,2.765534303\H,3.6737423525,-0.1323048047,1.1601385286\H,2.8879
736591,3.5066649066,-1.080254414\H,3.244997764,2.3229898215,-2.3419342
622\H,5.0261881181,4.095985678,-2.263005427\H,5.3472957746,3.704742264
7,-0.560822124\H,5.7058435279,2.5125944746,-1.8251417063\H,-0.12567251
45,0.6750878994,-1.55440801\C,6.6085761122,-1.7069773118,-1.0203814487
\C,5.3391273757,-1.9114178302,-0.4896464845\C,4.1892044497,-1.51356605
49,-1.1881979008\C,6.7552417128,-1.0946096198,-2.2642878949\C,5.621545
9062,-0.689212521,-2.9688489675\C,4.3523121422,-0.8941265865,-2.436345
6629\H,5.2288693523,-2.3924033121,0.479848895\H,7.4845605155,-2.030295
6984,-0.4639170937\H,7.7454608871,-0.9371082478,-2.6838861525\H,5.7258
529015,-0.2155268773,-3.9418736698\H,3.4819895704,-0.5710985293,-3.000
4918647\C,2.864755774,-1.7965005579,-0.5977402022\C,1.7329597147,-1.93
84882454,-1.3984459882\H,2.8785583938,-2.4236765091,0.2893088756\N,0.6
071482791,-2.5741405641,-0.9292518457\O,-0.4132689415,-2.5897019276,-1
.6862825034\O,0.5905486604,-3.1313650828,0.1842593765\H,1.6257494527,-
1.5505232511,-2.4001656806\\Version=IA64L-G03RevD.01\State=1-A\HF=-238
5.019039\RMSD=3.571e-09\RMSF=4.430e-06\Thermal=0.\Dipole=1.2117694,1.3
593675,-0.4015805\PG=C01 [X(C31H44N6O6S1)]\\@

Fin 12

1\1\GINC-A05\FOpt\RB3PW91\6-31G(d)\C31H44N6O6S1\SHUBINA\11-Aug-2008\0\
\#P B3PW91/6-31G(d) Opt Freq=NoRaman Name=SHUBINA\\Fin S 12\\0,1\S,0.9
491436576,4.2938070372,-1.4388797263\N,-0.3350941716,1.9241871187,-1.3
943584675\N,1.9245323564,1.8088396256,-1.1138814793\N,-0.6127101219,1.
2713803693,1.9884677365\N,0.6412280994,3.2038837986,1.8359413227\N,-1.
4721044468,3.1754334728,0.9049253248\C,-1.6365570499,2.5533663847,-1.4
72812344\C,-2.3363650185,2.5398461825,-0.0934507179\C,-3.6914722643,3.
2460094083,-0.1573798398\C,-4.5824763881,2.6267473056,-1.2370222986\C,

-3.8893225782,2.6247593359,-2.6013977247\C,-2.5314953894,1.9197200311,
-2.5419842274\C,0.8488751522,2.5893582811,-1.3184885972\C,3.3076752694
,2.2783340529,-1.180610271\C,4.1802148655,1.1213220646,-1.6773805637\C
,3.8387173067,2.8492190705,0.1286289743\C,3.8529094182,2.0849255594,1.
3023907932\C,4.3763791715,4.138866708,0.1566219937\C,4.392796616,2.610
0056601,2.4770319438\C,4.9232127954,4.6603686851,1.3291257798\C,4.9331
682106,3.8967346871,2.494421548\C,-0.4805207944,2.5134629051,1.5462500
676\H,-4.172713054,3.2022039273,0.8268114516\H,-2.4958150224,1.4977730
859,0.205938869\H,-1.4491189164,3.6001252388,-1.742108047\H,-4.8292822
329,1.5930930118,-0.9529314398\H,-5.5334281426,3.1708122343,-1.2889914
414\H,-4.5282786632,2.1395534074,-3.3494730079\H,-3.7485326152,3.66226
99737,-2.9376696603\H,-2.6667881194,0.853390729,-2.3238588624\H,-2.019
9428614,1.9820013188,-3.5093536555\H,-3.5287870746,4.3105695303,-0.383
6348437\H,-0.3221355822,0.9045060256,-1.4045802448\H,1.7750811067,0.83
84763062,-0.7843961125\H,3.3228984346,3.0847283224,-1.9201664298\H,5.2
27906667,1.4338920105,-1.7252812899\H,4.109173013,0.2595156566,-1.0051
555192\H,3.8625935789,0.8049259252,-2.6772794342\H,3.4083237874,1.0912
018661,1.3145006316\H,4.3523429036,4.7450880468,-0.7460384525\H,4.3974
671382,2.0039373221,3.3804270782\H,5.3341309126,5.6672334302,1.3316547
391\H,5.3569774244,4.3010377678,3.4106390429\H,-1.2016960244,4.1193322
535,0.6428314171\H,0.9490622668,3.871304466,1.126430017\H,1.4045200163
,2.6792788669,2.2486174857\H,0.2758465509,0.7180296623,2.0997550914\O,
-1.0378469914,-0.9160422892,-1.8749127347\C,-0.5647734782,-1.987697644
,-1.5443432691\C,-1.016126174,-2.8174809299,-0.3519977742\O,-2.1824314
63,-1.1464299625,0.9931859901\C,-2.2039445169,-2.1581784718,0.31991648
18\O,-3.3244933855,-2.8540090985,0.0835890251\C,-4.5334966387,-2.33718
45855,0.67189136\C,-5.6509884761,-3.298990878,0.3351328607\O,0.3178930
775,-2.6711353674,-2.2722851378\C,0.8843776321,-1.974342085,-3.3991613
877\C,1.8755687463,-2.9071337277,-4.057819048\H,2.3306064703,-2.412025
4429,-4.9222550285\H,2.6721512521,-3.1819225736,-3.3597372817\H,1.3852
072365,-3.8223480024,-4.4050529799\H,0.0743922952,-1.6853613135,-4.076
896904\H,1.3629961841,-1.0632685322,-3.029031858\H,-1.362566059,-3.767
0083867,-0.7743822389\H,-4.7173477845,-1.3349328139,0.2700658335\H,-4.
3848207565,-2.2391957257,1.7522171757\H,-6.5914618007,-2.9400489626,0.
7664932939\H,-5.7800963185,-3.3861181175,-0.7481537582\H,-5.4434929765
,-4.2943888852,0.7397153174\H,-1.4280986251,0.7217445817,1.7449382547\
C,-0.0862235656,-6.8092517465,1.8134214514\C,0.2766714393,-5.670773644
1,1.0961458326\C,-0.2221936746,-4.4115933989,1.4439084436\C,-0.9589591
474,-6.7056819841,2.8947073232\C,-1.4635522938,-5.4563039219,3.2521337
508\C,-1.0987485345,-4.3202571333,2.5326139749\H,0.9625239582,-5.75904
15639,0.2560425634\H,0.3184317484,-7.7774875739,1.5291383811\H,-1.2409
634509,-7.5915022637,3.4580870333\H,-2.1412188821,-5.3638624394,4.0974
698363\H,-1.4930784224,-3.3499002952,2.8261707074\C,0.1648403217,-3.18
9289814,0.6291081369\C,0.6417849665,-2.0888691524,1.5139978807\H,0.977
9694259,-3.470641269,-0.0525848473\N,1.2647839282,-1.0370424252,1.0361
658332\O,1.6668684813,-0.1022760079,1.8449452037\O,1.4708505673,-0.900
5036601,-0.2299337637\H,0.5481214978,-2.1239656224,2.5892744569\\Versi
on=IA64L-G03RevD.01\State=1-A\HF=-2385.0420363\RMSD=3.633e-09\RMSF=4.8
89e-06\Thermal=0.\Dipole=-2.3453075,-1.279408,-0.1344498\PG=C01 [X(C31
H44N6O6S1)]\\@

3-TABDa

\0\\#P B3PW91/6-31G(d) SCF=Tight Name=SHUBINA SCRF=(PCM,Solvent=THF)\\
Catalyst1\\0,1\C,0,4.38453,-0.10633,0.05509\C,0,2.89478,-0.05048,-0.30
81\C,0,2.21958,-1.41577,-0.06976\C,0,2.95213,-2.52798,-0.8328\C,0,4.43
979,-2.57105,-0.48169\C,0,5.09974,-1.21296,-0.72581\N,0,0.82973,-1.309
19,-0.47988\C,0,-0.21195,-1.89839,0.14602\S,0,-0.04004,-3.00649,1.4120
1\N,0,-1.42782,-1.50602,-0.33021\C,0,-2.71528,-2.07181,0.05621\C,0,-3.
73801,-0.94696,0.13203\C,0,-3.09438,-3.2467,-0.84405\C,0,-3.54778,0.06
901,1.07991\C,0,-4.44964,1.12164,1.19257\C,0,-5.56365,1.18223,0.35275\
C,0,-5.76335,0.18089,-0.59242\C,0,-4.8568,-0.87606,-0.701\N,0,2.20357,
1.05996,0.35461\C,0,1.27429,1.7997,-0.36336\N,0,0.81752,2.96544,0.2354
9\C,0,0.90598,3.21144,1.66654\C,0,2.11835,2.53458,2.27788\C,0,2.15604,
1.09024,1.80973\C,0,-0.26253,3.67357,-0.43506\C,0,-0.02056,3.66855,-1.
93471\C,0,0.09386,2.21998,-2.39686\N,0,0.93333,1.39573,-1.55007\H,0,4.
51603,-0.30467,1.12813\H,0,4.83631,0.87229,-0.14771\H,0,2.82131,0.1741
,-1.37739\H,0,2.23317,-1.66095,1.00117\H,0,2.83087,-2.34926,-1.91127\H

,0,2.46289,-3.48168,-0.60619\H,0,4.94177,-3.35173,-1.06672\H,0,4.55887
,-2.84974,0.57562\H,0,5.06934,-0.97992,-1.80025\H,0,6.15929,-1.24123,-
0.44173\H,0,0.65189,-0.51495,-1.10864\H,0,-1.43749,-0.88909,-1.13313\H
,0,-3.17952,-2.94591,-1.89527\H,0,-4.04607,-3.69158,-0.53271\H,0,-2.31
68,-4.01215,-0.7682\H,0,-2.68047,0.02087,1.73486\H,0,-4.28966,1.89357,
1.94179\H,0,-6.27152,2.00275,0.44009\H,0,-6.62859,0.21627,-1.25003\H,0
,-5.03493,-1.65108,-1.44109\H,0,-0.00999,2.85386,2.16975\H,0,0.94832,4
.29762,1.82097\H,0,2.05806,2.5891,3.37061\H,0,3.0364,3.04171,1.95968\H
,0,1.28681,0.53422,2.19687\H,0,3.04753,0.59132,2.19505\H,0,-1.24077,3.
21966,-0.19921\H,0,-0.27823,4.69771,-0.04301\H,0,0.91056,4.20874,-2.14
582\H,0,-0.83331,4.18625,-2.45722\H,0,0.48762,2.18018,-3.42186\H,0,-0.
91452,1.77688,-2.44836\H,0,-2.55642,-2.46363,1.06806\\Version=AM64L-G0
3RevD.02\State=1-A\HF=-1529.7549559\RMSD=4.106e-09\Thermal=0.\Dipole=-
0.0648415,3.0706615,-0.7545704\PG=C01 [X(C22H33N5S1)]\\@

3TABD-b

1\1\FAU-CCC-CCDH159\SP\RB3PW91\6-31G(d)\C22H33N5S1\SHUBINA\10-Aug-2008
\0\\#P B3PW91/6-31G(d) SCF=Tight Name=SHUBINA SCRF=(PCM,Solvent=THF)\\
Catalyst2\\0,1\C,0,4.11614,-0.7813,-0.39996\C,0,2.65994,-0.64929,-0.86
658\C,0,1.75119,-1.61578,-0.07974\C,0,2.23488,-3.04942,-0.33156\C,0,3.
68741,-3.21818,0.11971\C,0,4.61286,-2.21925,-0.57886\N,0,0.37725,-1.40
068,-0.52356\C,0,-0.7267,-1.75957,0.18889\S,0,-0.68622,-2.7018,1.58105
\N,0,-1.88588,-1.26877,-0.33922\C,0,-3.22802,-1.57769,0.14371\C,0,-4.1
0821,-0.34995,-0.04775\C,0,-3.75663,-2.86919,-0.47865\C,0,-3.69158,0.8
716,0.50087\C,0,-4.46681,2.01846,0.36757\C,0,-5.6798,1.96825,-0.32166\
C,0,-6.10456,0.76233,-0.87073\C,0,-5.32407,-0.38751,-0.73494\N,0,2.140
44,0.72832,-0.95344\C,0,2.02116,1.49533,0.21984\N,0,1.53747,2.79806,0.
05062\C,0,1.73299,3.53377,-1.18565\C,0,1.56452,2.62323,-2.38473\C,0,2.
46093,1.41261,-2.20311\C,0,1.51463,3.63895,1.23911\C,0,1.07325,2.82717
,2.4443\C,0,2.00748,1.63025,2.58969\N,0,2.25794,0.93107,1.34755\H,0,4.
17373,-0.48035,0.65057\H,0,4.74638,-0.08842,-0.97359\H,0,2.62317,-0.99
807,-1.9107\H,0,1.81977,-1.38888,0.98833\H,0,2.14142,-3.27769,-1.40411
\H,0,1.58057,-3.7411,0.20702\H,0,4.02176,-4.24556,-0.0718\H,0,3.7444,-
3.06901,1.20721\H,0,4.6592,-2.45982,-1.65216\H,0,5.6375,-2.31031,-0.19
616\H,0,0.28677,-0.54387,-1.0648\H,0,-1.83975,-0.87056,-1.2694\H,0,-3.
85774,-2.78453,-1.56749\H,0,-4.73229,-3.13939,-0.06005\H,0,-3.05603,-3
.67848,-0.25489\H,0,-2.74637,0.91255,1.03726\H,0,-4.1273,2.95391,0.805
98\H,0,-6.28823,2.86305,-0.42656\H,0,-7.04755,0.71023,-1.40961\H,0,-5.
6756,-1.31854,-1.1697\H,0,2.73352,4.0048,-1.21256\H,0,0.99952,4.34986,
-1.21172\H,0,1.82642,3.15812,-3.30447\H,0,0.51958,2.3001,-2.46336\H,0,
3.52002,1.72392,-2.24126\H,0,2.31047,0.70146,-3.02256\H,0,2.5072,4.087
73,1.42376\H,0,0.81982,4.46587,1.04502\H,0,0.04429,2.48174,2.28603\H,0
,1.07951,3.45033,3.34628\H,0,1.59341,0.91051,3.30754\H,0,2.97026,1.958
38,3.01338\H,0,-3.10476,-1.75405,1.21957\\Version=AM64L-G03RevD.02\Sta
te=1-A\HF=-1529.7446412\RMSD=8.195e-09\Thermal=0.\Dipole=0.2504432,2.9
391154,-2.6529998\PG=C01 [X(C22H33N5S1)]\\@

Init13

1\1\FAU-CCC-CCDH158\SP\RB3PW91\6-31G(d)\C37H52N6O6S1\SHUBINA\11-Aug-20
08\0\\#P B3PW91/6-31G(d) SCF=Tight Name=SHUBINA SCRF=(PCM,Solvent=THF)
\\Aza INIT 1R\\0,1\C,0,-1.52159,4.68467,-2.32805\C,0,-1.41721,3.31906,
-1.63554\C,0,-1.69766,3.4669,-0.11687\C,0,-0.7172,4.47895,0.4967\C,0,-
0.74763,5.82703,-0.22485\C,0,-0.50437,5.65742,-1.72487\N,0,-1.60805,2.
20228,0.58152\C,0,-2.64781,1.54307,1.15118\S,0,-4.27393,2.02768,0.9835
5\N,0,-2.27926,0.46171,1.87568\C,0,-3.19469,-0.46677,2.52069\C,0,-3.43
234,-1.72415,1.69481\C,0,-2.67811,-0.80722,3.92164\C,0,-4.73466,-2.141
99,1.40608\C,0,-4.96541,-3.31921,0.69353\C,0,-3.89282,-4.09277,0.25269
\C,0,-2.58702,-3.68075,0.52488\C,0,-2.36341,-2.50612,1.24161\N,0,-2.27
803,2.29511,-2.26601\C,0,-1.91895,0.98457,-2.24834\N,0,-2.81025,0.0232
2,-2.59796\C,0,-4.23461,0.32594,-2.68696\C,0,-4.40974,1.66881,-3.35991
\C,0,-3.65544,2.71088,-2.56063\C,0,-2.48131,-1.40306,-2.51728\C,0,-0.9
9975,-1.6514,-2.72687\C,0,-0.2273,-0.74768,-1.79006\N,0,-0.65953,0.632
65,-1.96972\H,0,-2.52278,5.11569,-2.19663\H,0,-1.35497,4.56283,-3.4062
3\H,0,-0.37838,3.0128,-1.76046\H,0,-2.72626,3.82083,0.01809\H,0,0.2938
4,4.05451,0.44518\H,0,-0.96648,4.60183,1.55716\H,0,0.00313,6.49843,0.2
1001\H,0,-1.7248,6.30759,-0.06898\H,0,0.51247,5.27672,-1.89234\H,0,-0.

57204,6.6245,-2.23857\H,0,-0.66868,1.81586,0.73263\H,0,-1.27922,0.2559
1,1.91614\H,0,-1.70474,-1.30974,3.87645\H,0,-3.37571,-1.48242,4.42772\
H,0,-2.57201,0.1037,4.52028\H,0,-5.57441,-1.53604,1.7394\H,0,-5.98607,
-3.62914,0.48064\H,0,-4.0713,-5.01092,-0.30208\H,0,-1.73568,-4.25813,0
.17429\H,0,-1.33983,-2.19673,1.4383\H,0,-4.68473,0.32548,-1.68422\H,0,
-4.69934,-0.47364,-3.27241\H,0,-5.46841,1.94493,-3.39885\H,0,-4.03444,
1.61826,-4.38936\H,0,-4.17331,2.9036,-1.6118\H,0,-3.60844,3.6403,-3.13
218\H,0,-2.80301,-1.79935,-1.5453\H,0,-3.07004,-1.90863,-3.29123\H,0,-
0.72397,-1.44308,-3.76777\H,0,-0.77072,-2.69961,-2.51262\H,0,0.84626,-
0.77332,-1.99367\H,0,-0.3668,-1.04889,-0.7465\H,0,-4.14502,0.06658,2.6
1244\H,0,0.00534,1.30777,-1.56742\C,0,2.7544,0.76915,0.49888\C,0,1.779
81,0.25142,1.36889\C,0,2.48928,1.68275,-0.53617\O,0,0.5484,0.4463,1.38
926\O,0,2.34453,-0.58884,2.31245\O,0,1.39262,2.15079,-0.91822\O,0,3.63
79,2.0809,-1.16977\C,0,1.47457,-1.09482,3.32291\C,0,2.33349,-1.72131,4
.40351\C,0,3.48628,3.00503,-2.2415\C,0,4.86096,3.2387,-2.83564\H,0,3.7
8889,0.50589,0.67651\H,0,0.79318,-1.83496,2.88535\H,0,0.86662,-0.27733
,3.72541\H,0,3.01048,-0.97957,4.83977\H,0,2.93634,-2.54372,4.00401\H,0
,1.69955,-2.12186,5.20239\H,0,3.05185,3.94103,-1.86792\H,0,2.79099,2.6
0227,-2.98829\H,0,5.28598,2.30219,-3.21144\H,0,5.54501,3.64562,-2.0838
6\H,0,4.79918,3.94925,-3.66757\C,0,7.72712,-3.16759,1.19574\C,0,6.8614
4,-2.21051,1.7214\C,0,5.49542,-2.29663,1.4722\C,0,4.97514,-3.34369,0.6
9103\C,0,5.86103,-4.30373,0.16764\C,0,7.22276,-4.21445,0.41868\C,0,3.5
3717,-3.37412,0.47207\C,0,2.8514,-4.28881,-0.23325\N,0,1.42319,-4.1842
4,-0.36292\O,0,0.87725,-5.01594,-1.08961\O,0,0.82376,-3.29293,0.23865\
H,0,8.79477,-3.10144,1.38848\H,0,7.24955,-1.39468,2.32495\H,0,4.81258,
-1.55545,1.88003\H,0,5.48297,-5.12221,-0.43871\H,0,7.89743,-4.96126,0.
00875\H,0,2.96269,-2.56816,0.92669\H,0,3.24241,-5.14358,-0.76761\\Vers
ion=AM64L-G03RevD.02\State=1-A\HF=-2618.4178756\RMSD=7.839e-09\Thermal
=0.\Dipole=1.2538443,-0.8539138,-2.4491173\PG=C01 [X(C37H52N6O6S1)]\\@

TS13

1\1\FAU-CCC-CCDH160\SP\RB3PW91\6-31G(d)\C37H52N6O6S1\SHUBINA\11-Aug-20
08\0\\#P B3PW91/6-31G(d) SCF=Tight Name=SHUBINA SCRF=(PCM,Solvent=THF)
\\Aza TS1 R\\0,1\C,0,-1.017792,4.300402,-1.222357\C,0,-1.185187,2.8401
64,-0.780976\C,0,-1.799051,2.724657,0.637084\C,0,-1.058566,3.616964,1.
645671\C,0,-0.891094,5.056773,1.161899\C,0,-0.199509,5.090996,-0.20060
2\N,0,-1.773427,1.342283,1.071635\C,0,-2.855338,0.570687,1.351106\S,0,
-4.455318,1.143641,1.196476\N,0,-2.557821,-0.678838,1.760701\C,0,-3.53
1692,-1.681494,2.182606\C,0,-3.920098,-2.62949,1.053537\C,0,-2.977573,
-2.417576,3.405122\C,0,-5.215746,-2.589881,0.528514\C,0,-5.606638,-3.4
68461,-0.482317\C,0,-4.702549,-4.402595,-0.9843\C,0,-3.404688,-4.44599
8,-0.472213\C,0,-3.014762,-3.566027,0.536897\N,0,-1.981978,2.058702,-1
.758086\C,0,-1.700087,0.755336,-2.024692\N,0,-2.646761,-0.050843,-2.56
1564\C,0,-4.041611,0.377637,-2.607541\C,0,-4.073348,1.810783,-3.089252
\C,0,-3.280631,2.653684,-2.111279\C,0,-2.395893,-1.460167,-2.868829\C,
0,-0.930271,-1.704641,-3.168724\C,0,-0.111423,-1.130545,-2.03134\N,0,-
0.474534,0.266161,-1.823298\H,0,-1.994754,4.791442,-1.311723\H,0,-0.54
9736,4.3246,-2.214646\H,0,-0.179938,2.416151,-0.727944\H,0,-2.852758,3
.019689,0.606763\H,0,-0.070718,3.179014,1.823932\H,0,-1.607642,3.58223
3,2.594143\H,0,-0.315286,5.628979,1.899809\H,0,-1.872875,5.54715,1.083
78\H,0,0.809195,4.663434,-0.115515\H,0,-0.078605,6.123646,-0.55042\H,0
,-0.840875,0.982593,1.273185\H,0,-1.589174,-1.012547,1.705193\H,0,-2.0
07297,-2.876689,3.188354\H,0,-3.670302,-3.203818,3.722253\H,0,-2.83939
4,-1.715153,4.233658\H,0,-5.918947,-1.856185,0.91486\H,0,-6.621076,-3.
42564,-0.872401\H,0,-5.006993,-5.096417,-1.764594\H,0,-2.693542,-5.176
095,-0.852878\H,0,-1.998168,-3.60399,0.91958\H,0,-4.506131,0.281623,-1
.616665\H,0,-4.56242,-0.289403,-3.300125\H,0,-5.099963,2.188804,-3.129
65\H,0,-3.650116,1.870972,-4.099303\H,0,-3.867638,2.782342,-1.193671\H
,0,-3.092694,3.63787,-2.547693\H,0,-2.729052,-2.082196,-2.02751\H,0,-3
.016507,-1.715075,-3.735264\H,0,-0.65213,-1.227212,-4.116192\H,0,-0.75
1667,-2.780215,-3.265597\H,0,0.95624,-1.128013,-2.257386\H,0,-0.266877
,-1.692704,-1.101971\H,0,-4.428258,-1.126184,2.471202\H,0,0.296885,0.8
36839,-1.479149\C,0,3.422365,0.799521,0.569681\C,0,2.329408,1.194112,1
.424548\C,0,3.290944,0.899146,-0.864401\O,0,1.143761,1.27986,1.101739\
O,0,2.762577,1.41627,2.687759\O,0,2.261421,0.868301,-1.541404\O,0,4.50
8927,0.996236,-1.456917\C,0,1.762225,1.608688,3.696645\C,0,2.450466,1.
506811,5.041166\C,0,4.515503,1.039595,-2.886341\C,0,5.958247,1.163816,

-3.328468\H,0,4.417726,0.978598,0.959403\H,0,0.991201,0.842319,3.57834
\H,0,1.300413,2.593766,3.560254\H,0,3.248394,2.251124,5.134936\H,0,2.8
85163,0.511336,5.174042\H,0,1.725459,1.675043,5.845018\H,0,3.909713,1.
887262,-3.226992\H,0,4.050534,0.128301,-3.279917\H,0,6.544532,0.310698
,-2.973663\H,0,6.409613,2.079402,-2.932859\H,0,6.015431,1.195863,-4.42
2223\C,0,6.956844,-2.688342,-0.895659\C,0,6.986414,-2.139486,0.385615\
C,0,5.809027,-1.70456,0.98522\C,0,4.576215,-1.81012,0.323834\C,0,4.562
894,-2.360196,-0.966847\C,0,5.739508,-2.795361,-1.568686\C,0,3.352832,
-1.375023,1.026317\C,0,2.103233,-1.899219,0.710283\N,0,1.039361,-1.784
224,1.576526\O,0,-0.098991,-2.157292,1.155448\O,0,1.185239,-1.355696,2
.734998\H,0,7.874363,-3.032829,-1.365823\H,0,7.92837,-2.053326,0.92130
9\H,0,5.836892,-1.280012,1.986361\H,0,3.625339,-2.449185,-1.508136\H,0
,5.705872,-3.224624,-2.567148\H,0,3.488663,-1.108606,2.070769\H,0,1.84
3789,-2.350876,-0.233614\\Version=AM64L-G03RevD.02\State=1-A\HF=-2618.
3975699\RMSD=5.562e-09\Thermal=0.\Dipole=0.5743984,0.5179885,-4.208538
6\PG=C01 [X(C37H52N6O6S1)]\\@

Fin13

1\1\FAU-CCC-CCDH160\SP\RB3PW91\6-31G(d)\C37H52N6O6S1\SHUBINA\11-Aug-20
08\0\\#P B3PW91/6-31G(d) SCF=Tight Name=SHUBINA SCRF=(PCM,Solvent=THF)
\\Aza Fin 1R\\0,1\C,0,-0.54226,4.11248,-1.32218\C,0,-0.90398,2.70364,-
0.83605\C,0,-1.40798,2.70245,0.62987\C,0,-0.42282,3.44228,1.5477\C,0,-
0.06448,4.83597,1.03201\C,0,0.48597,4.75977,-0.3921\N,0,-1.60021,1.344
33,1.0821\C,0,-2.7797,0.79166,1.46199\S,0,-4.26921,1.62951,1.40602\N,0
,-2.65503,-0.47964,1.89012\C,0,-3.74066,-1.32144,2.36352\C,0,-4.18789,
-2.33366,1.31583\C,0,-3.31986,-2.00747,3.66739\C,0,-5.51324,-2.34411,0
.87101\C,0,-5.95138,-3.29032,-0.05629\C,0,-5.06481,-4.2434,-0.55323\C,
0,-3.73676,-4.2365,-0.12245\C,0,-3.29955,-3.28884,0.80204\N,0,-1.88472
,2.04267,-1.73191\C,0,-1.80561,0.71825,-2.02299\N,0,-2.89759,0.03895,-
2.4418\C,0,-4.22001,0.65185,-2.35665\C,0,-4.10713,2.07711,-2.85214\C,0
,-3.1202,2.80848,-1.96445\C,0,-2.86791,-1.40319,-2.69586\C,0,-1.49175,
-1.85992,-3.14241\C,0,-0.46929,-1.35939,-2.14279\N,0,-0.63007,0.08211,
-1.9788\H,0,-1.43267,4.75399,-1.33817\H,0,-0.16812,4.05399,-2.35272\H,
0,0.02472,2.12794,-0.84575\H,0,-2.38361,3.19688,0.68169\H,0,0.49651,2.
85176,1.63387\H,0,-0.87291,3.49692,2.54618\H,0,0.67383,5.29621,1.69972
\H,0,-0.95259,5.48574,1.04846\H,0,1.41354,4.17309,-0.38915\H,0,0.7356,
5.76062,-0.7665\H,0,-0.73813,0.80048,1.26084\H,0,-1.74027,-0.93458,1.7
245\H,0,-2.43204,-2.63249,3.51777\H,0,-4.12615,-2.64772,4.04112\H,0,-3
.08438,-1.2556,4.42774\H,0,-6.2068,-1.59934,1.25494\H,0,-6.98779,-3.28
36,-0.38635\H,0,-5.40491,-4.9886,-1.26872\H,0,-3.03749,-4.97812,-0.502
89\H,0,-2.25718,-3.28384,1.11332\H,0,-4.5927,0.62276,-1.32336\H,0,-4.8
9123,0.06113,-2.98675\H,0,-5.07448,2.58655,-2.79819\H,0,-3.77977,2.079
8,-3.89911\H,0,-3.5943,3.01004,-0.99627\H,0,-2.84382,3.75965,-2.42749\
H,0,-3.17768,-1.93832,-1.78898\H,0,-3.61333,-1.6071,-3.4725\H,0,-1.271
07,-1.46872,-4.14331\H,0,-1.47119,-2.95306,-3.19411\H,0,0.5517,-1.5168
6,-2.49706\H,0,-0.57343,-1.84384,-1.16221\H,0,-4.57868,-0.64805,2.5648
8\H,0,0.20423,0.55989,-1.66062\C,0,3.64225,0.10784,0.45645\C,0,3.14004
,1.19623,1.39628\C,0,3.28017,0.48806,-0.96388\O,0,2.71576,2.27273,1.02
9\O,0,3.41686,0.88613,2.66735\O,0,2.18104,0.41045,-1.47716\O,0,4.35603
,0.93717,-1.62256\C,0,2.95312,1.81662,3.66102\C,0,3.42929,1.31837,5.00
771\C,0,4.13941,1.37665,-2.97594\C,0,5.46637,1.86605,-3.51242\H,0,4.73
549,0.17169,0.52566\H,0,1.86123,1.85367,3.60464\H,0,3.34428,2.81202,3.
42664\H,0,4.52258,1.27101,5.04615\H,0,3.03069,0.32051,5.21525\H,0,3.08
665,1.99655,5.79666\H,0,3.38059,2.16608,-2.96997\H,0,3.74354,0.53846,-
3.55928\H,0,6.21181,1.06486,-3.50097\H,0,5.84561,2.69945,-2.91305\H,0,
5.34565,2.21073,-4.54502\C,0,6.18047,-4.01562,-0.95165\C,0,6.33863,-3.
60473,0.37052\C,0,5.3974,-2.76113,0.95816\C,0,4.28405,-2.31328,0.24027
\C,0,4.1358,-2.73464,-1.08658\C,0,5.07443,-3.57748,-1.67842\C,0,3.2880
9,-1.36019,0.88519\C,0,1.89394,-1.85501,0.65987\N,0,0.81674,-1.22034,1
.07401\O,0,-0.34705,-1.77157,0.95424\O,0,0.88661,-0.0367,1.57546\H,0,6
.91139,-4.67619,-1.41126\H,0,7.19352,-3.94569,0.94946\H,0,5.5247,-2.44
838,1.99283\H,0,3.26951,-2.40301,-1.65443\H,0,4.93973,-3.89582,-2.7096
\H,0,3.49741,-1.34956,1.96474\H,0,1.72023,-2.86095,0.30538\\Version=AM
64L-G03RevD.02\State=1-A\HF=-2618.4246743\RMSD=4.231e-09\Thermal=0.\Di
pole=1.3946914,-0.0084721,-4.0059284\PG=C01 [X(C37H52N6O6S1)]\\@

Init14

1\1\FAU-CCC-CCDH161\SP\RB3PW91\6-31G(d)\C37H52N6O6S1\SHUBINA\11-Aug-20
08\0\\#P B3PW91/6-31G(d) SCF=Tight Name=SHUBINA SCRF=(PCM,Solvent=THF)
\\Aza INIT 1S\\0,1\C,0,-1.34556,4.59393,-0.71585\C,0,-1.3563,3.07463,-
0.51048\C,0,-1.41439,2.71099,0.99516\C,0,-0.25724,3.38728,1.74534\C,0,
-0.20604,4.89579,1.50517\C,0,-0.15199,5.21827,0.01127\N,0,-1.37303,1.2
7769,1.18694\C,0,-2.36803,0.5447,1.76094\S,0,-3.9087,1.1803,2.13123\N,
0,-2.02307,-0.73329,1.9996\C,0,-2.87066,-1.71849,2.65559\C,0,-3.67772,
-2.55805,1.67371\C,0,-1.99828,-2.59435,3.55994\C,0,-5.06924,-2.62047,1
.78318\C,0,-5.81886,-3.43086,0.93007\C,0,-5.18256,-4.1907,-0.04915\C,0
,-3.79343,-4.12675,-0.17298\C,0,-3.04473,-3.31474,0.67854\N,0,-2.44141
,2.41263,-1.26876\C,0,-2.28106,1.15338,-1.75201\N,0,-3.35106,0.3782,-2
.03119\C,0,-4.68303,0.77458,-1.58072\C,0,-4.85163,2.25379,-1.85355\C,0
,-3.77528,3.00834,-1.09799\C,0,-3.21438,-1.00307,-2.49968\C,0,-1.90952
,-1.21948,-3.24374\C,0,-0.76973,-0.69042,-2.39525\N,0,-1.05755,0.6923,
-2.03484\H,0,-2.26252,5.0432,-0.31352\H,0,-1.31571,4.81842,-1.78982\H,
0,-0.39878,2.71866,-0.89727\H,0,-2.36764,3.06154,1.40781\H,0,0.6864,2.
92383,1.42953\H,0,-0.36533,3.16415,2.81251\H,0,0.66238,5.32547,2.01985
\H,0,-1.09532,5.37078,1.9448\H,0,0.78429,4.83282,-0.4173\H,0,-0.14997,
6.30315,-0.15133\H,0,-0.43183,0.86661,1.26711\H,0,-1.10861,-1.0621,1.6
627\H,0,-1.22979,-3.11536,2.97887\H,0,-2.61299,-3.34468,4.06783\H,0,-1
.49723,-1.97789,4.31363\H,0,-5.569,-2.02037,2.54007\H,0,-6.90115,-3.46
7,1.03195\H,0,-5.76312,-4.82969,-0.71066\H,0,-3.28868,-4.72084,-0.9324
4\H,0,-1.96332,-3.25449,0.56903\H,0,-4.80593,0.55129,-0.51205\H,0,-5.4
0549,0.18049,-2.14791\H,0,-5.83203,2.60175,-1.51305\H,0,-4.78186,2.440
8,-2.93218\H,0,-4.02004,3.01224,-0.02844\H,0,-3.73078,4.04083,-1.45593
\H,0,-3.28814,-1.68178,-1.64014\H,0,-4.06892,-1.20613,-3.15499\H,0,-1.
93088,-0.69693,-4.20749\H,0,-1.77787,-2.28801,-3.44096\H,0,-0.61826,-1
.2906,-1.48844\H,0,0.1654,-0.67797,-2.96048\H,0,-3.57691,-1.15447,3.27
196\H,0,-0.24001,1.26562,-1.85762\C,0,2.37387,-1.48641,0.99072\C,0,1.3
2585,-2.30976,0.53423\C,0,2.17819,-0.27671,1.68251\O,0,0.09176,-2.1312
8,0.58087\O,0,1.81772,-3.46102,-0.04256\O,0,1.13339,0.3186,1.98259\O,0
,3.3963,0.2826,2.06775\C,0,0.86171,-4.4193,-0.47103\C,0,1.62562,-5.602
35,-1.03345\C,0,3.31669,1.45561,2.874\C,0,4.68146,1.69476,3.48999\H,0,
3.38307,-1.86469,0.88901\H,0,0.19833,-3.98224,-1.22945\H,0,0.22822,-4.
72276,0.37211\H,0,2.27555,-6.04139,-0.26966\H,0,2.25262,-5.29477,-1.87
7\H,0,0.93047,-6.37484,-1.38153\H,0,2.54843,1.32093,3.64191\H,0,3.0095
1,2.31155,2.25827\H,0,5.44594,1.84546,2.71994\H,0,4.98117,0.84261,4.10
902\H,0,4.65729,2.58854,4.12384\C,0,8.40934,-0.5059,-0.75242\C,0,7.491
83,-0.56292,0.29501\C,0,6.17362,-0.16849,0.08944\C,0,5.75348,0.28336,-
1.17487\C,0,6.69161,0.33857,-2.22335\C,0,8.00583,-0.05186,-2.01161\C,0
,4.36039,0.66957,-1.32701\C,0,3.75572,1.00663,-2.48193\N,0,2.36617,1.3
3071,-2.51403\O,0,1.8445,1.43674,-3.62559\O,0,1.74431,1.47587,-1.45207
\H,0,9.43983,-0.81219,-0.59185\H,0,7.80214,-0.91537,1.27482\H,0,5.4497
3,-0.20841,0.90005\H,0,6.39429,0.69532,-3.20569\H,0,8.72208,-0.0029,-2
.82739\H,0,3.75167,0.65887,-0.42519\H,0,4.18781,1.00826,-3.47284\\Vers
ion=AM64L-G03RevD.02\State=1-A\HF=-2618.4102567\RMSD=5.750e-09\Thermal
=0.\Dipole=0.9866721,1.1740434,-3.8842565\PG=C01 [X(C37H52N6O6S1)]\\@

TS14

1\1\FAU-CCC-CCDH162\SP\RB3PW91\6-31G(d)\C37H52N6O6S1\SHUBINA\11-Aug-20
08\0\\#P B3PW91/6-31G(d) SCF=Tight Name=SHUBINA SCRF=(PCM,Solvent=THF)
\\Aza_TS1 S\\0,1\C,0,-1.371704,4.591875,-0.998584\C,0,-1.43054,3.07861
2,-0.745781\C,0,-1.005687,2.740367,0.707883\C,0,0.371618,3.350498,1.01
4923\C,0,0.443744,4.845513,0.70954\C,0,0.037596,5.126765,-0.736774\N,0
,-0.950436,1.309704,0.932942\C,0,-1.805588,0.588023,1.704386\S,0,-3.23
6457,1.228239,2.373182\N,0,-1.416749,-0.690099,1.88023\C,0,-2.129527,-
1.690874,2.657626\C,0,-2.996256,-2.593368,1.788554\C,0,-1.12142,-2.497
409,3.481595\C,0,-4.36264,-2.72271,2.052075\C,0,-5.160216,-3.5798,1.29
2638\C,0,-4.59886,-4.319366,0.253587\C,0,-3.236418,-4.191091,-0.022958
\C,0,-2.441337,-3.334537,0.736865\N,0,-2.758117,2.511475,-1.077025\C,0
,-2.883958,1.22977,-1.514578\N,0,-4.097945,0.622426,-1.527209\C,0,-5.2
37629,1.207072,-0.830275\C,0,-5.229273,2.701771,-1.053163\C,0,-3.91115
,3.257129,-0.555516\C,0,-4.232208,-0.795711,-1.866084\C,0,-3.219905,-1
.203006,-2.91851\C,0,-1.841617,-0.81933,-2.425612\N,0,-1.832243,0.5830
82,-2.024385\H,0,-2.057668,5.127011,-0.329634\H,0,-1.689563,4.79958,-2
.028294\H,0,-0.693464,2.640899,-1.422104\H,0,-1.755185,3.147508,1.3971

02\H,0,1.122617,2.822834,0.411993\H,0,0.61169,3.146258,2.063986\H,0,1.
459079,5.213906,0.902016\H,0,-0.222788,5.398467,1.387733\H,0,0.751889,
4.650233,-1.422593\H,0,0.065638,6.203236,-0.945946\H,0,-0.035786,0.880
215,0.784085\H,0,-0.541428,-0.982659,1.437301\H,0,-0.405546,-3.016637,
2.834258\H,0,-1.639353,-3.249929,4.085037\H,0,-0.561228,-1.832225,4.14
6492\H,0,-4.805301,-2.139701,2.856598\H,0,-6.221173,-3.668696,1.514797
\H,0,-5.216554,-4.992943,-0.335774\H,0,-2.789102,-4.767794,-0.82978\H,
0,-1.383156,-3.230568,0.506142\H,0,-5.185483,0.966908,0.240698\H,0,-6.
141009,0.751168,-1.248094\H,0,-6.047337,3.178055,-0.503154\H,0,-5.3668
82,2.919224,-2.119434\H,0,-3.884014,3.223547,0.541287\H,0,-3.814392,4.
294436,-0.881949\H,0,-4.115662,-1.405604,-0.960041\H,0,-5.253954,-0.93
9048,-2.23386\H,0,-3.439039,-0.700194,-3.868061\H,0,-3.277687,-2.28317
4,-3.084534\H,0,-1.538498,-1.443534,-1.57617\H,0,-1.080243,-0.917043,-
3.202726\H,0,-2.788611,-1.139447,3.33434\H,0,-0.883368,0.962173,-1.935
319\C,0,2.912689,-1.256316,0.591447\C,0,1.86166,-1.985332,-0.067083\C,
0,2.62034,-0.379641,1.697906\O,0,0.64118,-1.857448,0.051643\O,0,2.3879
36,-2.880821,-0.946071\O,0,1.569638,0.21475,1.932244\O,0,3.717176,-0.2
01426,2.485248\C,0,1.454747,-3.638016,-1.721554\C,0,2.249803,-4.598105
,-2.581717\C,0,3.554495,0.696648,3.587563\C,0,4.860955,0.716627,4.3529
55\H,0,3.882025,-1.737657,0.602667\H,0,0.855177,-2.953674,-2.33197\H,0
,0.772928,-4.172845,-1.050443\H,0,2.855144,-5.267556,-1.962084\H,0,2.9
18568,-4.055657,-3.257729\H,0,1.571759,-5.208273,-3.188503\H,0,2.72145
,0.356885,4.212959\H,0,3.288755,1.692132,3.212277\H,0,5.683464,1.04938
1,3.711696\H,0,5.106085,-0.281188,4.730642\H,0,4.786975,1.400261,5.205
866\C,0,7.744574,0.017152,-0.667312\C,0,7.02996,1.013712,-0.005736\C,0
,5.643401,1.070343,-0.1172\C,0,4.94614,0.141988,-0.903334\C,0,5.677305
,-0.862824,-1.557416\C,0,7.060913,-0.924025,-1.439086\C,0,3.485449,0.2
67733,-1.040221\C,0,2.860241,-0.034699,-2.242381\N,0,1.570629,0.365311
,-2.502394\O,0,1.040044,0.029888,-3.580533\O,0,0.948298,1.077487,-1.65
7398\H,0,8.826817,-0.031292,-0.578383\H,0,7.552796,1.749229,0.600509\H
,0,5.089483,1.849149,0.400681\H,0,5.151551,-1.616533,-2.136925\H,0,7.6
10002,-1.712267,-1.948097\H,0,3.005612,1.03686,-0.444718\H,0,3.288972,
-0.630614,-3.033947\\Version=AM64L-G03RevD.02\State=1-A\HF=-2618.40117
73\RMSD=7.874e-09\Thermal=0.\Dipole=-1.153963,0.4305293,-1.4879182\PG=
C01 [X(C37H52N6O6S1)]\\@

Fin14

1\1\FAU-CCC-CCDH154\SP\RB3PW91\6-31G(d)\C37H52N6O6S1\SHUBINA\11-Aug-20
08\0\\#P B3PW91/6-31G(d) SCF=Tight Name=SHUBINA SCRF=(PCM,Solvent=THF)
\\Aza Fin 1S\\0,1\C,0,2.25971,4.48255,-0.12184\C,0,2.00164,2.968,-0.08
648\C,0,1.2716,2.47225,-1.3607\C,0,-0.00015,3.30109,-1.60205\C,0,0.268
03,4.80592,-1.61947\C,0,0.95304,5.25155,-0.32761\N,0,0.9319,1.06805,-1
.23826\C,0,1.35352,0.06049,-2.03894\S,0,2.50883,0.26159,-3.27224\N,0,0
.76526,-1.1303,-1.77909\C,0,1.15133,-2.39721,-2.38321\C,0,2.21506,-3.1
193,-1.56483\C,0,-0.09346,-3.26181,-2.59404\C,0,3.42345,-3.49793,-2.15
486\C,0,4.39376,-4.18299,-1.42295\C,0,4.16781,-4.49645,-0.08439\C,0,2.
96603,-4.11667,0.51669\C,0,1.99855,-3.43268,-0.2171\N,0,3.24353,2.2118
5,0.1623\C,0,3.32726,1.24029,1.10549\N,0,4.51814,0.60981,1.32169\C,0,5
.67434,0.77247,0.44716\C,0,5.66169,2.13369,-0.21322\C,0,4.30231,2.3359
9,-0.84707\C,0,4.59633,-0.52509,2.24041\C,0,3.68402,-0.30778,3.43424\C
,0,2.28157,-0.07943,2.91469\N,0,2.28325,0.94231,1.87577\H,0,2.94288,4.
75031,-0.9389\H,0,2.747,4.77954,0.81504\H,0,1.32607,2.79782,0.75392\H,
0,1.93742,2.55717,-2.22718\H,0,-0.71515,3.067,-0.80236\H,0,-0.44765,2.
97168,-2.54711\H,0,-0.67492,5.34858,-1.75963\H,0,0.90703,5.06081,-2.47
809\H,0,0.28191,5.0745,0.52408\H,0,1.1606,6.32862,-0.34956\H,0,0.27579
,0.85658,-0.4806\H,0,0.02561,-1.16861,-1.08001\H,0,-0.57429,-3.50572,-
1.64052\H,0,0.18156,-4.20084,-3.08524\H,0,-0.82063,-2.73133,-3.21676\H
,0,3.60866,-3.24188,-3.1954\H,0,5.3281,-4.46936,-1.90025\H,0,4.91983,-
5.03574,0.48696\H,0,2.77834,-4.36476,1.55952\H,0,1.06699,-3.1343,0.259
01\H,0,5.67822,-0.02217,-0.31362\H,0,6.57185,0.64417,1.06343\H,0,6.443
83,2.18184,-0.97778\H,0,5.85941,2.91824,0.52707\H,0,4.12465,1.61491,-1
.65736\H,0,4.23274,3.33455,-1.27881\H,0,4.33831,-1.45694,1.71579\H,0,5
.64154,-0.60491,2.55782\H,0,4.02638,0.55954,4.01118\H,0,3.71058,-1.182
14,4.09265\H,0,1.87835,-1.01211,2.49486\H,0,1.58092,0.26297,3.68033\H,
0,1.58429,-2.14755,-3.35622\H,0,1.30962,1.25722,1.64231\C,0,-3.36773,-
0.91052,0.60794\C,0,-2.23,-1.81,1.06114\C,0,-3.70129,-1.19756,-0.84931
\O,0,-1.13454,-1.87665,0.53707\O,0,-2.58322,-2.52754,2.13173\O,0,-3.00

566,-0.88258,-1.78845\O,0,-4.87023,-1.84636,-0.96079\C,0,-1.55995,-3.3
5945,2.71357\C,0,-2.15667,-4.0243,3.93401\C,0,-5.29272,-2.14181,-2.307
56\C,0,-6.63996,-2.82376,-2.22266\H,0,-4.24178,-1.12981,1.22467\H,0,-0
.70383,-2.72652,2.9673\H,0,-1.23257,-4.08931,1.96551\H,0,-3.02018,-4.6
3973,3.66274\H,0,-2.47882,-3.27708,4.6656\H,0,-1.40912,-4.67006,4.4070
2\H,0,-4.53681,-2.77701,-2.78139\H,0,-5.33828,-1.20481,-2.87166\H,0,-7
.37694,-2.17352,-1.74111\H,0,-6.57385,-3.75451,-1.65024\H,0,-6.99787,-
3.0639,-3.22956\C,0,-6.29259,3.17722,-0.0923\C,0,-5.27413,2.99827,-1.0
255\C,0,-4.19551,2.16355,-0.7342\C,0,-4.11249,1.49864,0.49413\C,0,-5.1
4395,1.68552,1.42267\C,0,-6.22372,2.51592,1.13397\C,0,-2.93291,0.58147
,0.78811\C,0,-2.31122,0.79196,2.13045\N,0,-1.04882,1.16045,2.27981\O,0
,-0.51901,1.30274,3.41683\O,0,-0.30351,1.39456,1.21982\H,0,-7.13358,3.
82891,-0.31607\H,0,-5.31529,3.51027,-1.98405\H,0,-3.40746,2.01977,-1.4
6918\H,0,-5.10021,1.18592,2.38788\H,0,-7.01199,2.65096,1.87084\H,0,-2.
15898,0.76484,0.0405\H,0,-2.84252,0.64296,3.06071\\Version=AM64L-G03Re
vD.02\State=1-A\HF=-2618.4309272\RMSD=4.123e-09\Thermal=0.\Dipole=1.49
14125,-1.1423334,1.5656164\PG=C01 [X(C37H52N6O6S1)]\\@

Init15

1\1\FAU-CCC-CCDH158\SP\RB3PW91\6-31G(d)\C37H52N6O6S1\SHUBINA\11-Aug-20
08\0\\#P B3PW91/6-31G(d) SCF=Tight Name=SHUBINA SCRF=(PCM,Solvent=THF)
\\Aza INIT 2R\\0,1\C,0,-1.40786,4.6967,-0.64071\C,0,-1.42909,3.17113,-
0.48086\C,0,-1.09332,2.74565,0.97104\C,0,0.23797,3.37379,1.41355\C,0,0
.27399,4.88814,1.21177\C,0,-0.04202,5.25654,-0.23774\N,0,-1.02058,1.30
297,1.08098\C,0,-1.867,0.53155,1.8151\S,0,-3.26732,1.13738,2.58053\N,0
,-1.49582,-0.76184,1.87815\C,0,-2.20143,-1.79807,2.61623\C,0,-3.17862,
-2.57947,1.7471\C,0,-1.17754,-2.72436,3.27927\C,0,-4.51839,-2.69666,2.
12623\C,0,-5.41209,-3.45727,1.37154\C,0,-4.97461,-4.11171,0.22196\C,0,
-3.63996,-3.99247,-0.17049\C,0,-2.74834,-3.22983,0.5832\N,0,-2.70652,2
.58086,-0.94149\C,0,-2.74422,1.36466,-1.54295\N,0,-3.89519,0.65605,-1.
58449\C,0,-5.0358,1.05636,-0.76446\C,0,-5.19398,2.55687,-0.87693\C,0,-
3.91611,3.21092,-0.39024\C,0,-3.98708,-0.67135,-2.19695\C,0,-2.92437,-
0.87705,-3.25899\C,0,-1.57917,-0.48412,-2.68363\N,0,-1.66573,0.88023,-
2.17272\H,0,-2.16651,5.16355,0.00051\H,0,-1.65198,4.95758,-1.67851\H,0
,-0.62616,2.79376,-1.11453\H,0,-1.89489,3.08076,1.63925\H,0,1.05364,2.
90324,0.85036\H,0,0.40416,3.11339,2.46463\H,0,1.25883,5.2767,1.49986\H
,0,-0.45773,5.37102,1.87626\H,0,0.73389,4.84965,-0.90104\H,0,-0.03759,
6.34536,-0.37199\H,0,-0.09311,0.89323,0.90774\H,0,-0.69339,-1.06301,1.
30942\H,0,-0.53905,-3.20584,2.53066\H,0,-1.68803,-3.50911,3.84711\H,0,
-0.53684,-2.15478,3.96052\H,0,-4.86429,-2.18029,3.0188\H,0,-6.45083,-3
.53766,1.68382\H,0,-5.66656,-4.71233,-0.36397\H,0,-3.2894,-4.50402,-1.
06476\H,0,-1.71245,-3.12633,0.26526\H,0,-4.87941,0.7505,0.27894\H,0,-5
.91341,0.53321,-1.1551\H,0,-6.0276,2.90787,-0.26029\H,0,-5.40591,2.828
69,-1.91832\H,0,-3.87,3.14529,0.70376\H,0,-3.90965,4.26424,-0.68324\H,
0,-3.90024,-1.43429,-1.41252\H,0,-4.98973,-0.75641,-2.63178\H,0,-3.145
37,-0.26655,-4.14294\H,0,-2.91903,-1.92723,-3.56769\H,0,-1.25908,-1.16
17,-1.88216\H,0,-0.79402,-0.4836,-3.4398\H,0,-2.77754,-1.28328,3.39053
\H,0,-0.79213,1.40131,-2.1818\C,0,2.64681,-1.61928,0.54881\C,0,1.54179
,-2.23954,-0.07932\C,0,2.57155,-0.38949,1.24448\O,0,0.33771,-1.92404,-
0.05849\O,0,1.9421,-3.35657,-0.77794\O,0,1.65786,0.44938,1.25524\O,0,3
.72395,-0.1413,1.94891\C,0,0.91832,-4.12114,-1.40148\C,0,1.58783,-5.27
302,-2.12498\C,0,3.75494,1.07252,2.692\C,0,5.04024,1.07918,3.49536\H,0
,3.57479,-2.17345,0.60076\H,0,0.35265,-3.48995,-2.09757\H,0,0.21205,-4
.48841,-0.64576\H,0,2.14835,-5.89931,-1.42331\H,0,2.28462,-4.90259,-2.
8843\H,0,0.83724,-5.89823,-2.62165\H,0,2.87406,1.13336,3.34153\H,0,3.7
0832,1.93161,2.00987\H,0,5.91102,1.01254,2.83507\H,0,5.06979,0.22868,4
.18419\H,0,5.11726,2.00177,4.08202\C,0,7.97088,-0.33659,-0.37052\C,0,7
.26753,-1.46898,-0.77992\C,0,5.94596,-1.3532,-1.19344\C,0,5.29922,-0.1
0531,-1.20332\C,0,6.02194,1.02725,-0.78814\C,0,7.34343,0.91103,-0.3768
\C,0,3.92866,-0.04405,-1.68561\C,0,3.15842,1.06522,-1.76727\N,0,1.8640
6,0.97635,-2.32741\O,0,1.49483,-0.06398,-2.87202\O,0,1.14231,1.99399,-
2.25116\H,0,9.00503,-0.42368,-0.04762\H,0,7.74936,-2.44292,-0.77517\H,
0,5.39143,-2.23531,-1.50358\H,0,5.55088,2.00593,-0.7949\H,0,7.88994,1.
79632,-0.06242\H,0,3.50498,-0.96189,-2.08057\H,0,3.38081,2.05004,-1.38
421\\Version=AM64L-G03RevD.02\State=1-A\HF=-2618.4050044\RMSD=3.042e-0
9\Thermal=0.\Dipole=-0.4722647,1.2991822,-2.968396\PG=C01 [X(C37H52N6O
6S1)]\\@

TS15

1\1\FAU-CCC-CCDH162\SP\RB3PW91\6-31G(d)\C37H52N6O6S1\SHUBINA\11-Aug-20
08\0\\#P B3PW91/6-31G(d) SCF=Tight Name=SHUBINA SCRF=(PCM,Solvent=THF)
\\Aza_TS2 R\\0,1\C,0,-1.230097,4.56121,-0.90321\C,0,-1.343768,3.056797
,-0.619384\C,0,-0.983753,2.718147,0.849214\C,0,0.38267,3.320296,1.2174
46\C,0,0.489542,4.809715,0.897961\C,0,0.173917,5.070423,-0.574103\N,0,
-0.937646,1.28182,1.053593\C,0,-1.786312,0.561623,1.832623\S,0,-3.1945
88,1.209846,2.537294\N,0,-1.405833,-0.722765,1.998239\C,0,-2.162366,-1
.732509,2.722059\C,0,-3.117876,-2.505708,1.821385\C,0,-1.186114,-2.667
592,3.440891\C,0,-4.472612,-2.598818,2.150518\C,0,-5.350651,-3.345187,
1.364296\C,0,-4.882085,-4.009033,0.232262\C,0,-3.531744,-3.914275,-0.1
10104\C,0,-2.656107,-3.16672,0.676147\N,0,-2.67457,2.526926,-0.996406\
C,0,-2.816625,1.31727,-1.597767\N,0,-4.029227,0.710794,-1.619191\C,0,-
5.10757,1.193523,-0.762572\C,0,-5.158202,2.701161,-0.878185\C,0,-3.819
716,3.25876,-0.435135\C,0,-4.268809,-0.568839,-2.287774\C,0,-3.231228,
-0.847805,-3.35905\C,0,-1.859765,-0.619329,-2.757842\N,0,-1.792588,0.7
35133,-2.226655\H,0,-1.943252,5.130112,-0.292398\H,0,-1.481821,4.74565
5,-1.955209\H,0,-0.592187,2.572981,-1.244409\H,0,-1.753832,3.115366,1.
51995\H,0,1.16167,2.777133,0.666436\H,0,0.566412,3.127481,2.28031\H,0,
1.496069,5.169229,1.146423\H,0,-0.20943,5.380184,1.527496\H,0,0.909819
,4.559565,-1.209884\H,0,0.239812,6.141405,-0.802776\H,0,-0.025076,0.85
1988,0.887337\H,0,-0.573522,-1.043896,1.498042\H,0,-0.528459,-3.175916
,2.726784\H,0,-1.735738,-3.432459,3.998804\H,0,-0.562672,-2.10038,4.14
0204\H,0,-4.842798,-2.073935,3.028252\H,0,-6.401446,-3.407035,1.637899
\H,0,-5.562066,-4.598792,-0.378144\H,0,-3.156824,-4.43364,-0.989675\H,
0,-1.607263,-3.089052,0.397127\H,0,-4.938067,0.881511,0.276795\H,0,-6.
034777,0.734683,-1.118059\H,0,-5.943898,3.118105,-0.239734\H,0,-5.3801
83,2.982035,-1.915035\H,0,-3.7527,3.211755,0.658663\H,0,-3.741321,4.30
301,-0.748806\H,0,-4.269235,-1.371828,-1.538111\H,0,-5.274162,-0.52329
1,-2.723992\H,0,-3.377279,-0.181656,-4.217791\H,0,-3.338404,-1.879424,
-3.70978\H,0,-1.653793,-1.343979,-1.959732\H,0,-1.050835,-0.705005,-3.
483718\H,0,-2.75953,-1.193684,3.463548\H,0,-0.854854,1.16483,-2.279488
\C,0,2.936595,-1.441558,0.390332\C,0,1.778042,-2.207906,-0.061012\C,0,
2.749144,-0.413776,1.397732\O,0,0.595936,-2.033721,0.21853\O,0,2.18059
4,-3.219992,-0.865175\O,0,1.777354,0.324517,1.520949\O,0,3.844916,-0.2
82889,2.182338\C,0,1.144359,-4.006838,-1.462952\C,0,1.80988,-5.027412,
-2.361585\C,0,3.798634,0.762418,3.162136\C,0,5.09753,0.712232,3.937802
\H,0,3.839863,-2.032962,0.495428\H,0,0.48016,-3.34221,-2.02466\H,0,0.5
55194,-4.487874,-0.673521\H,0,2.4846,-5.672742,-1.78966\H,0,2.388457,-
4.533189,-3.148468\H,0,1.051473,-5.6589,-2.83732\H,0,2.92783,0.610615,
3.809512\H,0,3.663915,1.725755,2.657193\H,0,5.953008,0.85267,3.269701\
H,0,5.212587,-0.251755,4.443574\H,0,5.112819,1.503355,4.69549\C,0,7.77
3243,0.164153,-0.537219\C,0,7.293821,-1.000685,-1.134641\C,0,5.930734,
-1.154553,-1.368103\C,0,5.016968,-0.150727,-1.014201\C,0,5.513174,1.01
153,-0.406244\C,0,6.876409,1.168323,-0.17365\C,0,3.58115,-0.349838,-1.
319314\C,0,2.756503,0.763041,-1.577341\N,0,1.576529,0.600929,-2.228763
\O,0,1.199326,-0.528325,-2.620787\O,0,0.859085,1.637369,-2.448967\H,0,
8.837802,0.289388,-0.356593\H,0,7.983279,-1.7898,-1.42421\H,0,5.560799
,-2.063996,-1.836817\H,0,4.829714,1.802005,-0.110162\H,0,7.240754,2.08
0821,0.292069\H,0,3.368245,-1.213966,-1.943591\H,0,2.958714,1.777578,-
1.271333\\Version=AM64L-G03RevD.02\State=1-A\HF=-2618.4042425\RMSD=4.5
48e-09\Thermal=0.\Dipole=-1.1092337,0.1222557,-1.2866652\PG=C01 [X(C37
H52N6O6S1)]\\@

Fin15

1\1\FAU-CCC-CCDH160\SP\RB3PW91\6-31G(d)\C37H52N6O6S1\SHUBINA\11-Aug-20
08\0\\#P B3PW91/6-31G(d) SCF=Tight Name=SHUBINA SCRF=(PCM,Solvent=THF)
\\Aza Fin 2R\\0,1\C,0,-1.1825,4.52632,-0.97359\C,0,-1.32904,3.03221,-0
.64866\C,0,-0.98063,2.72517,0.82911\C,0,0.38805,3.32325,1.19792\C,0,0.
51742,4.80176,0.84028\C,0,0.22641,5.02042,-0.64348\N,0,-0.9422,1.29076
,1.06144\C,0,-1.78757,0.58001,1.84757\S,0,-3.20248,1.23348,2.532\N,0,-
1.40277,-0.70109,2.04794\C,0,-2.19147,-1.70924,2.74107\C,0,-3.09822,-2
.48762,1.79584\C,0,-1.25464,-2.63776,3.51817\C,0,-4.47121,-2.56564,2.0
4227\C,0,-5.30656,-3.3098,1.20862\C,0,-4.7762,-3.98628,0.11182\C,0,-3.
40642,-3.90752,-0.14759\C,0,-2.57402,-3.16285,0.6863\N,0,-2.6695,2.522

19,-1.01548\C,0,-2.83445,1.3143,-1.61873\N,0,-4.07115,0.75086,-1.66724
\C,0,-5.14716,1.2581,-0.82411\C,0,-5.14784,2.76763,-0.92088\C,0,-3.799
15,3.27175,-0.44735\C,0,-4.33593,-0.51753,-2.34532\C,0,-3.28996,-0.814
8,-3.40325\C,0,-1.92554,-0.64338,-2.76738\N,0,-1.81669,0.69787,-2.2155
8\H,0,-1.89101,5.1289,-0.38994\H,0,-1.41833,4.68214,-2.03368\H,0,-0.58
051,2.51563,-1.2542\H,0,-1.75212,3.13423,1.49015\H,0,1.16722,2.75987,0
.66889\H,0,0.55698,3.1568,2.26803\H,0,1.52534,5.15444,1.0931\H,0,-0.18
284,5.39943,1.44282\H,0,0.95997,4.47427,-1.25176\H,0,0.31543,6.08209,-
0.90564\H,0,-0.05493,0.84483,0.83077\H,0,-0.55046,-1.02814,1.59248\H,0
,-0.55525,-3.14861,2.84729\H,0,-1.83494,-3.39996,4.04791\H,0,-0.67497,
-2.06514,4.25007\H,0,-4.88937,-2.03062,2.89181\H,0,-6.37273,-3.35992,1
.41742\H,0,-5.4234,-4.57341,-0.53563\H,0,-2.98415,-4.43595,-0.9998\H,0
,-1.50863,-3.09955,0.47465\H,0,-5.00773,0.92984,0.21501\H,0,-6.08298,0
.83347,-1.19971\H,0,-5.92996,3.20214,-0.28958\H,0,-5.34216,3.06879,-1.
95752\H,0,-3.74911,3.19293,0.64573\H,0,-3.68669,4.3214,-0.7297\H,0,-4.
36711,-1.32787,-1.60359\H,0,-5.33392,-0.44276,-2.79471\H,0,-3.39766,-0
.12908,-4.2524\H,0,-3.42595,-1.83614,-3.77407\H,0,-1.76803,-1.38798,-1
.97717\H,0,-1.10255,-0.75438,-3.4743\H,0,-2.82569,-1.16442,3.44621\H,0
,-0.84016,1.07964,-2.24785\C,0,3.06684,-1.25096,0.187\C,0,1.86747,-2.1
6256,-0.02469\C,0,2.84839,-0.31471,1.3465\O,0,0.83701,-2.16909,0.62435
\O,0,2.18835,-3.11458,-0.90882\O,0,1.90935,0.44369,1.48014\O,0,3.86071
,-0.38711,2.22384\C,0,1.15281,-4.05094,-1.24377\C,0,1.74325,-5.05717,-
2.20743\C,0,3.80334,0.52612,3.33642\C,0,5.09367,0.37616,4.11131\H,0,3.
8966,-1.91874,0.44246\H,0,0.32296,-3.4942,-1.68971\H,0,0.79393,-4.5282
1,-0.32554\H,0,2.58479,-5.58845,-1.75113\H,0,2.09715,-4.56353,-3.11769
\H,0,0.9833,-5.79485,-2.48714\H,0,2.9234,0.28622,3.94281\H,0,3.67075,1
.54217,2.95086\H,0,5.95475,0.60641,3.47628\H,0,5.20825,-0.64414,4.4904
1\H,0,5.0973,1.06319,4.96437\C,0,7.74232,0.47761,-0.81735\C,0,7.32675,
-0.67522,-1.48065\C,0,5.96904,-0.97216,-1.58197\C,0,5.00108,-0.12892,-
1.02562\C,0,5.43284,1.02535,-0.36104\C,0,6.78927,1.3275,-0.25841\C,0,3
.53132,-0.49238,-1.1371\C,0,2.70736,0.68079,-1.53782\N,0,1.47428,0.492
03,-1.96597\O,0,0.95088,-0.67329,-1.94653\O,0,0.7763,1.49101,-2.4174\H
,0,8.80049,0.71458,-0.73999\H,0,8.05991,-1.34286,-1.92681\H,0,5.6516,-
1.87107,-2.10691\H,0,4.7002,1.70043,0.07476\H,0,7.10203,2.23284,0.2566
4\H,0,3.42658,-1.2719,-1.90341\H,0,3.08949,1.68789,-1.60767\\Version=A
M64L-G03RevD.02\State=1-A\HF=-2618.4244936\RMSD=9.687e-09\Thermal=0.\D
ipole=-0.7639447,-0.5349354,-0.7582634\PG=C01 [X(C37H52N6O6S1)]\\@

Init16

1\1\FAU-CCC-CCDH153\SP\RB3PW91\6-31G(d)\C37H52N6O6S1\SHUBINA\14-Aug-20
08\0\\#P B3PW91/6-31G(d) SCF=Tight Name=SHUBINA SCRF=(PCM,Solvent=THF)
\\Aza Init 3R\\0,1\C,0,0.644164,4.280816,-1.776562\C,0,0.933253,2.9848
36,-1.007016\C,0,0.551604,1.755751,-1.871165\C,0,-0.930648,1.837936,-2
.265755\C,0,-1.263388,3.153645,-2.971964\C,0,-0.844424,4.358492,-2.127
886\N,0,0.83579,0.506947,-1.200216\C,0,1.735826,-0.418396,-1.623634\S,
0,2.844238,-0.140547,-2.889196\N,0,1.683613,-1.58361,-0.944603\C,0,2.5
18825,-2.742671,-1.218552\C,0,3.750925,-2.796846,-0.325014\C,0,1.68188
5,-4.019689,-1.102255\C,0,5.024664,-2.923944,-0.884454\C,0,6.156064,-3
.024355,-0.074334\C,0,6.028521,-2.99656,1.312653\C,0,4.76159,-2.862943
,1.883424\C,0,3.634345,-2.761942,1.06991\N,0,2.328049,2.904126,-0.5249
99\C,0,2.625273,2.24074,0.621555\N,0,3.917621,1.957127,0.933419\C,0,4.
965992,2.050914,-0.077798\C,0,4.744331,3.300556,-0.89922\C,0,3.363392,
3.230047,-1.516231\C,0,4.256024,1.160523,2.113085\C,0,3.27826,1.419171
,3.243303\C,0,1.877575,1.176506,2.722857\N,0,1.662727,1.939365,1.50018
\H,0,1.215076,4.31532,-2.71352\H,0,0.950052,5.144621,-1.172175\H,0,0.2
75412,3.011675,-0.137885\H,0,1.168789,1.759392,-2.777109\H,0,-1.537999
,1.742782,-1.356644\H,0,-1.165365,0.981499,-2.908061\H,0,-2.337233,3.1
97582,-3.19226\H,0,-0.743816,3.192864,-3.940589\H,0,-1.438273,4.386233
,-1.204072\H,0,-1.046214,5.294011,-2.664381\H,0,0.233805,0.239411,-0.4
1394\H,0,0.971089,-1.660979,-0.216173\H,0,1.361049,-4.192379,-0.068442
\H,0,2.276786,-4.885481,-1.410886\H,0,0.788448,-3.95311,-1.730426\H,0,
5.129177,-2.93557,-1.966895\H,0,7.138687,-3.123014,-0.529776\H,0,6.908
213,-3.080922,1.946239\H,0,4.650811,-2.84833,2.965696\H,0,2.650608,-2.
652303,1.522343\H,0,4.960503,1.155599,-0.715451\H,0,5.923732,2.08971,0
.450807\H,0,5.489621,3.372499,-1.697826\H,0,4.844584,4.186497,-0.26037
3\H,0,3.343542,2.469588,-2.308578\H,0,3.111199,4.19904,-1.951518\H,0,4
.274017,0.09356,1.849851\H,0,5.272373,1.444083,2.40787\H,0,3.38068,2.4

51656,3.598587\H,0,3.495687,0.750587,4.082307\H,0,1.118083,1.504807,3.
438485\H,0,1.697103,0.114357,2.521229\H,0,2.857606,-2.63135,-2.252584\
H,0,0.666978,1.970433,1.237227\C,0,-2.151874,0.057264,2.127099\C,0,-2.
017707,1.411443,1.779004\C,0,-1.208053,-0.950116,1.830669\O,0,-1.08595
9,1.999737,1.18671\O,0,-3.114133,2.146416,2.17091\O,0,-0.140206,-0.862
125,1.187074\O,0,-1.586574,-2.149546,2.368695\C,0,-3.057562,3.548262,1
.928223\C,0,-4.287124,4.170459,2.559247\C,0,-0.758937,-3.275927,2.0989
47\C,0,-1.379067,-4.469937,2.79709\H,0,-3.014822,-0.227344,2.715803\H,
0,-3.027673,3.740226,0.848049\H,0,-2.136683,3.96337,2.354802\H,0,-4.31
1075,3.976831,3.63653\H,0,-5.202169,3.76129,2.118305\H,0,-4.286241,5.2
54896,2.402026\H,0,0.257416,-3.088182,2.469163\H,0,-0.698313,-3.436304
,1.017066\H,0,-2.390727,-4.654917,2.421991\H,0,-1.440793,-4.301829,3.8
77349\H,0,-0.776126,-5.36739,2.619509\C,0,-8.036742,0.052289,0.022802\
C,0,-6.87968,0.514229,0.646417\C,0,-5.648616,-0.063378,0.350711\C,0,-5
.557034,-1.116611,-0.57571\C,0,-6.733754,-1.572548,-1.198545\C,0,-7.95
9214,-0.993006,-0.901707\C,0,-4.240947,-1.682148,-0.841694\C,0,-3.9579
43,-2.681543,-1.69114\N,0,-2.603873,-3.150427,-1.853481\O,0,-2.450846,
-4.111037,-2.606501\O,0,-1.693223,-2.585123,-1.250565\H,0,-8.998931,0.
502101,0.254147\H,0,-6.932517,1.324508,1.368377\H,0,-4.746957,0.298989
,0.838915\H,0,-6.690454,-2.385706,-1.917827\H,0,-8.859885,-1.355843,-1
.38991\H,0,-3.406528,-1.252004,-0.289207\H,0,-4.648926,-3.237486,-2.30
9878\\Version=AM64L-G03RevD.02\State=1-A\HF=-2618.4145171\RMSD=7.961e-
09\Thermal=0.\Dipole=0.7133284,3.0008854,1.8816873\PG=C01 [X(C37H52N6O
6S1)]\\@

TS16

1\1\FAU-CCC-CCDH158\SP\RB3PW91\6-31G(d)\C37H52N6O6S1\SHUBINA\11-Aug-20
08\0\\#P B3PW91/6-31G(d) SCF=Tight Name=SHUBINA SCRF=(PCM,Solvent=THF)
\\Aza_TS1\\0,1\C,0,1.182474,4.580707,-0.093551\C,0,1.327248,3.052331,-
0.018393\C,0,1.292366,2.434242,-1.439763\C,0,0.012951,2.868689,-2.1733
13\C,0,-0.163371,4.386227,-2.206258\C,0,-0.119036,4.971678,-0.795346\N
,0,1.361093,0.988008,-1.403938\C,0,2.365623,0.226649,-1.926461\S,0,3.9
2175,0.839205,-2.248424\N,0,2.007736,-1.048776,-2.156512\C,0,2.919865,
-2.144007,-2.453956\C,0,3.322443,-2.883263,-1.184242\C,0,2.274572,-3.0
71256,-3.486336\C,0,4.672114,-3.033631,-0.85482\C,0,5.055118,-3.721675
,0.297734\C,0,4.086729,-4.264262,1.140414\C,0,2.735422,-4.113825,0.821
896\C,0,2.354437,-3.430052,-0.331151\N,0,2.538102,2.649248,0.73121\C,0
,2.572893,1.49857,1.45435\N,0,3.758817,1.002658,1.889434\C,0,5.020185,
1.495106,1.345619\C,0,4.943914,3.000252,1.240488\C,0,3.771063,3.356123
,0.35214\C,0,3.843225,-0.258029,2.6286\C,0,2.609272,-0.463639,3.482153
\C,0,1.399671,-0.364651,2.580235\N,0,1.441746,0.888182,1.830572\H,0,2.
00955,5.019841,-0.665351\H,0,1.225519,5.001265,0.919254\H,0,0.449754,2
.6994,0.528339\H,0,2.172708,2.77826,-1.994971\H,0,-0.848698,2.411907,-
1.666732\H,0,0.038983,2.454509,-3.187522\H,0,-1.109802,4.640937,-2.699
014\H,0,0.635362,4.837593,-2.812637\H,0,-0.975426,4.604367,-0.215354\H
,0,-0.202468,6.065082,-0.827057\H,0,0.474133,0.504162,-1.259767\H,0,1.
008936,-1.270345,-2.067018\H,0,1.317853,-3.461253,-3.123606\H,0,2.9365
77,-3.917,-3.698572\H,0,2.09202,-2.525854,-4.418469\H,0,5.429679,-2.60
3105,-1.506135\H,0,6.110883,-3.833696,0.534712\H,0,4.38154,-4.806446,2
.036075\H,0,1.972238,-4.538534,1.470008\H,0,1.298073,-3.320874,-0.5696
56\H,0,5.208354,1.040308,0.363578\H,0,5.81247,1.185162,2.033729\H,0,5.
859922,3.405194,0.798368\H,0,4.827986,3.437068,2.239938\H,0,4.008182,3
.106109,-0.68978\H,0,3.577366,4.427613,0.428392\H,0,3.97414,-1.093878,
1.927218\H,0,4.743506,-0.201788,3.250168\H,0,2.566872,0.294257,4.27389
5\H,0,2.646273,-1.449405,3.955843\H,0,0.463381,-0.367068,3.13907\H,0,1
.354528,-1.209358,1.886921\H,0,3.816723,-1.689031,-2.884508\H,0,0.5379
41,1.1636,1.448056\C,0,-3.073785,-0.23561,1.221338\C,0,-2.555555,1.111
721,1.28177\C,0,-2.226942,-1.375985,1.508732\O,0,-1.392293,1.511677,1.
185087\O,0,-3.585724,1.992071,1.421164\O,0,-1.012354,-1.44698,1.41373\
O,0,-2.983612,-2.464493,1.820484\C,0,-3.254766,3.379181,1.478801\C,0,-
4.510367,4.131446,1.868511\C,0,-2.255655,-3.691271,1.96887\C,0,-3.2551
31,-4.782625,2.288515\H,0,-4.100891,-0.330889,1.5518\H,0,-2.885644,3.7
04187,0.498263\H,0,-2.446076,3.533305,2.201716\H,0,-4.866137,3.809056,
2.852161\H,0,-5.310319,3.956246,1.142356\H,0,-4.307965,5.207392,1.9088
99\H,0,-1.517188,-3.57657,2.770869\H,0,-1.70718,-3.895407,1.04277\H,0,
-3.978713,-4.904586,1.475825\H,0,-3.805959,-4.553743,3.206764\H,0,-2.7
35129,-5.736973,2.426657\C,0,-7.691848,-0.275963,-1.008312\C,0,-6.8758

54,0.783776,-1.398437\C,0,-5.490222,0.657757,-1.338244\C,0,-4.893409,-
0.532091,-0.900237\C,0,-5.726476,-1.588428,-0.500101\C,0,-7.109997,-1.
460594,-0.554083\C,0,-3.418974,-0.642124,-0.893453\C,0,-2.803282,-1.84
5646,-1.254646\N,0,-1.487362,-1.888001,-1.635061\O,0,-0.941891,-2.9993
83,-1.812337\O,0,-0.853275,-0.812111,-1.846941\H,0,-8.773706,-0.179767
,-1.052652\H,0,-7.318134,1.712271,-1.751229\H,0,-4.857343,1.487185,-1.
644473\H,0,-5.28375,-2.503544,-0.11754\H,0,-7.739045,-2.288903,-0.2373
11\H,0,-2.881927,0.245492,-1.213132\H,0,-3.25718,-2.821474,-1.168181\\
Version=AM64L-G03RevD.02\State=1-A\HF=-2618.3950746\RMSD=2.919e-09\The
rmal=0.\Dipole=0.7686224,2.7200191,3.5950381\PG=C01 [X(C37H52N6O6S1)]\
\@

Fin16

1\1\FAU-CCC-CCDH161\SP\RB3PW91\6-31G(d)\C37H52N6O6S1\SHUBINA\13-Aug-20
08\0\\#P B3PW91/6-31G(d) SCF=Tight Name=SHUBINA SCRF=(PCM,Solvent=THF)
\\Aza Fin 3R\\0,1\C,0,-1.171522,4.434121,0.387963\C,0,-1.347371,2.9116
24,0.360269\C,0,-1.869496,2.365586,1.71526\C,0,-0.952737,2.836518,2.85
7551\C,0,-0.74739,4.35152,2.863776\C,0,-0.219587,4.841308,1.514785\N,0
,-1.915285,0.925411,1.681021\C,0,-3.0195,0.147184,1.754467\S,0,-4.6079
32,0.775155,1.846937\N,0,-2.734116,-1.171557,1.745885\C,0,-3.706446,-2
.248094,1.823058\C,0,-3.88988,-2.95152,0.483972\C,0,-3.301049,-3.22921
7,2.928171\C,0,-5.151539,-2.994253,-0.117395\C,0,-5.349855,-3.666925,-
1.323886\C,0,-4.282598,-4.308933,-1.948632\C,0,-3.016821,-4.266172,-1.
361161\C,0,-2.819691,-3.591987,-0.156779\N,0,-2.199187,2.468488,-0.766
705\C,0,-1.948986,1.30102,-1.418603\N,0,-2.932875,0.659948,-2.08589\C,
0,-4.322814,1.081321,-1.930932\C,0,-4.363445,2.59353,-1.982069\C,0,-3.
511024,3.128747,-0.848942\C,0,-2.715218,-0.621224,-2.763357\C,0,-1.272
141,-0.785549,-3.20137\C,0,-0.366178,-0.501077,-2.020104\N,0,-0.707332
,0.808121,-1.468606\H,0,-2.135757,4.928893,0.561276\H,0,-0.801013,4.77
6091,-0.587017\H,0,-0.34867,2.49074,0.224547\H,0,-2.889338,2.724203,1.
893751\H,0,0.017803,2.331856,2.758006\H,0,-1.387298,2.496912,3.804565\
H,0,-0.055576,4.629656,3.668414\H,0,-1.700488,4.854897,3.08335\H,0,0.7
75282,4.411704,1.329597\H,0,-0.098649,5.93176,1.518352\H,0,-0.989176,0
.455621,1.741178\H,0,-1.747931,-1.438004,1.585981\H,0,-2.312012,-3.659
187,2.733311\H,0,-4.021915,-4.051272,2.99529\H,0,-3.264469,-2.71207,3.
892527\H,0,-5.986637,-2.492761,0.366953\H,0,-6.340616,-3.691758,-1.772
105\H,0,-4.43473,-4.84191,-2.884363\H,0,-2.177761,-4.766589,-1.839809\
H,0,-1.823716,-3.553716,0.278424\H,0,-4.733117,0.708384,-0.982417\H,0,
-4.88785,0.638378,-2.756141\H,0,-5.387463,2.959595,-1.85673\H,0,-3.995
593,2.941838,-2.955118\H,0,-4.041103,2.974073,0.099023\H,0,-3.340769,4
.200272,-0.988845\H,0,-3.010507,-1.437143,-2.091804\H,0,-3.386996,-0.6
44488,-3.628836\H,0,-1.04457,-0.097664,-4.024824\H,0,-1.118766,-1.8070
89,-3.562692\H,0,0.681589,-0.451923,-2.322157\H,0,-0.463602,-1.261786,
-1.233511\H,0,-4.655627,-1.775519,2.091454\H,0,0.064539,1.318944,-1.05
6747\C,0,3.794149,0.231453,-0.598853\C,0,3.143633,1.517443,-1.088404\C
,0,3.720135,-0.758957,-1.754835\O,0,1.99101,1.858105,-0.91017\O,0,4.02
852,2.260069,-1.759992\O,0,3.07386,-0.592251,-2.768713\O,0,4.467454,-1
.843229,-1.522692\C,0,3.52749,3.478833,-2.345756\C,0,4.67866,4.143522,
-3.066824\C,0,4.440139,-2.863166,-2.544857\C,0,5.32735,-3.996473,-2.08
1556\H,0,4.853347,0.457257,-0.432918\H,0,3.121554,4.10902,-1.547647\H,
0,2.706192,3.225473,-3.023804\H,0,5.071533,3.495643,-3.856427\H,0,5.49
2529,4.377452,-2.373498\H,0,4.338108,5.077973,-3.525334\H,0,4.785775,-
2.422634,-3.485654\H,0,3.403035,-3.182836,-2.687962\H,0,4.973022,-4.40
7612,-1.131551\H,0,6.359876,-3.658598,-1.948546\H,0,5.321818,-4.797345
,-2.828605\C,0,5.865572,-1.712347,3.808002\C,0,4.57279,-1.279736,4.108
614\C,0,3.732063,-0.826994,3.09803\C,0,4.162441,-0.791617,1.762142\C,0
,5.4543,-1.236999,1.471214\C,0,6.299229,-1.69183,2.486414\C,0,3.155791
,-0.281009,0.729414\C,0,2.038974,-1.254819,0.509039\N,0,0.864533,-1.07
6903,1.089458\O,0,-0.096596,-1.91122,0.885787\O,0,0.648503,-0.063769,1
.860587\H,0,6.525284,-2.064034,4.59746\H,0,4.218428,-1.292619,5.136709
\H,0,2.72098,-0.498523,3.328129\H,0,5.810933,-1.255539,0.446062\H,0,7.
300962,-2.0337,2.23554\H,0,2.666338,0.588362,1.180601\H,0,2.162965,-2.
196351,-0.008002\\Version=AM64L-G03RevD.02\State=1-A\HF=-2618.4270041\
RMSD=8.880e-09\Thermal=0.\Dipole=1.5108138,1.7053386,-4.418801\PG=C01
[X(C37H52N6O6S1)]\\@
